# Phosphokinome Analysis of Barth Syndrome Lymphoblasts Identify Novel Targets in the Pathophysiology of the Disease

**DOI:** 10.3390/ijms19072026

**Published:** 2018-07-12

**Authors:** Prasoon Agarwal, Laura K. Cole, Abin Chandrakumar, Kristin D. Hauff, Amir Ravandi, Vernon W. Dolinsky, Grant M. Hatch

**Affiliations:** 1 Department of Pharmacology and Therapeutics, University of Manitoba, Winnipeg, MB R3E 3P4, Canada; pagarwal@chrim.ca (P.A.); lcole@chrim.ca (L.K.C.); chandraa@myumanitoba.ca (A.C.); vdolinsky@chrim.ca (V.W.D.); 2 Diabetes Research Envisioned and Accomplished in Manitoba (DREAM), Children’s Hospital Research Institute of Manitoba, Winnipeg, MB R3E 3P4, Canada; 3 Manitoba Developmental Origins of Chronic Diseases in Children Network (DEVOTION), University of Manitoba, Winnipeg, MB R3E 3P4, Canada; 4 Clinical Research Unit, Children’s Hospital Research Institute of Manitoba, Winnipeg, MB R3E 3P4, Canada; 5 Pathology and Laboratory Medicine, University of British Columbia, Vancouver, BC V6T 2B5, Canada; Kristin.hauff@interiorhealth.ca; 6 Physiology and Pathophysiology, University of Manitoba, St. Boniface Hospital Research Center, Winnipeg, MB R2H 2A6, Canada; aravandi@sbgh.mb.ca; 7 Center for Research and Treatment of Atherosclerosis, University of Manitoba, Winnipeg, MB R3E 3P4, Canada

**Keywords:** cardiolipin, Barth Syndrome, phosphokinome, phosphoproteins, rare genetic disease, phospholipid, human lymphoblasts, mitochondria, cardiomyopathy, neutropenia

## Abstract

Barth Syndrome (BTHS) is a rare X-linked genetic disease in which the specific biochemical deficit is a reduction in the mitochondrial phospholipid cardiolipin (CL) as a result of a mutation in the CL transacylase tafazzin. We compared the phosphokinome profile in Epstein-Barr-virus-transformed lymphoblasts prepared from a BTHS patient with that of an age-matched control individual. As expected, mass spectrometry analysis revealed a significant (>90%) reduction in CL in BTHS lymphoblasts compared to controls. In addition, increased oxidized phosphatidylcholine (oxPC) and phosphatidylethanolamine (PE) levels were observed in BTHS lymphoblasts compared to control. Given the broad shifts in metabolism associated with BTHS, we hypothesized that marked differences in posttranslational modifications such as phosphorylation would be present in the lymphoblast cells of a BTHS patient. Phosphokinome analysis revealed striking differences in the phosphorylation levels of phosphoproteins in BTHS lymphoblasts compared to control cells. Some phosphorylated proteins, for example, adenosine monophosphate kinase, have been previously validated as bonafide modified phosphorylation targets observed in tafazzin deficiency or under conditions of reduced cellular CL. Thus, we report multiple novel phosphokinome targets in BTHS lymphoblasts and hypothesize that alteration in the phosphokinome profile may provide insight into the pathophysiology of BTHS and potential therapeutic targets.

## 1. Introduction

Cardiolipin (CL) is a key phospholipid involved in mitochondrial energy production [1,2,3,4,5,6,7]. Mammalian CL *de novo* biosynthesis occurs via the cytidine-5’-diphosphate-1,2-diacylglycerol pathway. Subsequent to biosynthesis, CL is rapidly remodelled to yield the molecular species of CL found in the mitochondrial membrane [4,5]. For example, in the human heart, linoleic acid (18:2) comprises 80–90% of the acyl chains in CL, the major species (approximately 80% of total) being (18:2-18:2)-(18:2-18:2)-CL or tetralinoleoyl-CL (L_4_-CL) [8]. The transacylation enzyme tafazzin (TAZ) is responsible for the majority of CL remodelling in mammalian tissues [9,10]. Altered remodeling of CL in mitochondria results in oxidative phosphorylation dysfunction [11].

Barth Syndrome (BTHS) is a rare X-linked genetic disorder associated with skeletal muscle abnormalities, cardiomyopathy, and neutropenia and is the only genetic disease in which the specific biochemical defect is a dramatic reduction in CL [12,13,14,15]. The loss of CL is caused by loss-of-function mutations in TAZ. As indicated above, TAZ is the CL transacylase that remodels CL and this is underscored in patients with BTHS where the ability to remodel CL is dramatically reduced [16,17,18]. Loss of CL levels via disruption of TAZ alters assembly/stability of respiratory chain supercomplexes in the mitochondrial inner membrane, promoting mitochondrial bioenergetic dysfunction and increased oxidative stress [19].

Here we examined the phosphokinome profile of Epstein-Barr-virus-transformed lymphoblasts prepared from a BTHS patient and compared it with those of an age-matched control individual. We observed striking differences in the phosphokinome profiles between BTHS and age-matched control lymphoblasts and hypothesize that the novel targets identified may be involved in the pathophysiology of the disease and may represent potential therapeutic targets.

## 2. Results and Discussion

Previous studies have indicated that BTHS lymphoblasts exhibit large reductions in CL levels [20,21,22,23]. The phospholipid profiles of BTHS lymphoblasts and age-matched control lymphoblasts were initially examined. The major phospholipid species are shown in Table 1. Consistent with the previous studies, BTHS lymphoblasts utilized for the phosphokinome analyses also exhibited a large reduction (>90%) in the percentage of CL compared to age-matched control lymphoblasts. In addition, the percent of oxidized-phosphatidylcholine (OxPC) in BTHS lymphoblasts was increased compared to control cells. Previous studies have indicated that TAZ deficiency increases oxidative stress in both yeast and cardiomyocytes, which promotes the generation of biologically potent oxidizing signaling metabolites [24,25]. Thus, PC may possibly serve as a target for increased oxidative modification in BTHS lymphoblasts. BTHS lymphoblasts also exhibited reductions in the percentage of lysophosphatidylcholine (LPC) and lysophosphatidylethanolamine (LPE) compared to controls. The percent of sphingomyelin (SM) was unaltered in BTHS lymphoblasts compared to control. Interestingly, the percents of both phosphatidylethanolamine (PE) and phosphatidylglycerol (PG) were increased in BTHS lymphoblasts compared to control. An increase in PE mass was previously reported in livers of 4-month-old TAZ knockdown mice and in *crd1∆* yeast mutants exhibiting reduced CL [26,27].

A previous study indicated that alterations in various processes involved in protein translation, amino acid metabolism, nucleotide metabolism, GTP hydrolysis, and folate metabolism were observed in TAZ knockdown mice [25]. Given these broad shifts in metabolism, we hypothesized that TAZ deficiency in BTHS would be associated with differences in regulatory posttranslational modifications such as phosphorylation. Hence, the phosphokinome profile of BTHS lymphoblasts was determined using the Kenex^TM^ KAM-880 antibody microarray with phospho-specific antibodies and compared with that of age-matched control lymphoblasts, as described in Material and Methods. The phosphokinone profile of identified targets are presented as pathway maps in Figures S1–S19 with the percent change from control (%CFC), as indicated in Table S1. We chose to discuss only the most significantly altered phosphorylated targets for each pathway map where phosphorylation was >2-fold or reduced by at least 50%. In addition, we focused on the identified phosphokinome targets which have known pathophysiological associations with the disease [28]. Known phosphokinome targets localized to mitochondria are indicated in Table 2.

Pathway map analysis of the phosphokinome profile of proteins involved in the Lipid Metabolism pathway revealed a striking >2-fold increase in pan-specific phosphorylated adenosine monophosphate kinase subunit b (AMPKb(Ps)) compared to controls indicating activation of AMPK in BTHS lymphoblasts (Table S1, Figure S1). AMPK is a master regulator of cellular energy metabolism that activates energy-producing pathways while downregulating anabolic processes in response to low energy availability. Previous studies demonstrated that phosphorylated AMPK is elevated in BTHS lymphoblasts and by short hairpin RNA knockdown of tafazzin in neonatal ventricular myocytes [29,30,31]. Thus, these findings as well as our findings suggest that TAZ deficiency causes reduced energy availability that could serve as a signal that stimulates AMPK in the BTHS lymphoblasts. In the heart, AMPK also modulates the mTOR pathway [32], which is consistent with the marked reduction in mTOR phosphorylation and the protein translation pathway (described below, e.g., Figures S3 and S6). The adaptations in these signaling pathways could be central to alterations in protein translation and amino acid metabolism that are associated with TAZ deficiency. In contrast, a 50% reduction in the phosphorylation of focal adhesion kinase Y756 (FAK(Y756)) was observed in BTHS lymphoblasts compared to control cells. FAK is a nonreceptor protein-tyrosine kinase implicated in signaling pathways involved in cell motility, proliferation, and apoptosis and is known to regulate neutrophil activation and recruitment [33].

When we analyzed the phosphokinome profile of proteins involved in the Glucose Metabolism pathway, we determined a >3-fold increase in pan-specific phosphorylation of protein phosphatase 6C (PP6C(Ps)) and in insulin receptor/insulin-like growth factor-1 receptor (IR/IGF1R (INSR) (Y1189/Y1190)) phosphorylation (Table S1, Figure S2). PP6C is a component of signaling pathways regulating cell cycle progression [34] and IGF1R is involved in cardiomyocyte reprogramming [35]. Notably, we also determined a 63% reduction in the phosphorylation of glycogen synthase kinase-3 a/b Serine 21/Serine 9 (GSK3a/b(S21/S9)) in BTHS lymphoblasts compared to control cells. Inactivation of GSK-3 may be one of the mechanisms that modulate apoptosis in stimulated neutrophils [36].

Pathway map analysis of the phosphokinome profile of proteins involved in the Inflammation pathway indicated that the pan-specific phosphorylation of interleukin-1 receptor-associated kinase 4 (IRAK4 (Ps)) was increased by >2-fold (Table S1, Figure S3). IRAK4 activity is necessary for activating signal pathways which lead to mitogen-activated protein kinases (MAPK) or Toll-like receptor-mediated immune responses [37]. The largest reduction (50%) was in the pan-specific phosphorylation of mammalian target of rapimycin (mTOR (FRAP) (Ps)), which is a key regulator of mammalian skeletal muscle myogenesis [38].

When we performed a pathway map analysis of the phosphokinome profile of proteins involved in the Chaperone pathway, we observed a >2.5-fold increase in pan-specific phosphorylation of a highly conserved protein in the heat shock protein 110 subfamily, APG-1 (APG1 (Ps)) (Table S1, Figure S4). This is a stress-induced protein in response to osmotic imbalance, which is also elevated in hypertensive heart tissues [39]. Interestingly, this was paired with an almost complete reduction in phosphorylation of heat shock protein 27 (HSP27(S78)), which is significantly decreased in a canine model of congestive heart failure with atrial fibrillation [40,41].

Pathway map analysis of the phosphokinome profile of proteins involved in the Transport pathway revealed a 2-fold increase in cortactin (Y470) (Table S1, Figure S5). Cortactin was shown to coimmunoprecipitate with the voltage-gated potassium channel Kv1.5 and aberrant Kv1.5 channel function is hypothesized to contribute to arrhythmogenesis in N-cadherin conditional knockout mice [42]. In contrast, caveolin-2 (S23) phosphorylation was reduced 75%. Caveolin-2 regulates the dynamics of caveolae assembly in mammalian cells [43].

The phosphokinome profile of proteins involved in the protein Translation pathway indicated a >3.7-fold increase in translation initiation factor eIF2Be (S540) (Table S1, Figure S6), which may be involved in promoting beta-adrenergic cardiac myocyte hypertrophy [44]. Since neutropenia is a primary clinical feature of BTHS, it was of interest to identify a 31% reduction in pan-specific phosphorylation of proliferating cell nuclear antigen (PCNA). It is established that PCNA is a nuclear factor involved in DNA replication and repair of proliferating cells and is a key regulator of neutrophil survival [45].

We have also generated a pathway map analysis of the phosphokinome profile of proteins involved in the Apoptosis pathway. Our analysis revealed a 3.7-fold increase in pan-specific phosphorylation of death-associated, protein-related apoptotic kinase-2 (DRAK2) (Table S1, Figure S7), which is highly expressed in lymphoid organs and is a negative regulator of T cell activation [46]. On the other end of the spectrum, pan-specific phosphorylation of the death-associated protein kinase, DAXX, was reduced 73%. Since death-associated protein kinase 2 is upregulated during normal myeloid differentiation and enhances neutrophil maturation in myeloid leukemic cells [47], it may also help us to further understand neutropenia in BTHS.

The Cell growth/division phosphokinome profile revealed a 3.5-fold increase in pan-specific phosphorylation of cell division cycle 25C phosphatase (Cdc25C) (Table S1, Figure S8). The Cdc25C phosphatase is a key activator of Cdc2/cyclin B that controls M-phase entry in eukaryotic cells [48]. In contrast, pan-specific phosphorylation of CDK6 was reduced 65%. CDK6 plays a key role in promoting cell proliferation through promoting progression of cells into the DNA synthesis phase of the cell cycle [49].

Pathway map analysis of the phosphokinome profile of proteins involved in the Adhesion pathway revealed a 2-fold increase in phosphorylation of lymphocyte-specific protein-tyrosine kinase (Lck) (S157) (Table S1, Figure S9). Lck is a member of the Src family of kinases and is required for T cell activation [50]. Moreover, when the phosphokinome profile of proteins involved in the Heme pathway were examined, a 59% decrease in Y1054/Y1059 phosphorylation of vascular endothelial growth factor receptor-tyrosine kinase 2 (Flk1) was observed (Table S1, Figure S10). The Flk1 signaling cascade is involved in cardiovascular system formation, including heart development, hematopoiesis, vasculogenesis, angiogenesis, and endothelial survival [51,52].

Interestingly, the phosphokinome profiles of several proteins involved in the Neuronal pathway were modified in the BTHS lymphoblasts, for example, a 2-fold increase in phosphorylation of Disabled-1 (Dab1(Y198)) (Table S1, Figure S11). Dab1 tyrosine phosphorylation is essential for Reelin signaling [53,54]. In contrast, phosphorylation of Synapsin 1 isoform Ia (S605) was reduced 54%. Synapsins are a family of presynaptic terminal phosphoproteins that modulate synaptic plasticity of the visual cortex [55,56]. Interestingly, we have recently observed reduced expression of synaptophysin, a presynaptic vesicle protein, in the brain of TAZ knockdown mice (unpublished data).

Our analysis also revealed changes in the phosphokinome of BTHS lymphoblasts in the Adrenergic Receptor pathway. For example, we report a 3-fold increase in phosphorylation of arrestin beta 1 (S412) (Table S1, Figure S12). The arrestins are scaffolding proteins involved in regulating signaling cascades involved in development [57]. Arrestin beta 1 acts as a cofactor in the beta-adrenergic receptor kinase (BARK)-mediated desensitization of beta-adrenergic receptors. It is expressed at high levels in peripheral blood leukocytes, and plays a major role in regulating receptor-mediated immune functions [58].

With respect to the phosphokinome profile of proteins involved in regulating Calcium homeostasis, a 2.6-fold increase in pan-specific phosphorylation of cAMP-dependent protein serine-kinase (PKA Ca/b) was observed in the BTHS cells compared to controls (Table S1, Figure S13). Soluble cAMP-dependent protein serine-kinase may also regulate mitochondrial oxidative phosphorylation [59]. In the phosphokinome profile of proteins involved in regulating AKT a 70% reduction in pan-specific phosphorylation of phosphatidylinositol-1,3,5-triphosphate phosphatase and tensin homologue (PTEN) were observed in the BTHS cells compared to controls (Table S1, Figure S14).

The phosphokinome profile of proteins involved in the mitogen-activated protein kinase (MAPK) pathway revealed a 2.3-fold increase in pan-specific phosphorylation of MEK4 (Table S1, Figure S15) and a 74% reduction in pan-specific phosphorylation of MnK2 in the BTHS lymphoblasts. MEK4 may play a role in retinotectal development [60]. MnK2 is activated by ERK, is involved in cell migration [61], and phosphorylates the translation initiation factor eIF4E, which could explain the enhanced phosphorylation of eIF4E in BTHS lymphoblasts (Figure S6).

Possibly related to mitochondrial dysfunction in BTHS cells, pathway map analysis of the phosphokinome profile of proteins involved in the cAMP response element binding protein (CREB) pathway revealed a 2.2-fold increase in phosphorylation of CREB1(S133) and a 51% reduction in pan-specific phosphorylation of calcium/calmodulin-dependent protein-serine kinase 1 gamma (CaMK1g) (Table S1, Figure S16). CREB function is important for the maintenance of normal physiological cardiac function, and in a number of tissues, regulates the expression of peroxisome proliferator-activated receptor-γ coactivator-1, which regulates mitochondrial biogenesis and oxidative metabolism [62]. CaMK1 is also implicated in a signaling pathway by which increases in cytosolic calcium lead to mitochondrial biogenesis in skeletal muscle [63].

Analysis of the phosphokinome profile of proteins involved in the Jun N-terminus protein-serine kinases (JUN) pathway revealed a 2.6-fold increase in phosphorylation of JNK (T183 + Y185) (Table S1, Figure S17). JNK is part of the mitogen-activated MAPK signaling cascade and JNK is phosphorylated and activated in response to various cell stressors [64,65]. In addition, a 50% reduction in phosphorylation of NF-κB p50 nuclear transcription factor (NF-κB) (S276) was observed in the BTHS lymphoblasts (Table S1, Figure S18). Unphosphorylated nuclear NF-κB can also affect the expression of genes not normally regulated by NF-κB through epigenetic mechanisms [66]. This might be a response to oxidative stress due to elevated ROS, as revealed by elevated oxPC.

Finally, pathway map analysis of the phosphokinome profile of proteins involved in the Orphan receptor pathway revealed a 2.7-fold increase in phosphorylation of crystallin αB (heat-shock 20 kDa like-protein) (S45) and a 61% reduction in pan-specific phosphorylation of Fes/Fps protein-tyrosine kinase (Fes) (Table S1, Figure S19). αB-crystallin is essential for muscle differentiation and regulation of αB-crystallin is important for proper myogenesis [67]. αB-crystallin undergoes phosphorylation under stress conditions such as hyperthermia and oxidative stress [68]. Fes regulates leukocyte recruitment and neutrophil chemotaxis [69,70].

In summary, we report for the first time that BTHS lymphoblasts, which exhibit a 90% reduction in CL and increased oxPC, express a markedly distinct phosphokinome profile compared to age-matched control cells. We hypothesize that the phosphokinome profile of BTHS cells may provide novel insight into the pathophysiology of BTHS and identify potential therapeutic targets.

## 3. Materials and Methods

Age-matched control Epstein-Barr-virus-transformed lymphoblasts and BTHS lymphoblasts (Exon 2, c. 171 del. A, frameshift) were a generous gift from Richard Kelly (Johns Hopkins University, Baltimore, MD, USA). RPMI 1640 media and other cell culture components and supplements were from Life Technologies Inc. (Burlington, ON, Canada). Cells were grown in RPMI 1640 medium containing 10% FBS and 1% antibiotic/antimycotic (A/A) solution at 37 °C and 5% CO_2_ in a Thermoscientific Steri-cycle CO_2_ incubator HEPA Class 200 (Winnipeg, Manitoba, Canada). Cell media was changed every 48 h and the cells were passaged every five days until mass spectrometry or phosphokinome analysis was performed. Mass spectrometry of phospholipids in control or BTHS lymphoblasts was performed in triplicate exactly as described [71].

Phosphokinome analysis was performed in duplicate by Kinexus Bioinformatics Corporation (Vancouver, British Columbia, Canada) using 50 μg of lysate protein from control or BTHS lymphoblasts using Kenex^TM^ KAM-880 antibody microarray with phospho-specific antibodies as indicated on the Kinexus website (http://www.kinexus.ca). The KAM-880 chip uses 877 distinct antibodies (~518 pan-specific and ~359 phosphosite-specific) for protein kinases and other cell signaling proteins. Data was expressed as average percent change from the control sample (%CFC) and target proteins were analyzed using the Kinections Pathway Map analysis feature to determine the category of protein family. PhosphoSitePlus^®^ (https://www.phosphosite.org) was used to find the information regarding the protein posttranslational modifications (PTMs).

## Figures and Tables

**Table 1:** Percent phospholipid composition of control and BTHS lymphoblasts.

Phospholipid	BTHS	Control
PC	69.66615	69.75617
OxPC	0.392307	0.264211
LPC	0.161851	0.332797
SM	4.488527	4.887775
CL	0.030898	0.847094
PS	12.26267	14.17647
PG	3.047165	2.288862
LPE	0.019736	0.045338
PE	9.930699	7.401288

Mass spectrometry of phospholipids in control or BTHS lymphoblasts was performed as described in Material and Methods. The individual molecular species of phospholipids are indicated. OxPC, oxidized phosphatidylcholine; PC, phosphatidylcholine; LPC, lysophosphatidylcholine; SM, sphingomyelin; CL, cardiolipin; PS, phosphatidylserine; PG, phosphatidylglycerol; LPE, lysophosphatidylethanolamine; PE, phosphatidylethanolamine.

**Table 2:** Phosphokinome targets localized to mitochondria.

Pathway	Target Protein Name	%CFC
Lipid metabolism	CASP1	27
	AcCoA carboxylase	18
Glucose metabolism	PP6C	182
	PCK2	−34
	PyDK2 (PDHK2)	−36
	GSK3a/b	−61
Inflammation	mTOR (FRAP)	−46
	STAT3	−25
Chaperone	Hsp60	−58
Translation	PP6C	182
Apoptosis	Bad	94
	PTEN	−70
Cell growth & division	CDK1/2	−64
Neuro	Tyrosine hydroxylase	−40
Calcium	PKA Ca/b	163
	PTP1D	−58
CREB	CREB	122
Jun	JNK	161
	JNK2	−24
NF-κB	NF-κB p50	−49
Orphan	Crystallin αB	276

Phosphokinome analysis was performed on control and BTHS lymphoblasts using Kenex^TM^ KAM-880 antibody microarray with phospho-specific antibodies as described in Materials and Methods. Known mitochondrial target proteins with the highest average percent change from the control sample (%CFC) are indicated. Site specific phosphorylation and protein identification of all targets are indicated in Table S1.

## Supplementary Materials

The following are available online at http://www.mdpi.com/1422-0067/19/7/2026/s1, Figure S1: Lipid Metabolism, Figure S2: Glucose Metabolism, Figure S3: Inflammation, Figure S4: Chaperone, Figure S5: Transport, Figure S6: Translation, Figure S7: Apoptosis, Figure S8: Cell Growth/Division, Figure S9: Adhesion, Figure S10: Heme, Figure S11: Neuronal, Figure S12: Adrenergic Receptor, Figure S13: Calcium, Figure S14: AKT, Figure S15: MAPK, Figure S16: CREB, Figure S17: JUN, Figure S18: NF-κB, Figure S19: Orphan, Table S1: Phosphokinome profile of control and BTHS lymphoblasts.
