# Supplementary material for: Phosphokinome Analysis of Barth Syndrome Lymphoblasts Identify Novel Targets in the Pathophysiology of the Disease
Source: Int J Mol Sci. 2018 Jul 12;19(7):2026. doi: 10.3390/ijms19072026 (PMC6073761; doi:10.3390/ijms19072026)
Supplement: Supplementary file 1 [file ijms-19-02026-s001.pdf]

Legend to Supplementary Figures and Table

Supplementary Figures 1-19. Pathway map analysis of the phosphokinome profile of control and BTHS lymphoblasts with pathways indicated.

Phosphokinome analysis was performed on control and BTHS lymphoblasts using Kenex^{TM} KAM-880 antibody microarray with phospho-specific antibodies as described in Materials and Methods. Target proteins and average percent change from the control sample (%CFC) are indicated. Site specific phosphorylation and protein identification are indicated in Supplementary Table 1.

Supplementary Table 1. Phosphokinome profiles of control and BTHS lymphoblasts.

Phosphokinome analysis was performed on control and BTHS lymphoblasts using Kenex^{TM} KAM-880 antibody microarray with phospho-specific antibodies as described in Materials and Methods. Data was expressed as average percent change from the control sample (%CFC) and is indicated on the right.

Figure S1

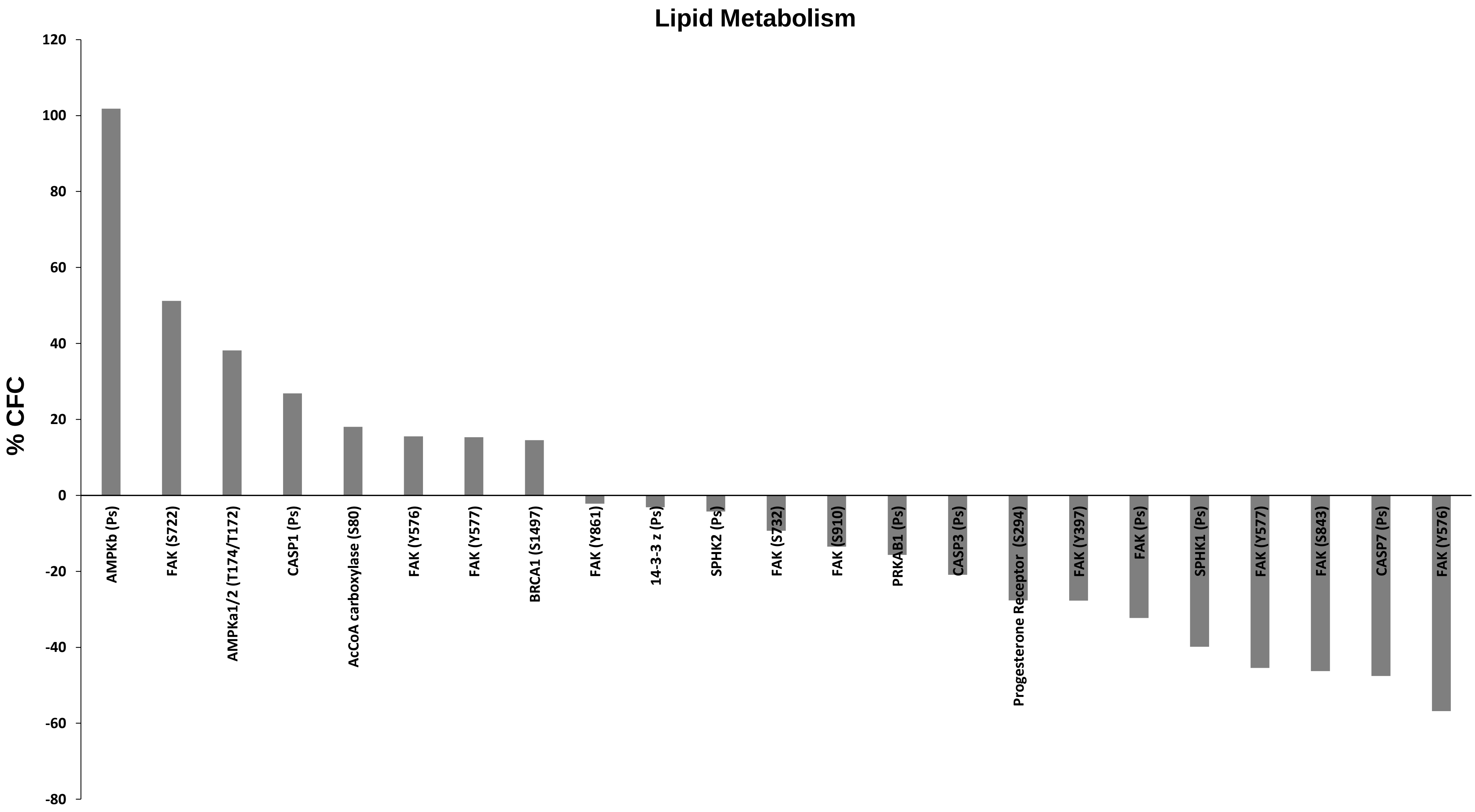

Lipid Metabolism
% CFC
120
100
80
60
40
20
0
-20
-40
-60
-80
AMPKb (Ps)
FAK (S722)
AMPKa1/2 (T174/T172)
CASP1 (Ps)
AcCoA carboxylase (S80)
FAK (Y576)
FAK (Y577)
BRCA1 (S1497)
FAK (Y861)
14-3-3 z (Ps)
SPHK2 (Ps)
FAK (S732)
FAK (S910)
PRKAB1 (Ps)
CASP3 (Ps)
Progesterone Receptor (S294)
FAK (Y397)
FAK (Ps)
SPHK1 (Ps)
FAK (Y577)
FAK (S843)
CASP7 (Ps)
FAK (Y576)

Figure S2

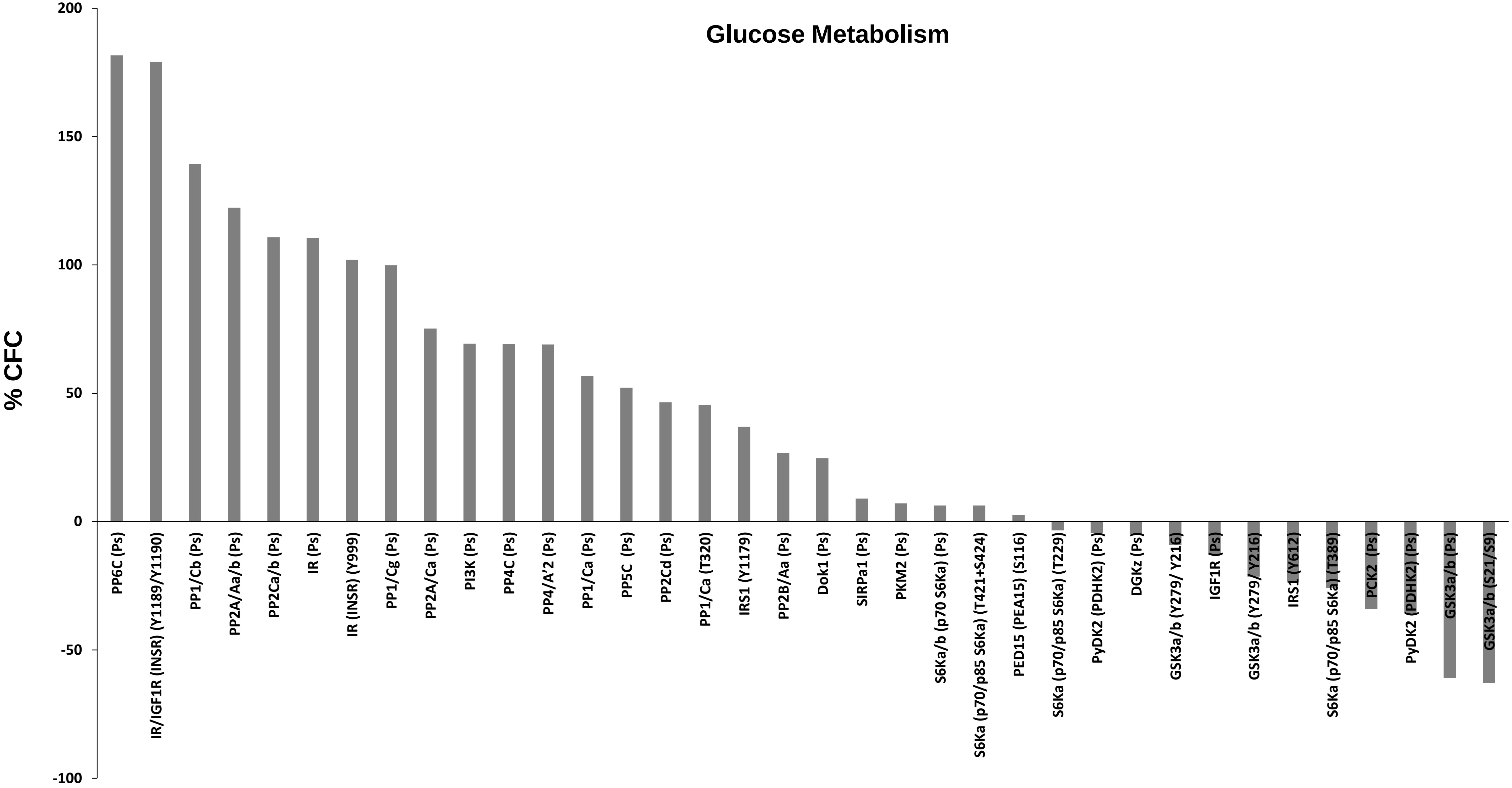
Glucose Metabolism
% CFC
200
150
100
50
0
-50
-100
PP6C (Ps)
IR/IGF1R (INSR) (Y1189/Y1190)
PP1/Cb (Ps)
PP2A/Aa/b (Ps)
PP2Ca/b (Ps)
IR (Ps)
IR (INSR) (Y999)
PP1/Cg (Ps)
PP2A/Ca (Ps)
PI3K (Ps)
PP4C (Ps)
PP4/A'2 (Ps)
PP1/Ca (Ps)
PP5C (Ps)
PP2Cd (Ps)
PP1/Ca (T320)
IRS1 (Y1179)
PP2B/Aa (Ps)
Dok1 (Ps)
SIRPa1 (Ps)
PKM2 (Ps)
S6Ka/b (p70 S6Ka) (Ps)
S6Ka (p70/p85 S6Ka) (T421+S424)
PED15 (PEA15) (S116)
S6Ka (p70/p85 S6Ka) (T229)
PyDK2 (PDHK2) (Ps)
DGKz (Ps)
GSK3a/b (Y279/ Y216)
IGF1R (Ps)
GSK3a/b (Y279/ Y216)
IRS1 (Y612)
S6Ka (p70/p85 S6Ka) (T389)
PCK2 (Ps)
PyDK2 (PDHK2) (Ps)
GSK3a/b (Ps)
GSK3a/b (S21/S9)

Figure S3

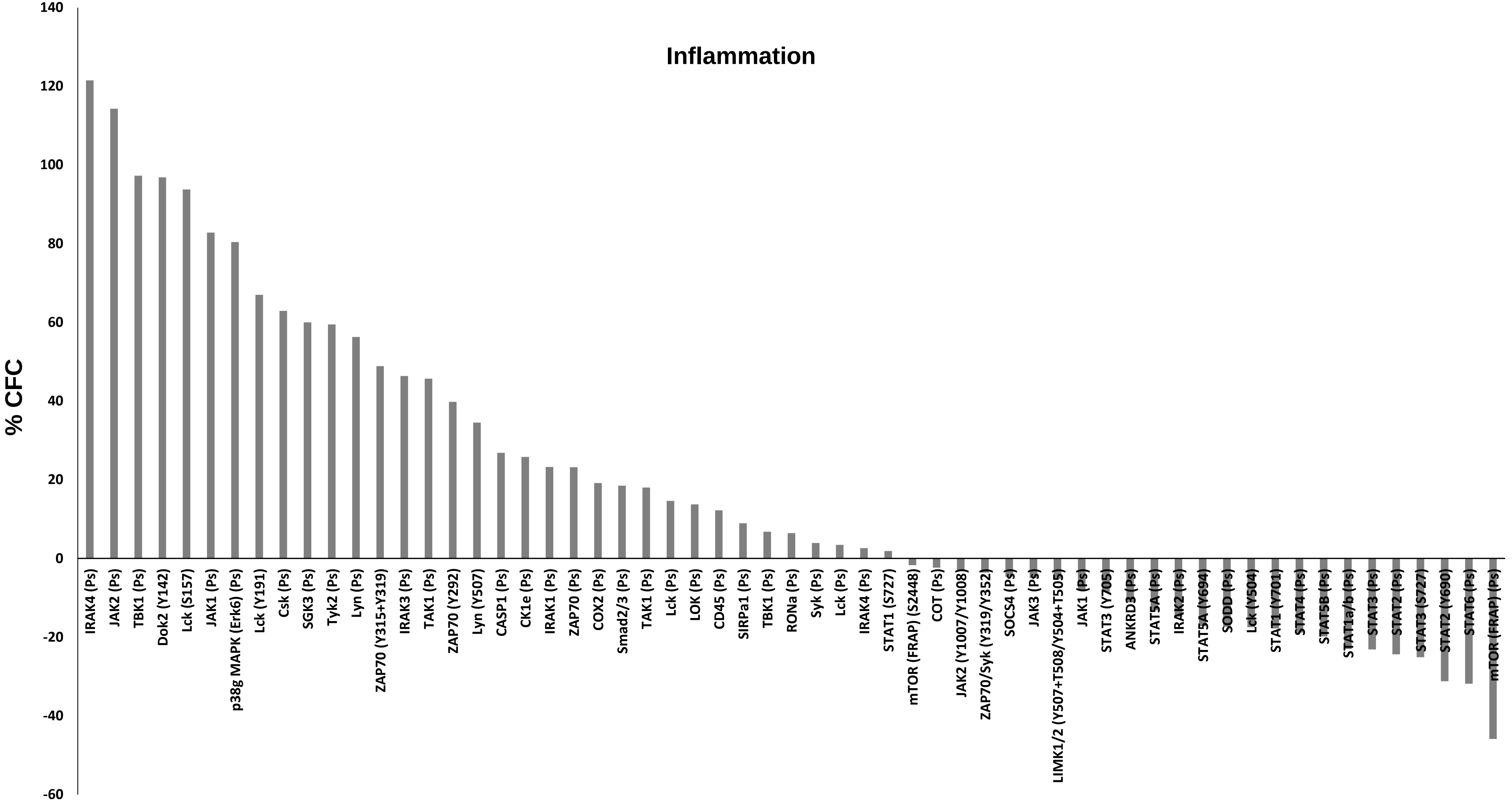
Inflammation
% CFC
140
120
100
80
60
40
20
0
-20
-40
-60
IRAK4 (Ps)
JAK2 (Ps)
TBK1 (Ps)
Dok2 (Y142)
Lck (S157)
JAK1 (Ps)
p38g MAPK (Erk6) (Ps)
Lck (Y191)
Csk (Ps)
SGK3 (Ps)
Tyk2 (Ps)
Lyn (Ps)
ZAP70 (Y315+Y319)
IRAK3 (Ps)
TAK1 (Ps)
ZAP70 (Y292)
Lyn (Y507)
CASP1 (Ps)
CK1e (Ps)
IRAK1 (Ps)
ZAP70 (Ps)
COX2 (Ps)
Smad2/3 (Ps)
TAK1 (Ps)
Lck (Ps)
LOK (Ps)
CD45 (Ps)
SIRPa1 (Ps)
TBK1 (Ps)
RONa (Ps)
Syk (Ps)
Lck (Ps)
IRAK4 (Ps)
STAT1 (S727)
mTOR (FRAP) (S2448)
COT (Ps)
JAK2 (Y1007/Y1008)
ZAP70/Syk (Y319/Y352)
SOCS4 (Ps)
JAK3 (Ps)
LIMK1/2 (Y507+T508/Y504+T505)
JAK1 (Ps)
STAT3 (Y705)
ANKRD3 (Ps)
STAT5A (Ps)
IRAK2 (Ps)
STAT5A (Y694)
SODD (Ps)
Lck (Y504)
STAT1 (Y701)
STAT4 (Ps)
STAT5B (Ps)
STAT1a/b (Ps)
STAT3 (Ps)
STAT2 (Ps)
STAT3 (S727)
STAT2 (Y690)
STAT6 (Ps)
mTOR (FRAP) (Ps)

Figure S4

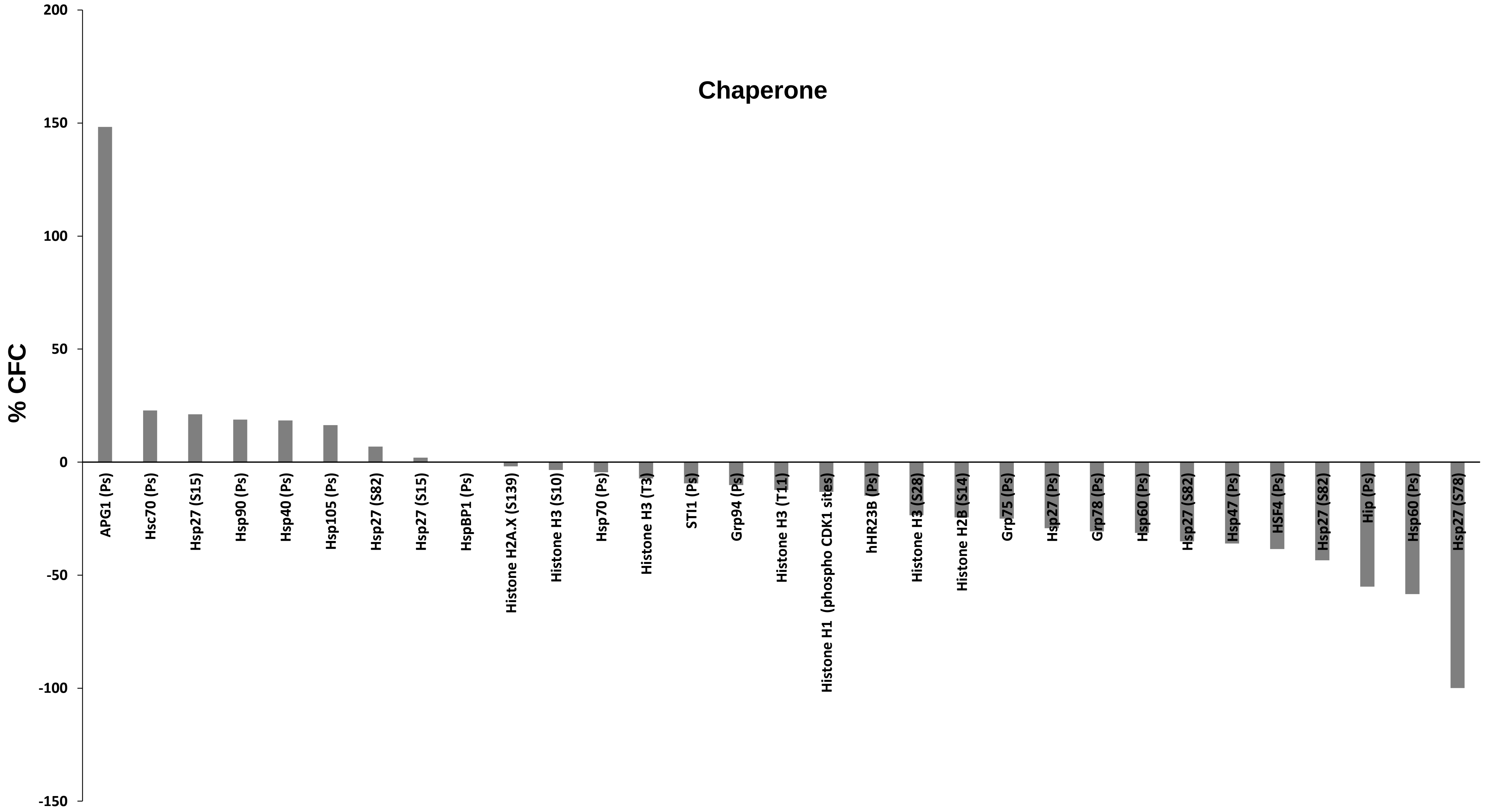

Chaperone
% CFC
200
150
100
50
0
-50
-100
-150
APG1 (Ps)
Hsc70 (Ps)
Hsp27 (S15)
Hsp90 (Ps)
Hsp40 (Ps)
Hsp105 (Ps)
Hsp27 (S82)
Hsp27 (S15)
HspBP1 (Ps)
Histone H2A.X (S139)
Histone H3 (S10)
Hsp70 (Ps)
Histone H3 (T3)
STI1 (Ps)
Grp94 (Ps)
Histone H3 (T11)
Histone H1 (phospho CDK1 sites)
hHR23B (Ps)
Histone H3 (S28)
Histone H2B (S14)
Grp75 (Ps)
Hsp27 (Ps)
Grp78 (Ps)
Hsp60 (Ps)
Hsp27 (S82)
Hsp47 (Ps)
HSF4 (Ps)
Hsp27 (S82)
Hip (Ps)
Hsp60 (Ps)
Hsp27 (S78)

Figure S5

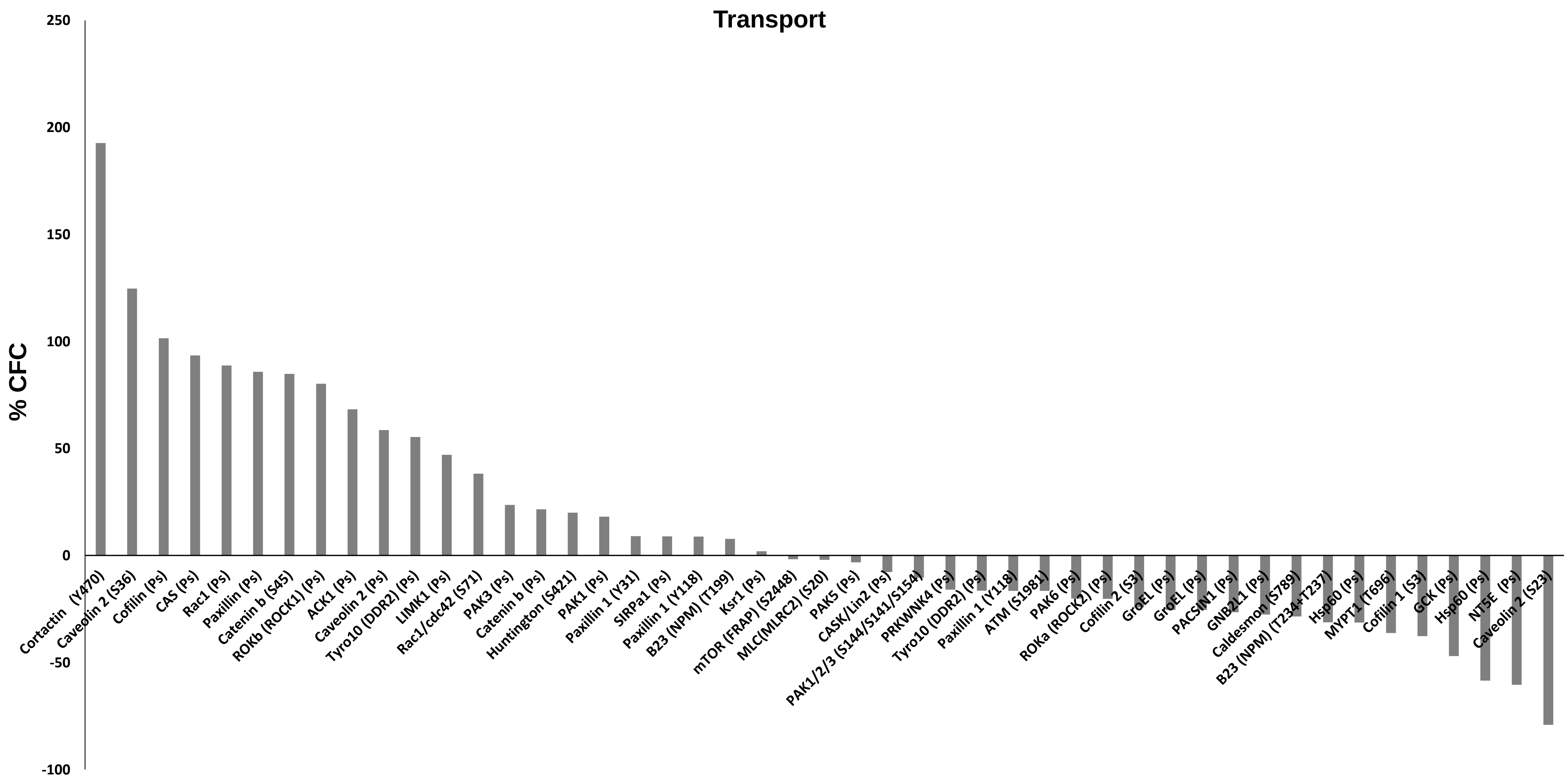

Transport
% CFC
250
200
150
100
50
0
-50
-100
Cortactin (Y470)
Caveolin 2 (S36)
Cofilin (Ps)
CAS (Ps)
Rac1 (Ps)
Paxillin (Ps)
Catenin b (S45)
ROKb (ROCK1) (Ps)
ACK1 (Ps)
Caveolin 2 (Ps)
Tyro10 (DDR2) (Ps)
LIMK1 (Ps)
Rac1/cdc42 (S71)
PAK3 (Ps)
Catenin b (Ps)
Huntington (S421)
PAK1 (Ps)
Paxillin 1 (Y31)
SIRPa1 (Ps)
Paxillin 1 (Y118)
B23 (NPM) (T199)
Ksr1 (Ps)
mTOR (FRAP) (S2448)
MLC(MLRC2) (S20)
PAK5 (Ps)
CASK/Lin2 (Ps)
PAK1/2/3 (S144/S141/S154)
PRKWNK4 (Ps)
Tyro10 (DDR2) (Ps)
Paxillin 1 (Y118)
ATM (S1981)
PAK6 (Ps)
ROKa (ROCK2) (Ps)
Cofilin 2 (S3)
GroEL (Ps)
GroEL (Ps)
PACSIN1 (Ps)
GNB2L1 (Ps)
Caldesmon (S789)
B23 (NPM) (T234+T237)
Hsp60 (Ps)
MYPT1 (T696)
Cofilin 1 (S3)
GCK (Ps)
Hsp60 (Ps)
NT5E (Ps)
Caveolin 2 (S23)

Figure S6

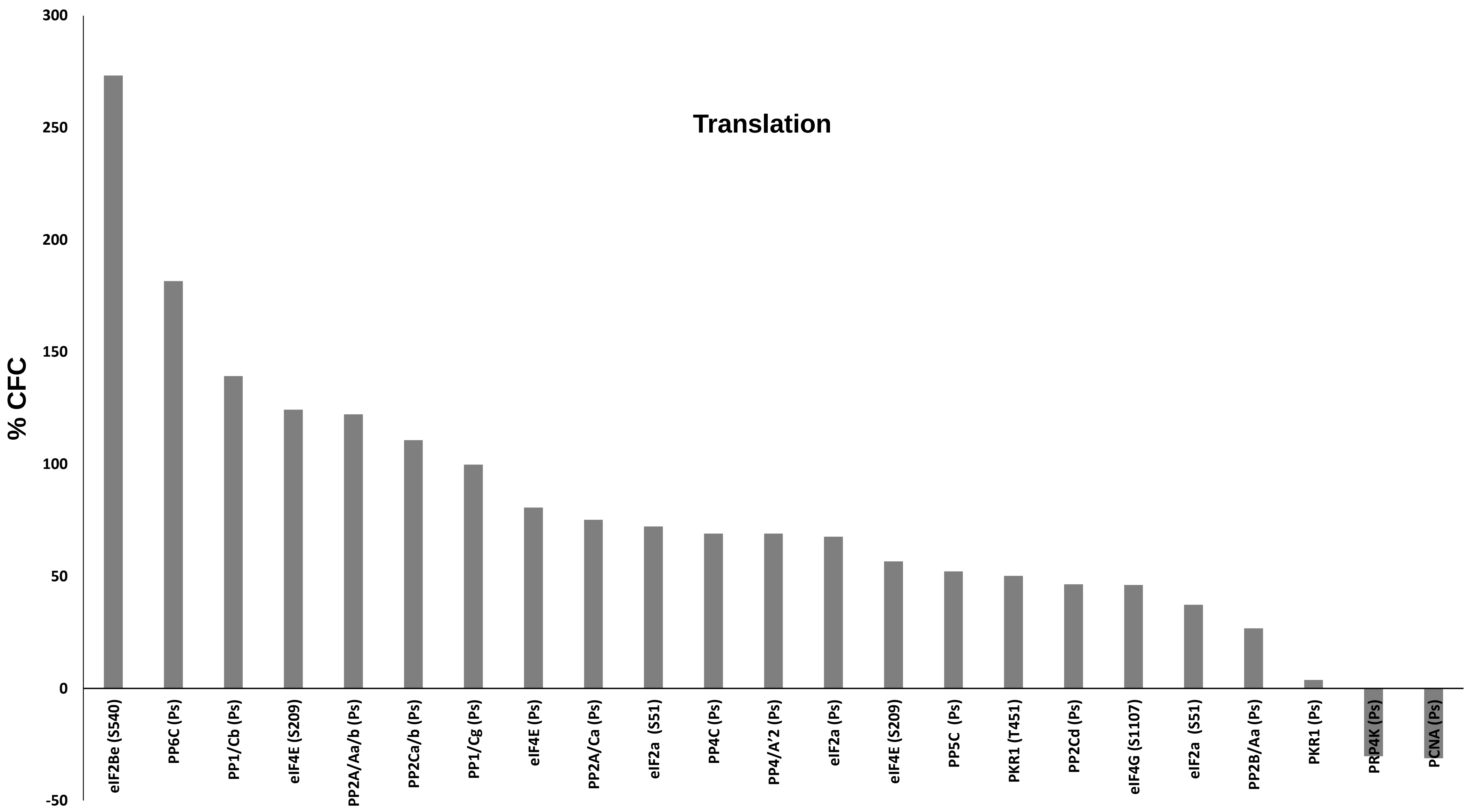

Translation
% CFC
300
250
200
150
100
50
0
-50
eIF2Be (S540)
PP6C (Ps)
PP1/Cb (Ps)
eIF4E (S209)
PP2A/Aa/b (Ps)
PP2Ca/b (Ps)
PP1/Cg (Ps)
eIF4E (Ps)
PP2A/Ca (Ps)
eIF2a (S51)
PP4C (Ps)
PP4/A'2 (Ps)
eIF2a (Ps)
eIF4E (S209)
PP5C (Ps)
PKR1 (T451)
PP2Cd (Ps)
eIF4G (S1107)
eIF2a (S51)
PP2B/Aa (Ps)
PKR1 (Ps)
PRP4K (Ps)
PCNA (Ps)

Figure S7

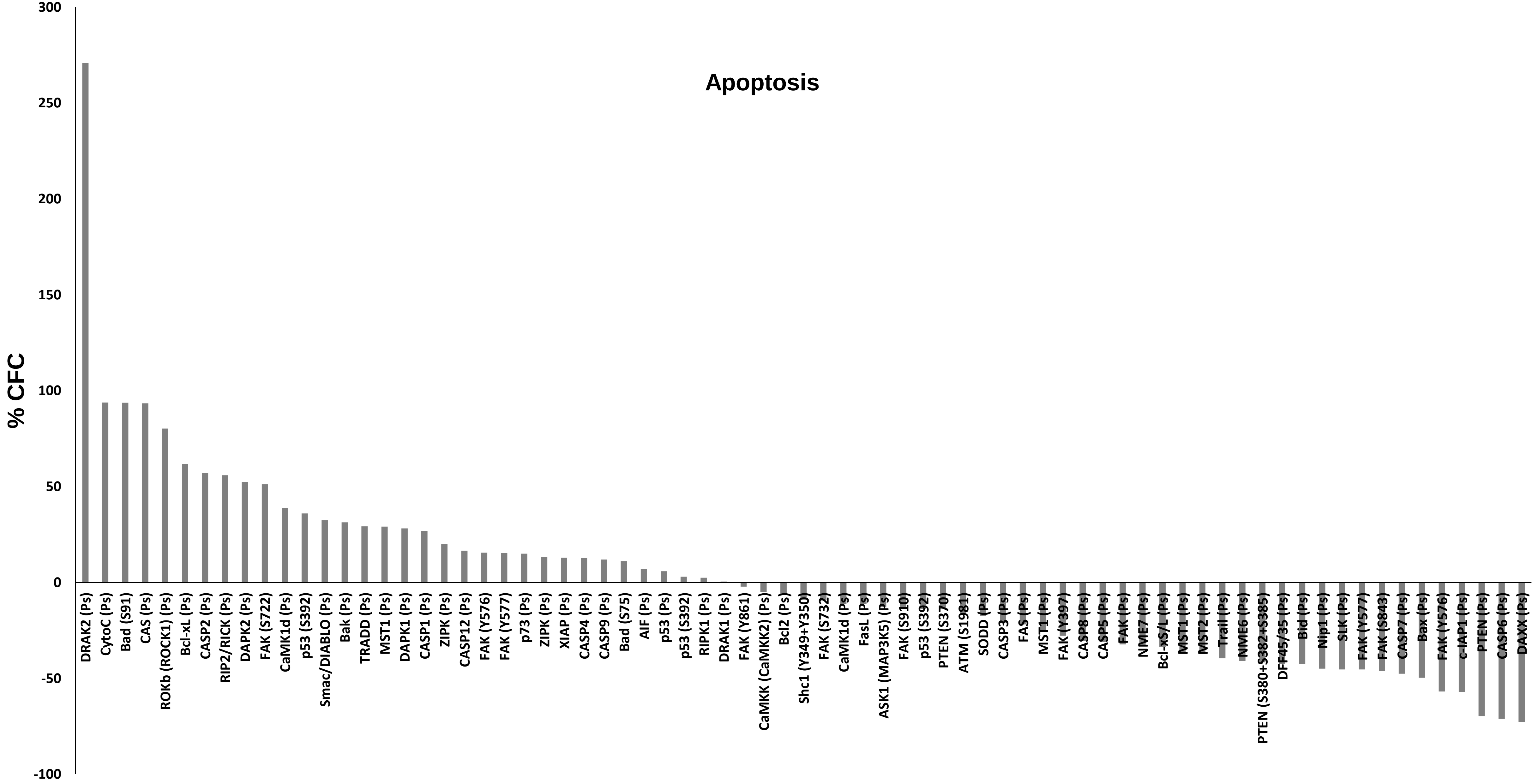
Apoptosis
% CFC
300
250
200
150
100
50
0
-50
-100
DRAK2 (Ps)
CytoC (Ps)
Bad (S91)
CAS (Ps)
ROKb (ROCK1) (Ps)
Bcl-xL (Ps)
CASP2 (Ps)
RIP2/RICK (Ps)
DAPK2 (Ps)
FAK (S722)
CaMK1d (Ps)
p53 (S392)
Smac/DIABLO (Ps)
Bak (Ps)
TRADD (Ps)
MST1 (Ps)
DAPK1 (Ps)
CASP1 (Ps)
ZIPK (Ps)
CASP12 (Ps)
FAK (Y576)
FAK (Y577)
p73 (Ps)
ZIPK (Ps)
XIAP (Ps)
CASP4 (Ps)
CASP9 (Ps)
Bad (S75)
AIF (Ps)
p53 (Ps)
p53 (S392)
RIPK1 (Ps)
DRAK1 (Ps)
FAK (Y861)
CaMKK (CaMKK2) (Ps)
Bcl2 (Ps)
Shc1 (Y349+Y350)
FAK (S732)
CaMK1d (Ps)
FasL (Ps)
ASK1 (MAP3K5) (Ps)
FAK (S910)
p53 (S392)
PTEN (S370)
ATM (S1981)
SODD (Ps)
CASP3 (Ps)
FAS (Ps)
MST1 (Ps)
FAK (Y397)
CASP8 (Ps)
CASP5 (Ps)
FAK (Ps)
NME7 (Ps)
Bcl-xS/L (Ps)
MST1 (Ps)
MST2 (Ps)
Trail (Ps)
NME6 (Ps)
PTEN (S380+S382+S385)
DFF45/35 (Ps)
Bid (Ps)
Nip1 (Ps)
SLK (Ps)
FAK (Y577)
FAK (S843)
CASP7 (Ps)
Bax (Ps)
FAK (Y576)
c-IAP1 (Ps)
PTEN (Ps)
CASP6 (Ps)
DAXX (Ps)

Figure S8

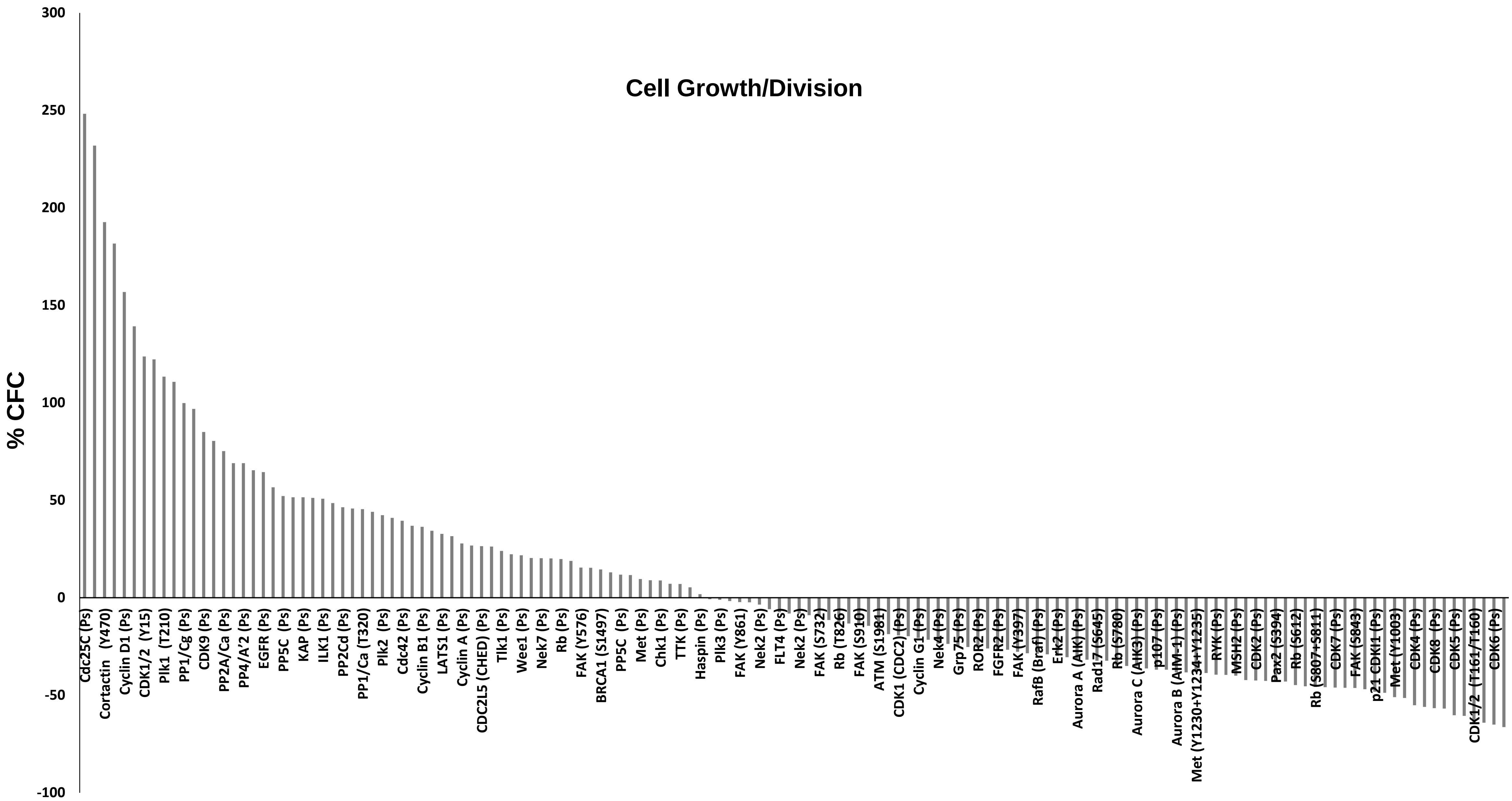

Cell Growth/Division
% CFC
300
250
200
150
100
50
0
-50
-100
Cdc25C (Ps)
Cortactin (Y470)
Cyclin D1 (Ps)
CDK1/2 (Y15)
Plk1 (T210)
PP1/Cg (Ps)
CDK9 (Ps)
PP2A/Ca (Ps)
PP4/A'2 (Ps)
EGFR (Ps)
PP5C (Ps)
KAP (Ps)
ILK1 (Ps)
PP2Cd (Ps)
PP1/Ca (T320)
Plk2 (Ps)
Cdc42 (Ps)
Cyclin B1 (Ps)
LATS1 (Ps)
Cyclin A (Ps)
CDC2L5 (CHED) (Ps)
Tlk1 (Ps)
Wee1 (Ps)
Nek7 (Ps)
Rb (Ps)
FAK (Y576)
BRCA1 (S1497)
PP5C (Ps)
Met (Ps)
Chk1 (Ps)
TTK (Ps)
Haspin (Ps)
Plk3 (Ps)
FAK (Y861)
Nek2 (Ps)
FLT4 (Ps)
Nek2 (Ps)
FAK (S732)
Rb (T826)
FAK (S910)
ATM (S1981)
CDK1 (CDC2) (Ps)
Cyclin G1 (Ps)
Nek4 (Ps)
Grp75 (Ps)
ROR2 (Ps)
FGFR2 (Ps)
FAK (Y397)
RafB (Braf) (Ps)
Erk2 (Ps)
Aurora A (AIK) (Ps)
Rad17 (S645)
Rb (S780)
Aurora C (AIK3) (Ps)
p107 (Ps)
Aurora B (AIM-1) (Ps)
Met (Y1230+Y1234+Y1235)
RYK (Ps)
MSH2 (Ps)
CDK2 (Ps)
Pax2 (S394)
Rb (S612)
Rb (S807+S811)
CDK7 (Ps)
FAK (S843)
p21 CDKI1 (Ps)
Met (Y1003)
CDK4 (Ps)
CDK8 (Ps)
CDK5 (Ps)
CDK1/2 (T161/T160)
CDK6 (Ps)

Figure S9

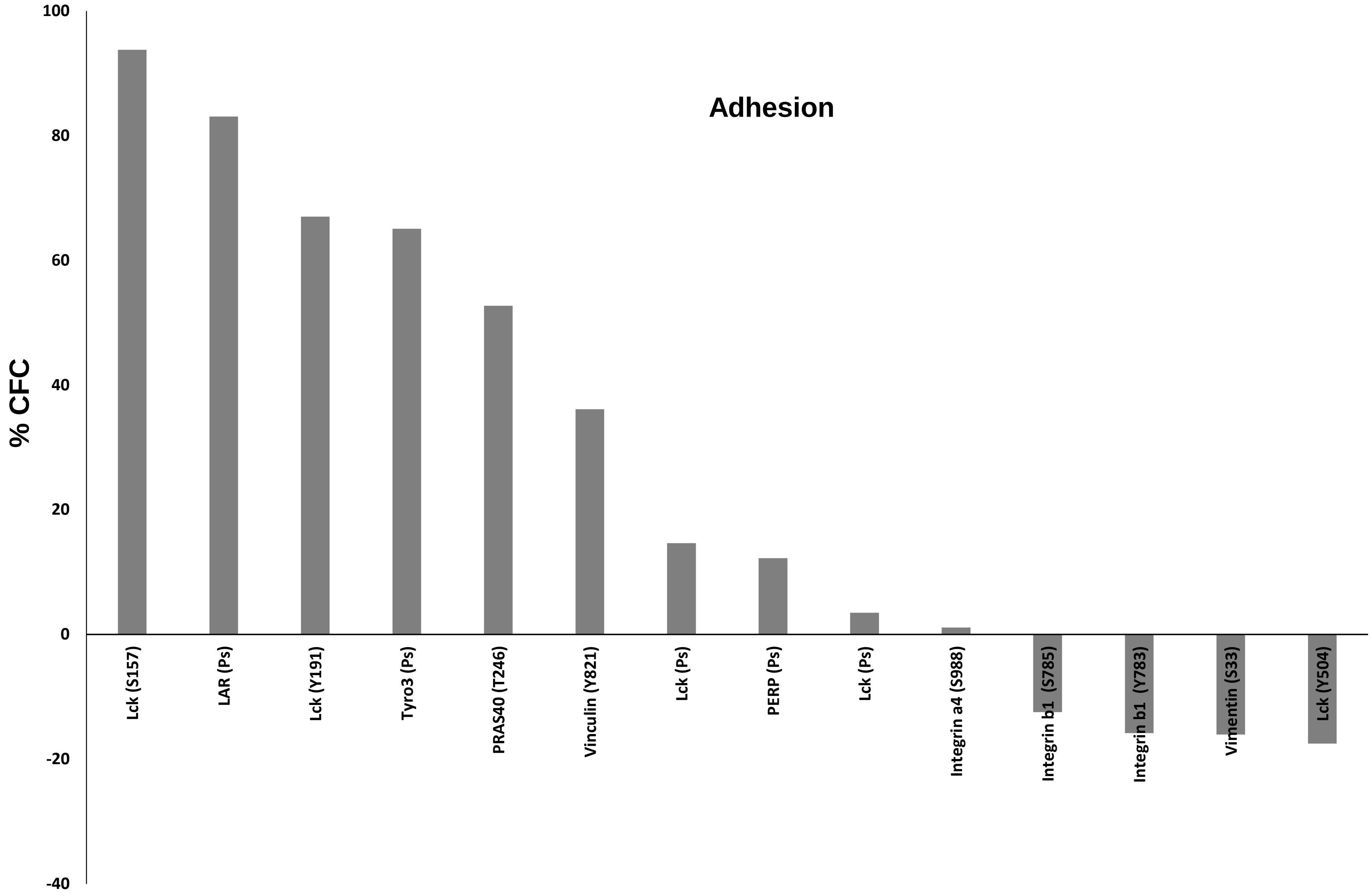

Adhesion
% CFC
100
80
60
40
20
0
-20
-40
Lck (S157)
LAR (Ps)
Lck (Y191)
Tyro3 (Ps)
PRAS40 (T246)
Vinculin (Y821)
Lck (Ps)
PERP (Ps)
Lck (Ps)
Integrin a4 (S988)
Integrin b1 (S785)
Integrin b1 (Y783)
Vimentin (S33)
Lck (Y504)

Figure S10

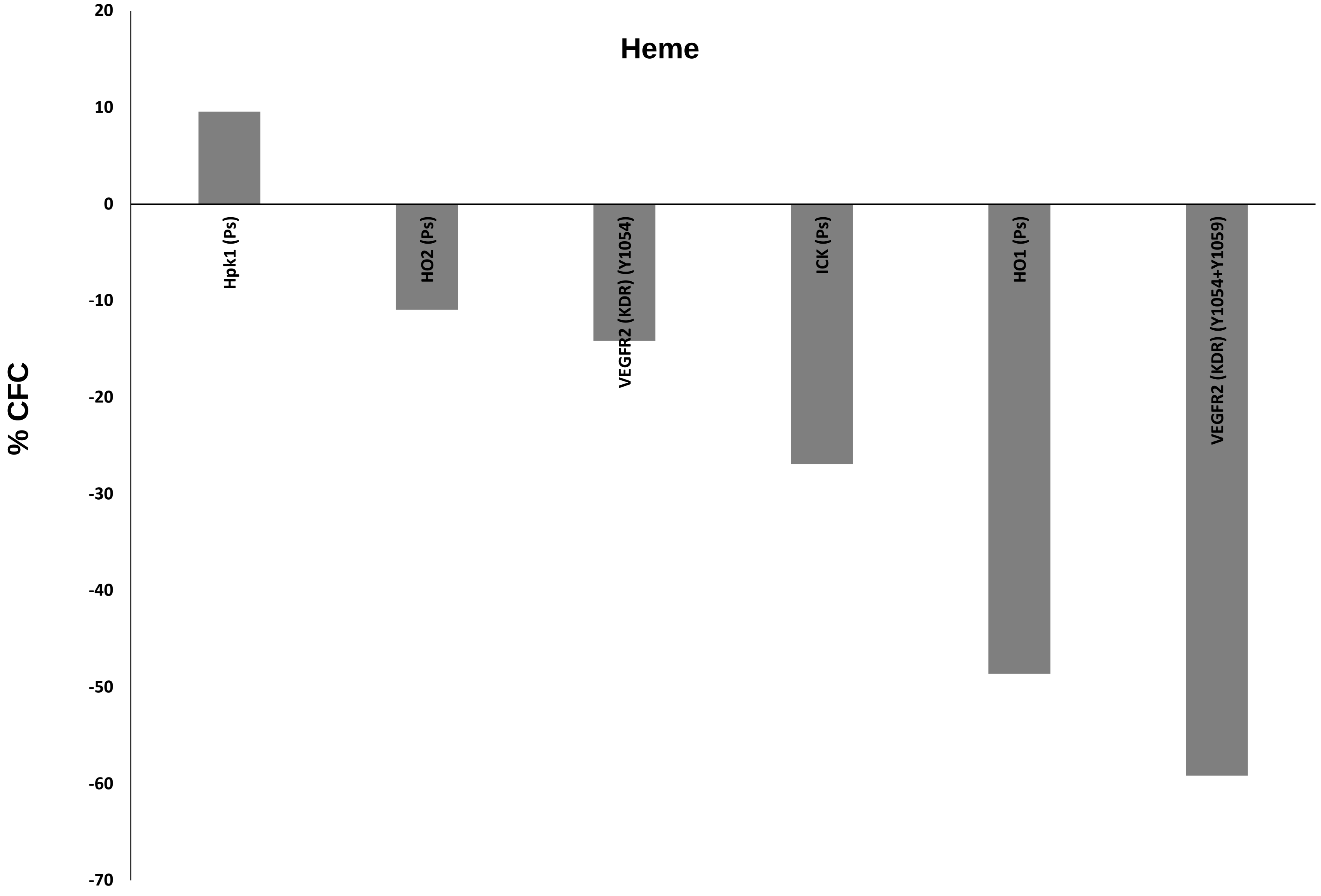

Heme
% CFC
20
10
0
-10
-20
-30
-40
-50
-60
-70
Hpk1 (Ps)
HO2 (Ps)
VEGFR2 (KDR) (Y1054)
ICK (Ps)
HO1 (Ps)
VEGFR2 (KDR) (Y1054+Y1059)

Figure S11

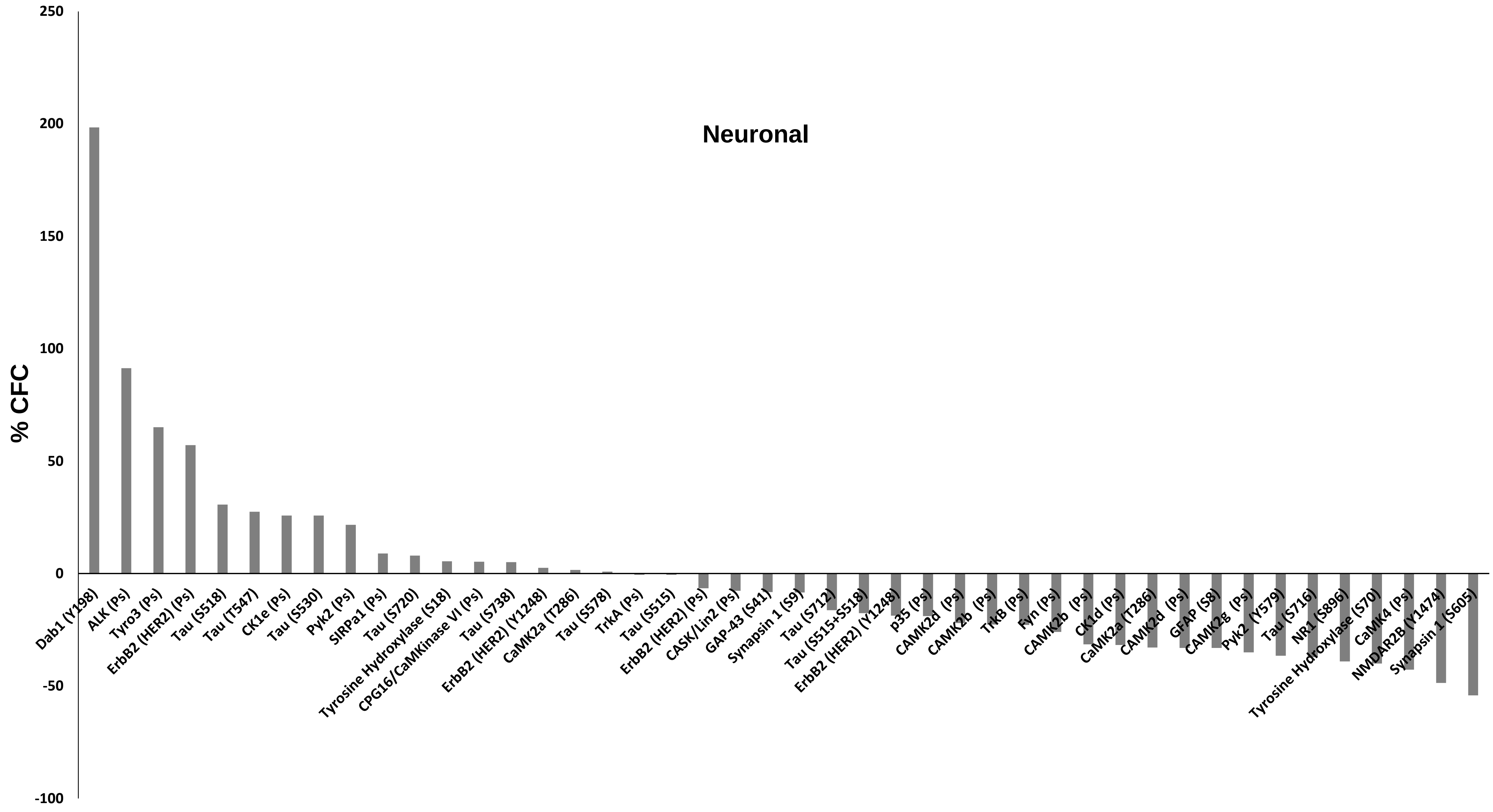
Neuronal
% CFC
250
200
150
100
50
0
-50
-100
Dab1 (Y198)
ALK (Ps)
Tyro3 (Ps)
ErbB2 (HER2) (Ps)
Tau (S518)
Tau (T547)
CK1e (Ps)
Tau (S530)
Pyk2 (Ps)
SIRPa1 (Ps)
Tau (S720)
Tyrosine Hydroxylase (S18)
CPG16/CaMKinase VI (Ps)
Tau (S738)
ErbB2 (HER2) (Y1248)
CaMK2a (T286)
Tau (S578)
TrkA (Ps)
Tau (S515)
ErbB2 (HER2) (Ps)
CASK/Lin2 (Ps)
GAP-43 (S41)
Synapsin 1 (S9)
Tau (S712)
Tau (S515+S518)
ErbB2 (HER2) (Y1248)
p35 (Ps)
CAMK2d (Ps)
CAMK2b (Ps)
TrkB (Ps)
Fyn (Ps)
CAMK2b (Ps)
CK1d (Ps)
CaMK2a (T286)
CAMK2d (Ps)
GFAP (S8)
CAMK2g (Ps)
Pyk2 (Y579)
Tau (S716)
NR1 (S896)
Tyrosine Hydroxylase (S70)
CaMK4 (Ps)
NMDAR2B (Y1474)
Synapsin 1 (S605)

Figure S12

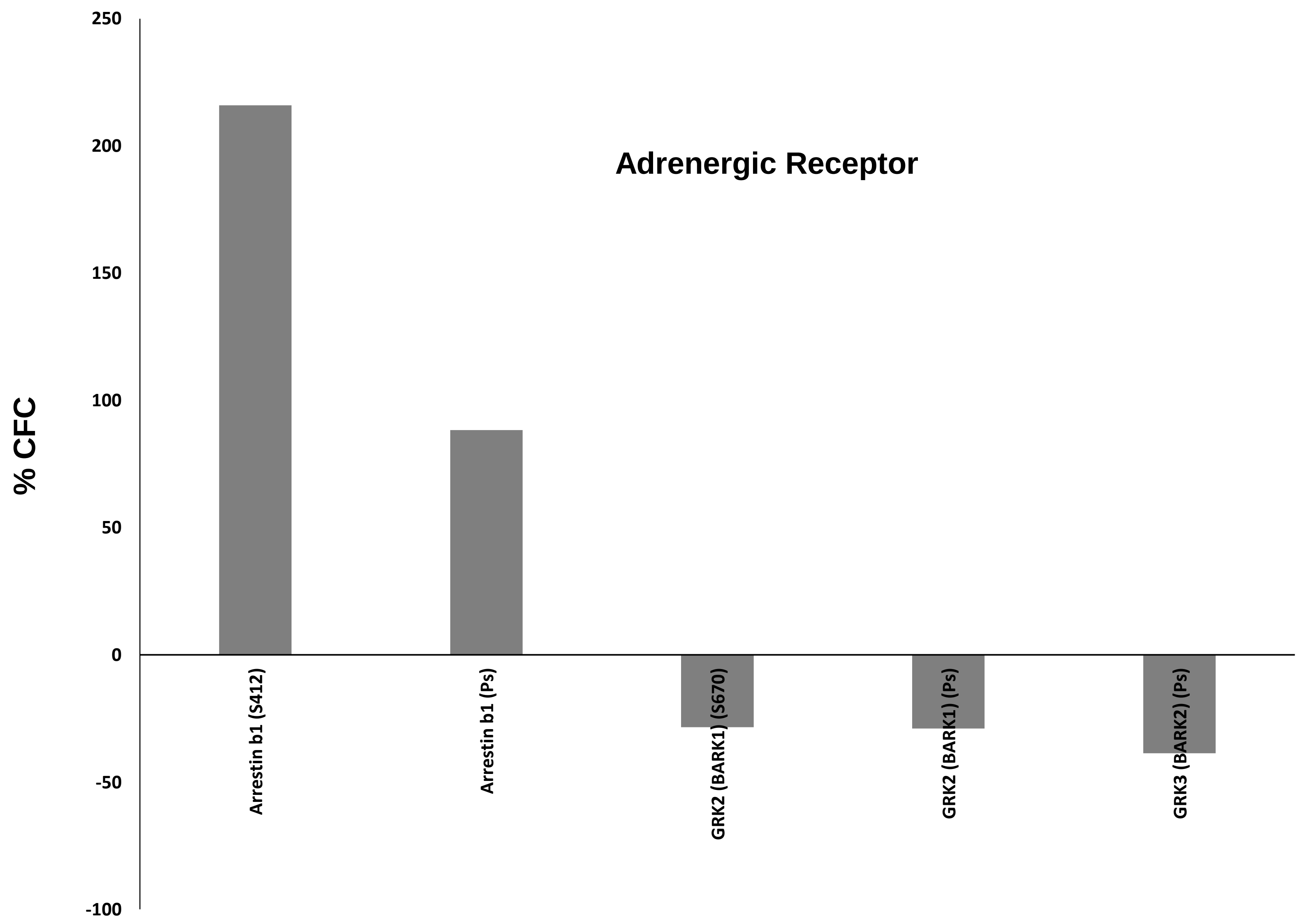
Adrenergic Receptor
% CFC
250
200
150
100
50
0
-50
-100
Arrestin b1 (S412)
Arrestin b1 (Ps)
GRK2 (BARK1) (S670)
GRK2 (BARK1) (Ps)
GRK3 (BARK2) (Ps)

Figure S13

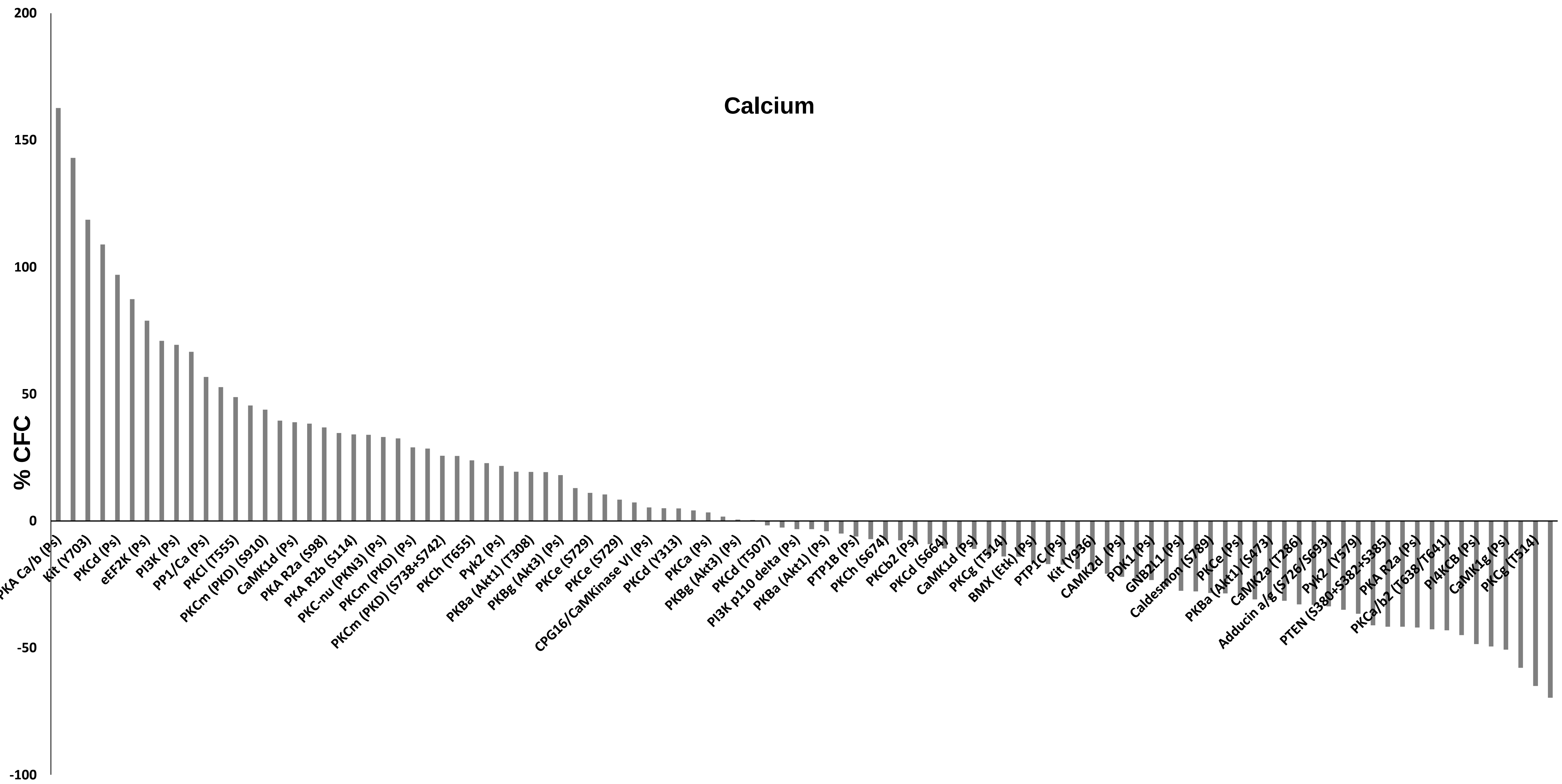

Calcium
% CFC
200
150
100
50
0
-50
-100
PKA Ca/b (Ps)
Kit (Y703)
PKCd (Ps)
eEF2K (Ps)
PI3K (Ps)
PP1/Ca (Ps)
PKCl (T555)
PKCm (PKD) (S910)
CaMK1d (Ps)
PKA R2a (S98)
PKA R2b (S114)
PKC-nu (PKN3) (Ps)
PKCm (PKD) (Ps)
PKCm (PKD) (S738+S742)
PKCh (T655)
Pyk2 (Ps)
PKBa (Akt1) (T308)
PKBg (Akt3) (Ps)
PKCe (S729)
PKCe (S729)
CPG16/CaMKinase VI (Ps)
PKCd (Y313)
PKCa (Ps)
PKBg (Akt3) (Ps)
PKCd (T507)
PI3K p110 delta (Ps)
PKBa (Akt1) (Ps)
PTP1B (Ps)
PKCh (S674)
PKCb2 (Ps)
PKCd (S664)
CaMK1d (Ps)
PKCg (T514)
BMX (Etk) (Ps)
PTP1C (Ps)
Kit (Y936)
CAMK2d (Ps)
PDK1 (Ps)
GNB2L1 (Ps)
Caldesmon (S789)
PKCe (Ps)
PKBa (Akt1) (S473)
CaMK2a (T286)
Adducin a/g (S726/S693)
Pyk2 (Y579)
PTEN (S380+S382+S385)
PKA R2a (Ps)
PKCa/b2 (T638/T641)
PI4KCB (Ps)
CaMK1g (Ps)
PKCg (T514)

Figure S14

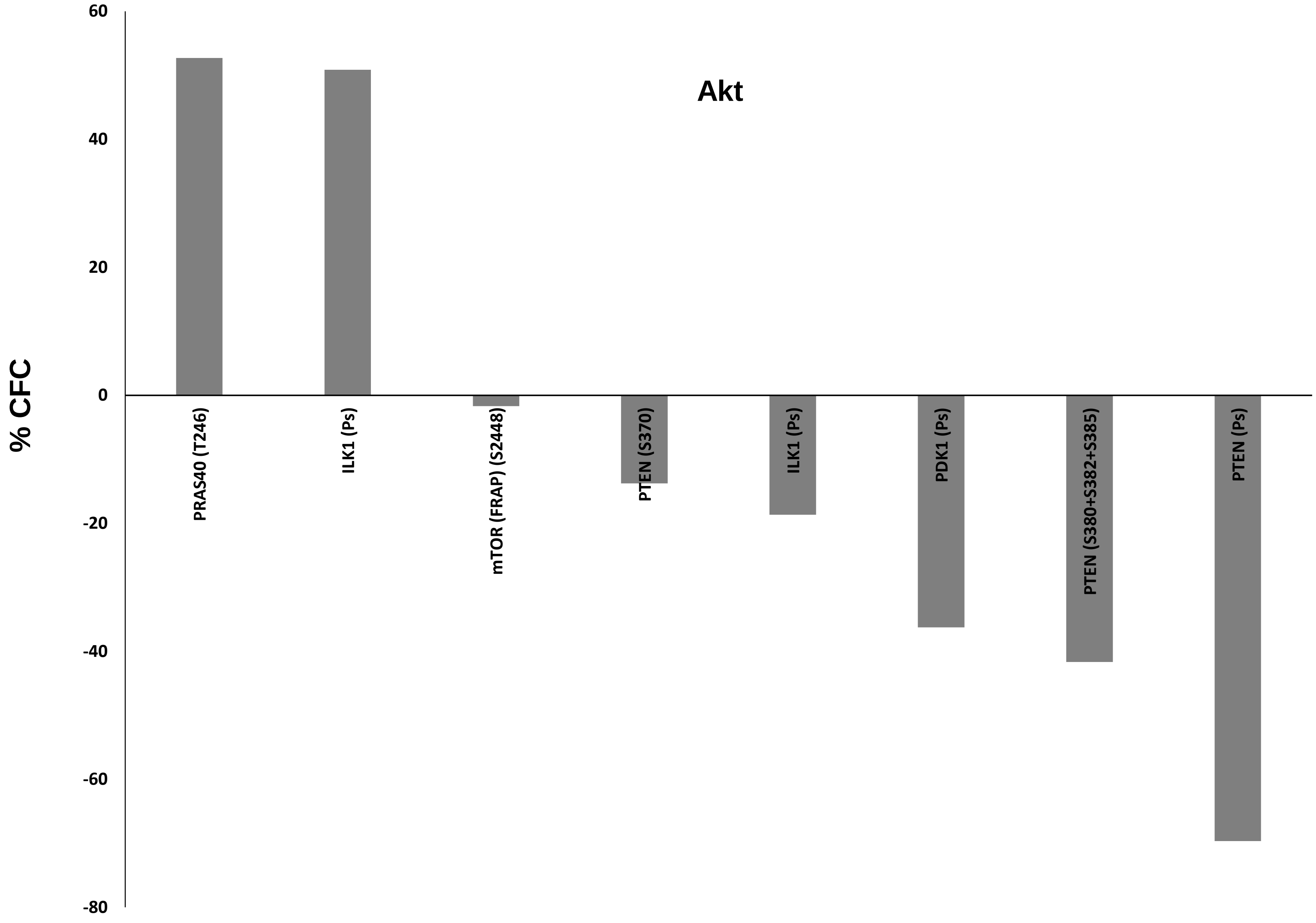
Akt
% CFC
60
40
20
0
-20
-40
-60
-80
PRAS40 (T246)
ILK1 (Ps)
mTOR (FRAP) (S2448)
PTEN (S370)
ILK1 (Ps)
PDK1 (Ps)
PTEN (S380+S382+S385)
PTEN (Ps)

Figure S15

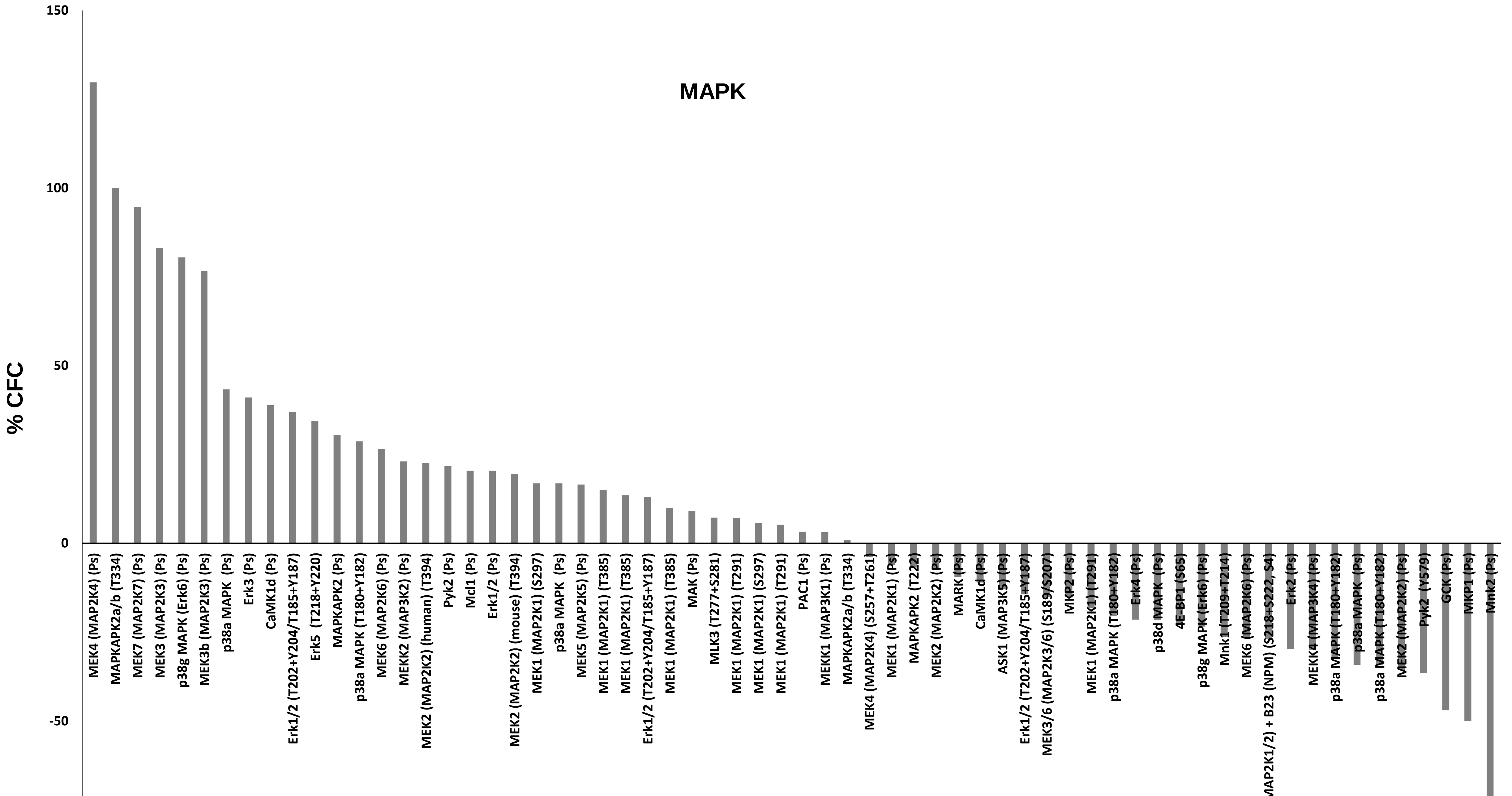
MAPK
% CFC
150
100
50
0
-50
MEK4 (MAP2K4) (Ps)
MAPKAPK2a/b (T334)
MEK7 (MAP2K7) (Ps)
MEK3 (MAP2K3) (Ps)
p38g MAPK (Erk6) (Ps)
MEK3b (MAP2K3) (Ps)
p38a MAPK (Ps)
Erk3 (Ps)
CaMK1d (Ps)
Erk1/2 (T202+Y204/T185+Y187)
Erk5 (T218+Y220)
MAPKAPK2 (Ps)
p38a MAPK (T180+Y182)
MEK6 (MAP2K6) (Ps)
MEKK2 (MAP3K2) (Ps)
MEK2 (MAP2K2) (human) (T394)
Pyk2 (Ps)
Mcl1 (Ps)
Erk1/2 (Ps)
MEK2 (MAP2K2) (mouse) (T394)
MEK1 (MAP2K1) (S297)
p38a MAPK (Ps)
MEK5 (MAP2K5) (Ps)
MEK1 (MAP2K1) (T385)
MEK1 (MAP2K1) (T385)
Erk1/2 (T202+Y204/T185+Y187)
MEK1 (MAP2K1) (T385)
MAK (Ps)
MLK3 (T277+S281)
MEK1 (MAP2K1) (T291)
MEK1 (MAP2K1) (S297)
MEK1 (MAP2K1) (T291)
PAC1 (Ps)
MEKK1 (MAP3K1) (Ps)
MAPKAPK2a/b (T334)
MEK4 (MAP2K4) (S257+T261)
MEK1 (MAP2K1) (Ps)
MAPKAPK2 (T222)
MEK2 (MAP2K2) (Ps)
MARK (Ps)
CaMK1d (Ps)
ASK1 (MAP3K5) (Ps)
Erk1/2 (T202+Y204/T185+Y187)
MEK3/6 (MAP2K3/6) (S189/S207)
MKP2 (Ps)
MEK1 (MAP2K1) (T291)
p38a MAPK (T180+Y182)
Erk4 (Ps)
p38d MAPK (Ps)
4E-BP1 (S65)
p38g MAPK (Erk6) (Ps)
Mnk1 (T209+T214)
MEK6 (MAP2K6) (Ps)
MAP2K1/2) + B23 (NPM) (S218+S222, S4)
Erk2 (Ps)
MEKK4 (MAP3K4) (Ps)
p38a MAPK (T180+Y182)
p38a MAPK (Ps)
p38a MAPK (T180+Y182)
MEK2 (MAP2K2) (Ps)
Pyk2 (Y579)
GCK (Ps)
MKP1 (Ps)
Mnk2 (Ps)

Figure S16

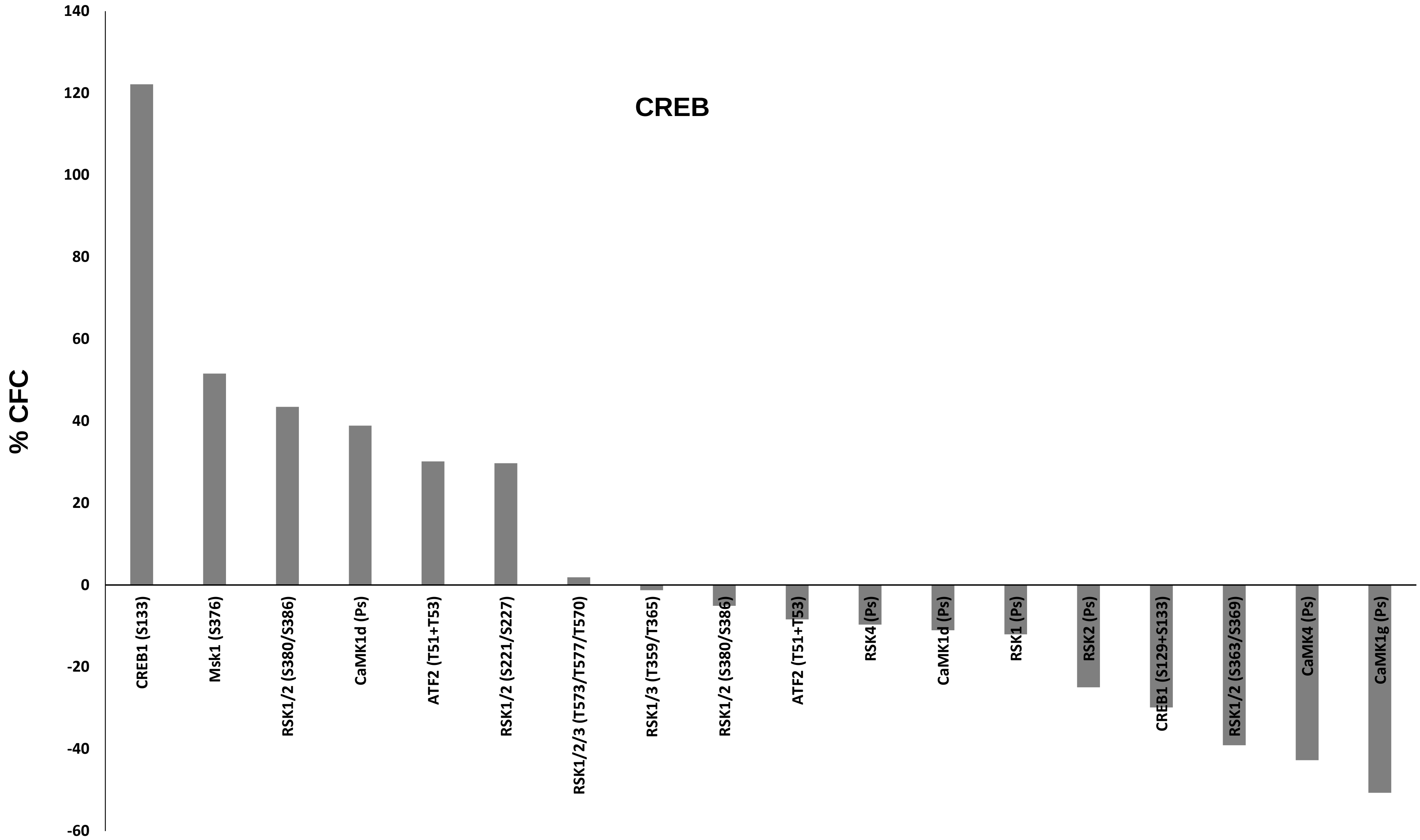

CREB
% CFC
140
120
100
80
60
40
20
0
-20
-40
-60
CREB1 (S133)
Msk1 (S376)
RSK1/2 (S380/S386)
CaMK1d (Ps)
ATF2 (T51+T53)
RSK1/2 (S221/S227)
RSK1/2/3 (T573/T577/T570)
RSK1/3 (T359/T365)
RSK1/2 (S380/S386)
ATF2 (T51+T53)
RSK4 (Ps)
CaMK1d (Ps)
RSK1 (Ps)
RSK2 (Ps)
CREB1 (S129+S133)
RSK1/2 (S363/S369)
CaMK4 (Ps)
CaMK1g (Ps)

Figure S17

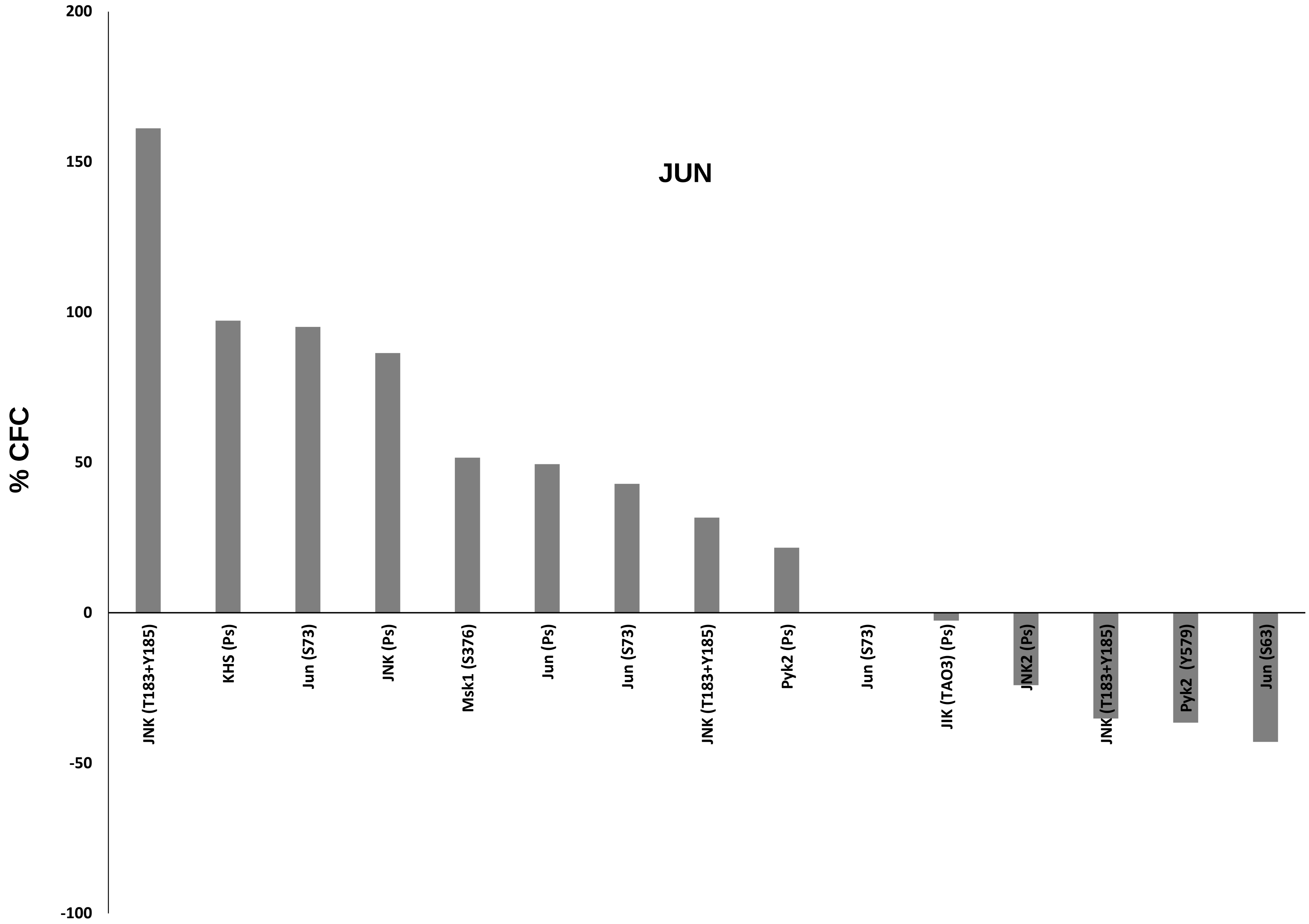
JUN
% CFC
200
150
100
50
0
-50
-100
JNK (T183+Y185)
KHS (Ps)
Jun (S73)
JNK (Ps)
Msk1 (S376)
Jun (Ps)
Jun (S73)
JNK (T183+Y185)
Pyk2 (Ps)
Jun (S73)
JIK (TAO3) (Ps)
JNK2 (Ps)
JNK (T183+Y185)
Pyk2 (Y579)
Jun (S63)

Figure S18

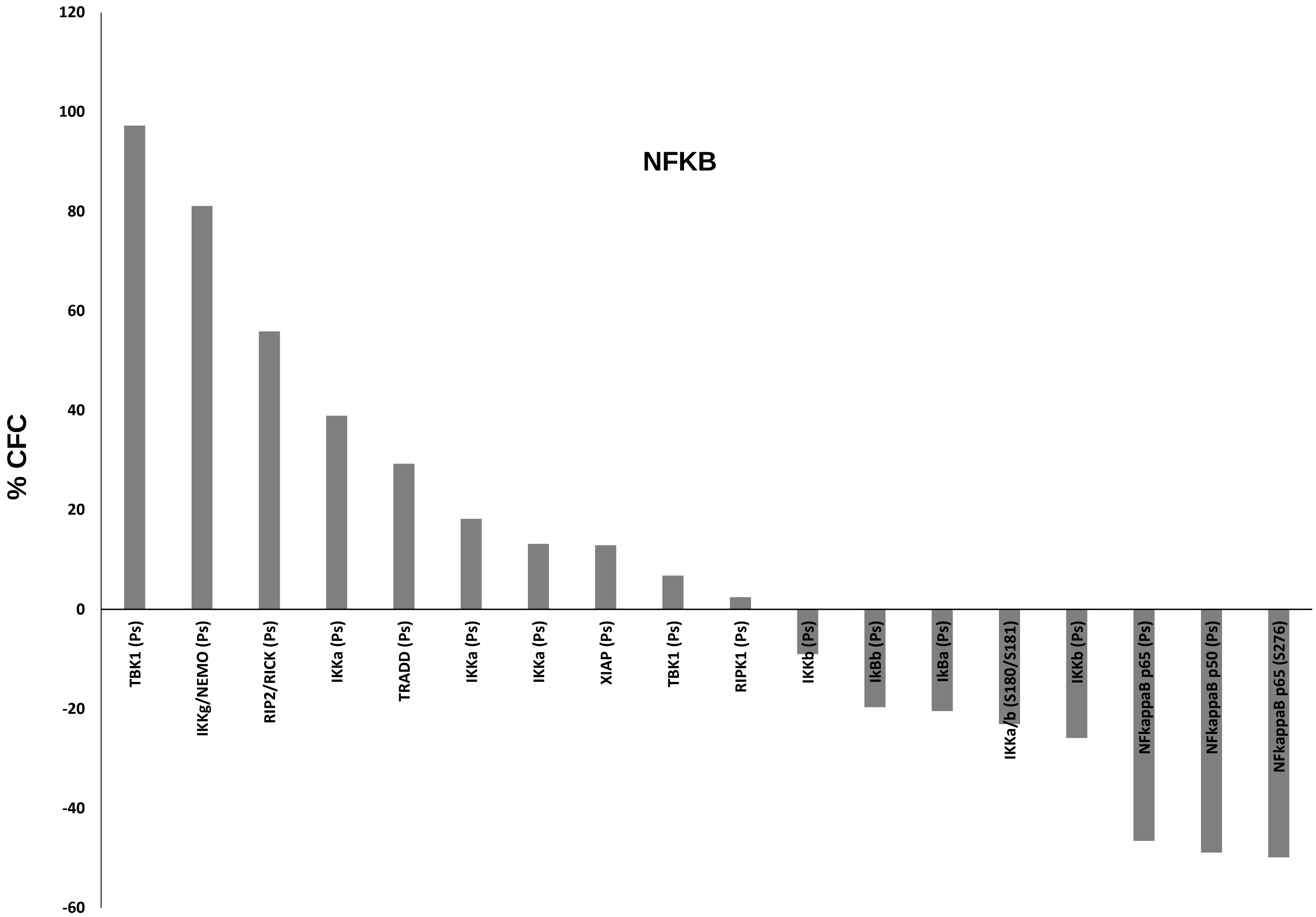
NFKB
% CFC
120
100
80
60
40
20
0
-20
-40
-60
TBK1 (Ps)
IKKg/NEMO (Ps)
RIP2/RICK (Ps)
IKKa (Ps)
TRADD (Ps)
IKKa (Ps)
IKKa (Ps)
XIAP (Ps)
TBK1 (Ps)
RIPK1 (Ps)
IKKb (Ps)
IkBb (Ps)
IkBa (Ps)
IKKa/b (S180/S181)
IKKb (Ps)
NFkappaB p65 (Ps)
NFkappaB p50 (Ps)
NFkappaB p65 (S276)

Figure S19

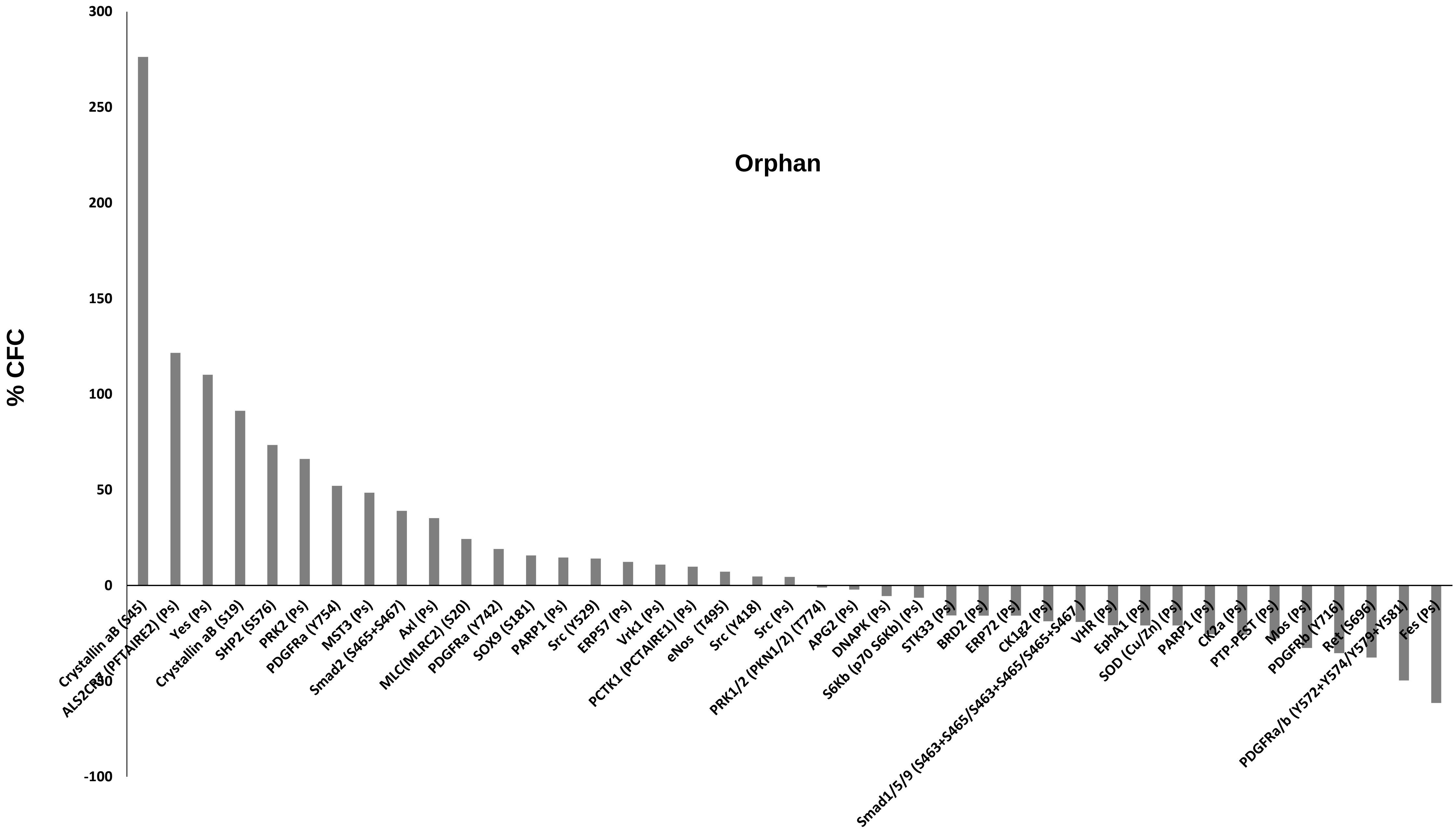

Orphan
% CFC
300
250
200
150
100
50
0
-100
Crystallin aB (S45)
ALS2CR7 (PFTAIRE2) (Ps)
Yes (Ps)
Crystallin aB (S19)
SHP2 (S576)
PRK2 (Ps)
PDGFRa (Y754)
MST3 (Ps)
Smad2 (S465+S467)
Axl (Ps)
MLC(MLRC2) (S20)
PDGFRa (Y742)
SOX9 (S181)
PARP1 (Ps)
Src (Y529)
ERP57 (Ps)
Vrk1 (Ps)
PCTK1 (PCTAIRE1) (Ps)
eNos (T495)
Src (Y418)
Src (Ps)
PRK1/2 (PKN1/2) (T774)
APG2 (Ps)
DNAPK (Ps)
S6Kb (p70 S6Kb) (Ps)
STK33 (Ps)
BRD2 (Ps)
ERP72 (Ps)
CK1g2 (Ps)
Smad1/5/9 (S463+S465/S463+S465/S465+S467)
VHR (Ps)
EphA1 (Ps)
SOD (Cu/Zn) (Ps)
PARP1 (Ps)
CK2a (Ps)
PTP-PEST (Ps)
Mos (Ps)
PDGFRb (Y716)
Ret (S696)
PDGFRa/b (Y572+Y574/Y579+Y581)
Fes (Ps)

Supplementary Table 1

Serial No.	Antibody Code	Target Protein Name	Phospho Site (Human)	Full Target Protein Name	Swiss-prot Link	% CFC
**Lipid Metabolism**						
1250	NK005	AMPKb	Pan-specific	5'-AMP-activated protein kinase subunit beta-1	Q9Y478	101.8
318	PK020	FAK	S722	Focal adhesion protein-tyrosine kinase	Q05397	51.2
26	PK002	AMPKa1/2	T174/T172	AMP-activated protein-serine kinase alpha 1/2	Q13131	38.2
116	NN011-NN125	CASP1	Pan-specific	Pro-caspase 1 (Interleukin-1 beta convertase) alpha/beta isoform	P29466	26.8
10	PN002	AcCoA carboxylase (ACC)	S80	Acetyl coenzyme A carboxylase	Q13085	18.1
330	PK018-1	FAK	Y576	Focal adhesion protein-tyrosine kinase	Q05397	15.5
328	PK019-2	FAK	Y577	Focal adhesion protein-tyrosine kinase	Q05397	15.3
78	PN014	BRCA1	S1497	Breast cancer type 1 susceptibility protein	P38398	14.5
336	PK023	FAK	Y861	Focal adhesion protein-tyrosine kinase	Q05397	-2.2
2	NN001	14-3-3 z	Pan-specific	14-3-3 protein zeta (cross-reacts with other isoforms)	P63104	-3.1
1092	NN101	SPHK2	Pan-specific	Sphingosine kinase 2	Q9NRA0	-4.3
320	PK021	FAK	S732	Focal adhesion protein-tyrosine kinase	Q05397	-9.3
324	PK024	FAK	S910	Focal adhesion protein-tyrosine kinase	Q05397	-13.5
962	NK150	PRKAB1	Pan-specific	5'-AMP-activated protein kinase (AMPK), beta-1 regulatory subunit	Q9Y478	-15.7
120	NN013	CASP3	Pan-specific	Pro-caspase 3 (apopain, cysteine protease CPP32)	P42574	-20.9
966	PN104	Progesterone Receptor (PR)	S294	Progesterone receptor	Q13523	-27.7
326	PK017	FAK	Y397	Focal adhesion protein-tyrosine kinase	Q05397	-27.7
316	NK060	FAK	Pan-specific	Focal adhesion protein-tyrosine kinase	Q05397	-32.3
1090	NN100	SPHK1	Pan-specific	Sphingosine kinase 1	Q9NYA1	-39.8
334	PK019-1	FAK	Y577	Focal adhesion protein-tyrosine kinase	Q05397	-45.4
322	PK022	FAK	S843	Focal adhesion protein-tyrosine kinase	Q05397	-46.2
128	NN017	CASP7	Pan-specific	Pro-caspase 7 (ICE-like apoptotic protease 3 (ICE-LAP3), Mch3)	P55210	-47.6
332	PK018-2	FAK	Y576	Focal adhesion protein-tyrosine kinase	Q05397	-56.8

**Glucose Metabolism**						
954	NP022	PP6C	Pan-specific	Protein-serine phosphatase 6 - catalytic subunit (PPVC)	O00743	181.6
486	PK033	IR/IGF1R (INSR)	Y1189/Y1190	Insulin receptor/Insulin-like growth factor 1 receptor	P06213	179.1
934	NP010	PP1/Cb	Pan-specific	Protein-serine phosphatase 1 - catalytic subunit - beta isoform	P62140	139.2
938	NP012	PP2A/Aa/b	Pan-specific	Protein-serine phosphatase 2A - A regulatory subunit - alpha and beta isoforms	P30153	122.2
944	NP016-NP031	PP2Ca/b	Pan-specific	Protein-serine phosphatase 2C - catalytic subunit - alpha/beta	P35813	110.7
1274	NK079	IR	Pan-specific	Insulin receptor beta chain	P06213	110.5
484	PK032	IR (INSR)	Y999	Insulin receptor	P06213	102.0
936	NP011	PP1/Cg	Pan-specific	Protein-serine phosphatase 1 - catalytic subunit - gamma isoform	P36873	99.8
940	NP013-NP014	PP2A/Ca	Pan-specific	Protein-serine phosphatase 2A - catalytic subunit alpha isoform	P67775	75.2
1276	NN089	PI3K	Pan-specific	Phosphatidylinositol 3-kinase regulatory subunit alpha	P27986	69.3
950	NP020	PP4C	Pan-specific	Protein-serine phosphatase X - catalytic subunit (PPX/C)	P60510	69.0
948	NP019	PP4/A’2	Pan-specific	Protein-serine phosphatase 4 - regulatory subunit (PPX/A'2)	Q8TF05	69.0
930	NP009	PP1/Ca	Pan-specific	Protein-serine phosphatase 1 - catalytic subunit - alpha isoform	P62136	56.7
952	NP021	PP5C	Pan-specific	Protein-serine phosphatase 5 - catalytic subunit (PPT)	P53041	52.2
946	NP018	PP2Cd	Pan-specific	Protein-serine phosphatase 2C - catalytic subunit - delta isoform	O15297	46.5
932	PP001	PP1/Ca	T320	Protein-serine phosphatase 1 - catalytic subunit - alpha isoform	P62136	45.4
498	PN046	IRS1	Y1179	Insulin receptor substrate 1	P35568	36.9
942	NP015	PP2B/Aa	Pan-specific	Protein-serine phosphatase 2B - catalytic subunit - alpha isoform	Q08209	26.7
1238	NN037	Dok1	Pan-specific	Docking protein 1	Q99704	24.7
1072	NN094	SIRPa1	Pan-specific	Signal regulatory protein substrate of PTP1D phosphatase (SHPS1)	P78324	8.9
912	NN115	PKM2	Pan-specific	Pyruvate kinase, isozymes M1/M2	P14618	7.1
1054	NK168-	S6Ka/b (p70	Pan-specific	p70 ribosomal protein-serine	P23443	6.3

	NK169	S6Ka)		S6 kinase alpha/beta		
1058	PK105	S6Ka (p70/p85 S6Ka)	T421+S424	p70/p85 ribosomal protein-serine S6 kinase alpha	P23443	6.2
788	PN061	PED15 (PEA15)	S116	Phosphoprotein-enriched in diabetes/astrocytes 15	Q15121	2.5
1060	PK104	S6Ka (p70/p85 S6Ka)	T229	p70/p85 ribosomal protein-serine S6 kinase alpha	P23443	-3.5
786	NK153	PyDK2 (PDHK2)	Pan-specific	Pyruvate dehydrogenase kinase isoform 2	Q15119	-4.4
246	NN036	DGKz	Pan-specific	Diacylglycerol kinase zeta	Q13574	-4.8
388	PK028-PK029-2	GSK3a/b	Y279/ Y216	Glycogen synthase-serine kinase 3 beta	P49841	-9.1
456	NK074	IGF1R	Pan-specific	Insulin-like growth factor receptor protein-tyrosine kinase	P08069	-13.0
386	PK028-PK029-1	GSK3a/b	Y279/ Y216	Glycogen synthase-serine kinase 3 beta	P49841	-21.4
500	PN045	IRS1	Y612	Insulin receptor substrate 1	P35568	-23.8
1056	PK106	S6Ka (p70/p85 S6Ka)	T389	p70/p85 ribosomal protein-serine S6 kinase alpha	P23443	-25.9
768	NN113	PCK2	Pan-specific	Phosphoenolpyruvate carboxykinase	Q16822	-34.1
986	NK153	PyDK2 (PDHK2)	Pan-specific	Pyruvate dehydrogenase kinase isoform 2	Q14289	-35.6
382	NK069-NK070	GSK3a/b	Pan-specific	Glycogen synthase-serine kinase 3 beta	P49841	-61.0
384	PK026-PK027-1	GSK3a/b	S21/S9	Glycogen synthase-serine kinase 3 beta	P49841	-62.9
			**Inflammation**			
496	NK083-2	IRAK4	Ps	Interleukin 1 receptor-associated kinase 4	Q9NWZ3	121.5
506	NK085	JAK2	Ps	Janus protein-tyrosine kinase 2	O60674	114.3
1162	NN109-2	TBK1	Ps	Tank-binding protein 1	Q9UHD2	97.3
250	PN027	Dok2	Y142	Docking protein 2 (mouse)	O60496	96.8
554	PK039	Lck	S157	Lymphocyte-specific protein-tyrosine kinase	P06239	93.8
504	NK084-2	JAK1	Ps	Janus protein-tyrosine kinase 1	P23458	82.8
310	NK059-1	p38g MAPK (Erk6)	Ps	Mitogen-activated protein-serine kinase p38 gamma (MAPK12)	P53778	80.4
556	PK040	Lck	Y191	Lymphocyte-specific protein-tyrosine kinase	P06239	67.0
222	NK044	Csk	Ps	C-terminus of Src tyrosine kinase	P41240	62.9
1066	NK170	SGK3	Ps	Serum/glucocorticoid regulated kinase 3	Q96BR1	60.0
1180	NK181	Tyk2	Ps	Protein-tyrosine kinase 2 (Jak-related)	P29597	59.5

564	NK095	Lyn	Ps	Yes-related protein-tyrosine kinase	P07948	56.3
1212	PK113	ZAP70	Y315+ Y319	Zeta-chain (TCR) associated protein-tyrosine kinase, 70 kDa	P43403	48.9
492	NK082	IRAK3	Ps	Interleukin 1 receptor-associated kinase 3	Q9Y616	46.4
1138	NK175-2	TAK1	Ps	TGF-beta-activated protein-serine kinase 1	O43318	45.7
1210	PK112	ZAP70	Y292	Zeta-chain (TCR) associated protein-tyrosine kinase, 70 kDa	P43403	39.8
566	PK043	Lyn	Y507	Yes-related protein-tyrosine kinase	P07948	34.5
116	NN011-NN125	CASP1	Ps	Pro-caspase 1 (Interleukin-1 beta convertase) alpha/beta isoform	P29466	26.8
198	NK037	CK1e	Ps	Casein protein-serine kinase 1 epsilon	P49674	25.8
488	NK080	IRAK1	Ps	Interleukin 1 receptor-associated kinase 1 (Pelle-like protein kinase)	P51617	23.3
1208	NK187	ZAP70	Ps	Zeta-chain (TCR) associated protein-tyrosine kinase, 70 kDa	P43403	23.2
212	NN027	COX2	Ps	Cyclo-oxygenase 2 (prostaglandin G/H synthase 2 precursor)	P35354	19.2
1248	NN096	Smad2/3	Ps	SMA- and mothers against decapentaplegic homolog 2/3	Q15796	18.5
1136	NK175-1	TAK1	Ps	TGF-beta-activated protein-serine kinase 1	O43318	18.0
552	NK092-2	Lck	Ps	Lymphocyte-specific protein-tyrosine kinase	P06239	14.6
562	NK094	LOK	Ps	Lymphocyte-oriented protein-serine kinase	O94804	13.7
146	NP001	CD45	Ps	Leukocyte common antigen CD45 receptor-tyrosine phosphatase (LCA, T200)	P08575	12.2
1072	NN094	SIRPa1	Ps	Signal regulatory protein substrate of PTP1D phosphatase (SHPS1)	P78324	8.9
1160	NN109-1	TBK1	Ps	Tank-binding protein 1	Q9UHD2	6.8
1278	NK161	RONa	Ps	Macrophage-stimulating protein receptor alpha chain	Q04912	6.4
1130	NK174	Syk	Ps	Spleen protein-tyrosine kinase	P43405	3.9
550	NK092-1	Lck	Ps	Lymphocyte-specific protein-tyrosine kinase	P06239	3.5
494	NK083-1	IRAK4	Ps	Interleukin 1 receptor-associated kinase 4	Q9NWZ3	2.6
1102	PN078	STAT1	S727	Signal transducer and activator of transcription 1	P42224	1.9

1030	PK116	mTOR (FRAP)	S2448	Mammalian target of rapamycin (FRAP)	P42345	-1.7
210	NK042	COT	Ps	Osaka thyroid oncogene protein-serine kinase (Tpl2)	P41279	-2.3
508	PK034	JAK2	Y1007/Y1008	Janus protein-tyrosine kinase 2	O60674	-3.1
1214	PK109	ZAP70/Syk	Y319/Y352	Zeta-chain (TCR) associated protein-tyrosine kinase, 70 kDa/Spleen protein-tyrosine kinase	P43403	-3.6
1082	NN097	SOCS4	Ps	Suppressor of cytokine signalling 4 (SOCS7)	Q8WXH5	-4.7
510	NK086	JAK3	Ps	Janus protein-tyrosine kinase 3	P52333	-5.0
560	PK042	LIMK1/2	Y507+T508/Y504+T505	LIM domain kinase 1/2	P53667	-5.2
502	NK084-1	JAK1	Ps	Janus protein-tyrosine kinase 1	P23458	-8.1
1104	PN082	STAT3	Y705	Signal transducer and activator of transcription 3	P40763	-8.5
28	NK006	ANKRD3	Ps	Ankyrin repeat domain protein-serine kinase 3 (RIPK4, DIK)	P57078	-11.2
1118	NN105	STAT5A	Ps	Signal transducer and activator of transcription 5A	P42229	-13.2
490	NK081	IRAK2	Ps	Interleukin 1 receptor-associated kinase 2	O43187	-14.8
1120	PN083	STAT5A	Y694	Signal transducer and activator of transcription 5A	P42229	-17.2
1086	NN099	SODD	Ps	Silencer of death domains (Bcl2 associated athanogene 4 (BAG4))	O95429	-17.4
558	PK041	Lck	Y504	Lymphocyte-specific protein-tyrosine kinase	P06239	-17.5
1106	PN079	STAT1	Y701	Signal transducer and activator of transcription 1	P42224	-17.8
1116	NN117	STAT4	Ps	Signal transducer and activator of transcription 4 (acute phase response factor)	Q14765	-18.8
1122	NN106	STAT5B	Ps	Signal transducer and activator of transcription 5B	P51692	-19.8
1100	NN102	STAT1a/b	Ps	Signal transducer and activator of transcription 1 alpha/beta	P42224	-23.0
1112	NN104	STAT3	Ps	Signal transducer and activator of transcription 3 (acute phase response factor)	P40763	-23.1
1108	NN103	STAT2	Ps	Signal transducer and activator of transcription 2	P52630	-24.3
1114	PN081	STAT3	S727	Signal transducer and activator of transcription 3 (acute phase response factor)	P40763	-25.1
1110	PN080	STAT2	Y690	Signal transducer and activator of transcription 2	P52630	-31.2

1124	NN107	STAT6	Ps	Signal transducer and activator of transcription 6	P42226	-31.8
672	NK116	mTOR (FRAP)	Ps	Mammalian target of rapamycin (FRAP)	P42345	-45.9
			**Chaperone**			
30	NN004	APG1	Pan-specific	Hsp 70-related heat shock protein 1 (osmotic stress protein 94 (OSP94))	O95757	148.3
416	NN054	Hsc70	Pan-specific	Heat shock 70 kDa protein 8	P11142	22.8
424	PN040-1	Hsp27	S15	Heat shock 27 kDa protein beta 1 (HspB1)	P04792	21.2
444	NN061	Hsp90	Pan-specific	Heat shock 90 kDa protein alpha	P07900	18.8
434	NN057	Hsp40	Pan-specific	DnaJ homolog, subfamily B member 1	P25685	18.4
446	NN062	Hsp105	Pan-specific	Heat shock 105 kDa protein	Q92598	16.4
422	PN042-3	Hsp27	S82	Heat shock 27 kDa protein beta 1 (HspB1)	P04792	6.9
426	PN040-2	Hsp27	S15	Heat shock 27 kDa protein beta 1 (HspB1)	P04792	2.0
448	NN063	HspBP1	Pan-specific	Hsp70 binding protein 1	O95351	0.0
398	PN036	Histone H2A.X	S139	Histone H2A variant X	P16104	-1.9
406	PN038	Histone H3	S10	Histone H3.3	P84243	-3.4
442	NN060	Hsp70	Pan-specific	Heat shock 70 kDa protein 1	P08107	-4.5
402	PN101	Histone H3	T3	Histone H3.3	P84243	-7.3
1126	NN108	STI1	Pan-specific	Stress induced phosphoprotein 1 (Hsc70/Hsp90 organizing protein (Hop))	P31948	-9.4
380	NN049	Grp94	Pan-specific	Glucose regulated protein 94 (endoplasmin)	P14625	-10.1
404	PN100	Histone H3	T11	Histone H3.3	P84243	-12.3
396	PN035	Histone H1	phospho CDK1 sites	Histone H1 phosphorylated	Q02539	-13.1
392	NN050	hHR23B	Pan-specific	UV excison repair protein RAD23 homolog B	P54727	-14.8
408	PN039	Histone H3	S28	Histone H3.3	P84243	-23.5
400	PN037	Histone H2B	S14	Histone H2B	P33778	-24.4
376	NN047	Grp75	Pan-specific	Glucose regulated protein 75	P38646	-25.0
420	NN056	Hsp27	Pan-specific	Heat shock 27 kDa protein beta 1 (HspB1)	P04792	-29.2
378	NN048	Grp78	Pan-specific	Glucose regulated protein 78	P11021	-30.7
438	NN059-1	Hsp60	Pan-specific	Heat shock 60 kDa protein 1 (chaperonin, CPN60)	P10809	-31.3
430	PN042-1	Hsp27	S82	Heat shock 27 kDa protein beta 1 (HspB1)	P04792	-35.1
436	NN058	Hsp47	Pan-specific	Heat shock 47 kDa protein (collagen-binding protein 1, colligin 1)	P29043	-36.0

418	NN055	HSF4	Pan-specific	Heat shock transcription factor 4	Q9ULV5	-38.5
432	PN042-2	Hsp27	S82	Heat shock 27 kDa protein beta 1 (HspB1)	P04792	-43.4
394	NN051	Hip	Pan-specific	Hsp70/Hsc70 interacting protein (ST13)	P50502	-55.1
440	NN059-2	Hsp60	Pan-specific	Heat shock 60 kDa protein 1 (chaperonin, CPN60)	P10809	-58.4
428	PN041	Hsp27	S78	Heat shock 27 kDa protein beta 1 (HspB1)	P04792	-99.9
			**Transport**			
208	PN022	Cortactin	Y470	Cortactin (amplaxin) (mouse)	Q60598	192.7
144	PN018	Caveolin 2	S36	Caveolin 2	P51636	124.7
1244	NN026	Cofilin	Pan-specific	Cofilin 1	P23528	101.5
112	NN010	CAS	Pan-specific	Cellular apoptosis susceptibility protein (CSE1L)	P55060	93.4
1254	NN092	Rac1	Pan-specific	Ras-related C3 botulinum toxin substrate 1	P60953	88.7
1240	NN086	Paxillin	Pan-specific	Paxillin 1	P49023	85.8
140	PN016	Catenin b	S45	Catenin (cadherin-associated protein) beta 1	P35222	84.9
1270	NK160	ROKb (ROCK1)	Pan-specific	Rho-associated protein kinase 1	Q13464	80.3
12	NK002	ACK1	Pan-specific	Activated p21cdc42Hs protein-serine kinase	Q07912	68.3
1242	NN022	Caveolin 2	Pan-specific	Caveolin 2	P51636	58.6
1182	NK183-1	Tyro10 (DDR2)	Pan-specific	Neurotrophic receptor-tyrosine kinase of discoidin domain receptor family, member 2 precursor	Q16832	55.4
1252	NK093	LIMK1	Pan-specific	LIM domain kinase 1	P53667	47.1
992	PN063	Rac1/cdc42	S71	Ras-related C3 botulinum toxin substrate 1	O75943	38.2
750	NK123	PAK3	Pan-specific	p21-activated serine kinase 3 (beta)	O75914	23.6
138	NN021	Catenin b	Pan-specific	Catenin (cadherin-associated protein) beta 1	P35222	21.6
452	PN103	Huntington	S421	Huntington's disease protein	P42858	20.0
746	NK122	PAK1	Pan-specific	p21-activated serine kinase 1 (alpha)	Q13153	18.2
766	PN059	Paxillin 1	Y31	Paxillin 1	P49023	9.1
1072	NN094	SIRPa1	Pan-specific	Signal regulatory protein substrate of PTP1D phosphatase (SHPS1)	P78324	8.9
764	PN060-2	Paxillin 1	Y118	Paxillin 1	P49023	8.8
50	PN008	B23 (NPM)	T199	B23 (nucleophosmin, numatrin, nucleolar protein NO38)	P06748	7.7
544	NK090	Ksr1	Pan-specific	Protein-serine kinase suppressor of Ras 1	Q8IVT5	2.0

1030	PK116	mTOR (FRAP)	S2448	Mammalian target of rapamycin (FRAP)	P42345	-1.7
792	PN051-2	MLC(MLR C2)	S20	Myosin regulatory light chain 2, smooth muscle isoform	O14950	-2.0
752	NK190	PAK5	Pan-specific	p21-activated serine kinase 5 (Serine/threonine-protein kinase PAK 7)	Q9P286	-3.2
114	NK023	CASK/Lin2	Pan-specific	Calcium/calmodulin-dependent protein-serine kinase (Lin2 homolog)	O14936	-7.7
748	PK061	PAK1/2/3	S144/S141/ S154	p21-activated protein-serine kinase 1/2/3	Q13153	-10.4
964	NK151	PRKWNK4	Pan-specific	Putative protein-serine kinase WNK4	Q96J92	-15.9
1184	NK183-2	Tyro10 (DDR2)	Pan-specific	Neurotrophic receptor-tyrosine kinase of discoidin domain receptor family, member 2 precursor	Q16832	-16.4
762	PN060-1	Paxillin 1	Y118	Paxillin 1	P49023	-16.5
42	PK115	ATM	S1981	Ataxia telangiectasia mutated	Q13315	-16.5
754	NK124	PAK6	Pan-specific	p21-activated serine kinase 6	Q9NQU5	-20.2
1022	NK159	ROKa (ROCK2)	Pan-specific	RhoA protein-serine kinase alpha	O75116	-20.2
206	PN020	Cofilin 2	S3	Cofilin 2	Q9Y281	-24.3
374	NN046	GroEL	Pan-specific	GroEL homolog (may correspond to Hsp60)	P10809	-25.4
744	NN084	PACSIN1	Pan-specific	Protein kinase C + casein kinase substrate in neurons protein 1	Q9BY11	-26.5
366	NN045	GNB2L1	Pan-specific	Guanine nucleotide-binding protein beta (receptor for activated C kinase 1 (RACK1))	P63244	-27.6
88	PN015	Caldesmon	S789	Caldesmon	Q05682	-28.4
52	PN009	B23 (NPM)	T234+T237	B23 (nucleophosmin, numatrin, nucleolar protein NO38)	P06748	-31.2
438	NN059-1	Hsp60	Pan-specific	Heat shock 60 kDa protein 1 (chaperonin, CPN60)	P10809	-31.3
674	PN052	MYPT1	T696	Myosin phosphatase target 1	O14974	-36.2
204	PN019	Cofilin 1	S3	Cofilin 1	P23528	-37.7
360	NK066	GCK	Pan-specific	Germinal centre protein-serine kinase	Q12851	-47.0
440	NN059-2	Hsp60	Pan-specific	Heat shock 60 kDa protein 1 (chaperonin, CPN60)	P10809	-58.4
702	NN075	NT5E	Pan-specific	Ecto-5'-nucleotidase (CD73 antigen)	P21589	-60.4
142	PN017	Caveolin 2	S23	Caveolin 2	P51636	-79.1

Translation						
272	PN029	eIF2Be	S540	Eukaryotic translation initiation factor 2B epsilon	Q13144	273.3
954	NP022	PP6C	Pan-specific	Protein-serine phosphatase 6 - catalytic subunit (PPVC)	O00743	181.6
934	NP010	PP1/Cb	Pan-specific	Protein-serine phosphatase 1 - catalytic subunit - beta isoform	P62140	139.2
274	PN030-1	eIF4E	S209	Eukaryotic translation initiation factor 4 (mRNA cap binding protein)	P06730	124.3
938	NP012	PP2A/Aa/b	Pan-specific	Protein-serine phosphatase 2A - A regulatory subunit - alpha and beta isoforms	P30153	122.2
944	NP016-NP031	PP2Ca/b	Pan-specific	Protein-serine phosphatase 2C - catalytic subunit - alpha/beta	P35813	110.7
936	NP011	PP1/Cg	Pan-specific	Protein-serine phosphatase 1 - catalytic subunit - gamma isoform	P36873	99.8
1246	NN039	eIF4E	Pan-specific	Eukaryotic translation initiation factor 4 (mRNA cap binding protein)	P06730	80.6
940	NP013-NP014	PP2A/Ca	Pan-specific	Protein-serine phosphatase 2A - catalytic subunit alpha isoform	P67775	75.2
268	PN028-1	eIF2a	S51	Eukaryotic translation initiation factor 2 alpha	P05198	72.2
950	NP020	PP4C	Pan-specific	Protein-serine phosphatase X - catalytic subunit (PPX/C)	P60510	69.0
948	NP019	PP4/A’2	Pan-specific	Protein-serine phosphatase 4 - regulatory subunit (PPX/A'2)	Q8TF05	69.0
266	NN038	eIF2a	Pan-specific	Eukaryotic translation initiation factor 2 alpha	P05198	67.7
276	PN030-2	eIF4E	S209	Eukaryotic translation initiation factor 4 (mRNA cap binding protein)	P06730	56.6
952	NP021	PP5C	Pan-specific	Protein-serine phosphatase 5 - catalytic subunit (PPT)	P53041	52.2
920	PK094	PKR1	T451	Double stranded RNA dependent protein-serine kinase	P19525	50.2
946	NP018	PP2Cd	Pan-specific	Protein-serine phosphatase 2C - catalytic subunit - delta isoform	O15297	46.5
278	PN031	eIF4G	S1107	Eukaryotic translation initiation factor 4 gamma 1	Q04637	46.1
270	PN028-2	eIF2a	S51	Eukaryotic translation initiation factor 2 alpha	P05198	37.3
942	NP015	PP2B/Aa	Pan-specific	Protein-serine phosphatase 2B - catalytic subunit - alpha isoform	Q08209	26.7
918	NK144-1	PKR1	Pan-specific	Double stranded RNA	P19525	3.8

				dependent protein-serine kinase		
968	NK152	PRP4K	Pan-specific	Protein-serine kinase PRP4 homolog	P60484	-30.3
770	NN087	PCNA	Pan-specific	Proliferating cell nuclear antigen	P12004	-31.2
			**Apoptosis**			
254	NK050	DRAK2	Pan-specific	DAP kinase-related apoptosis-inducing protein-serine kinase 2 (STK17B)	O94768	270.9
234	NN033	CytoC	Pan-specific	Cytochrome C	P99999	93.8
56	PN011	Bad	S91	Bcl2-antagonist of cell death protein	Q92934	93.8
112	NN010	CAS	Pan-specific	Cellular apoptosis susceptibility protein (CSE1L)	P55060	93.4
1270	NK160	ROKb (ROCK1)	Pan-specific	Rho-associated protein kinase 1	Q13464	80.3
58	NN007	Bcl-xL	Pan-specific	Bcl2-like protein 1	Q07817	61.8
118	NN012	CASP2	Pan-specific	Pro-caspase 2 (ICH1 protease)	P42575	57.0
1262	NK157	RIP2/RICK	Pan-specific	Receptor-interacting serine/threonine-protein kinase 2 (RIPK2)	O43353	55.9
240	NK046	DAPK2	Pan-specific	Death-associated protein kinase 2	Q9UIK4	52.3
318	PK020	FAK	S722	Focal adhesion protein-tyrosine kinase	Q05397	51.2
92	NK016-2	CaMK1d	Pan-specific	Calcium/calmodulin-dependent protein-serine kinase 1 delta	Q8IU85	38.8
736	PN057-1	p53	S392	Tumor suppressor protein p53 (antigenNY-CO-13)	P04637	36.0
1076	NN095	Smac/DIAB LO	Pan-specific	Second mitochondria-derived activator of caspase	Q9NR28	32.4
60	NN000	Bak	Pan-specific	Bcl2 homologous antagonist/killer (BCK2L7)	Q16611	31.4
1170	NN110	TRADD	Pan-specific	Tumor necrosis factor receptor type 1 associated DEATH domain protein	Q15628	29.3
542	NK113-3	MST1	Pan-specific	Mammalian STE20-like protein-serine kinase 1 (KRS2)	Q13043	29.1
238	NK045	DAPK1	Pan-specific	Death-associated protein kinase 1	P53355	28.2
116	NN011-NN125	CASP1	Pan-specific	Pro-caspase 1 (Interleukin-1 beta convertase) alpha/beta isoform	P29466	26.8
1216	NK188-1	ZIPK	Pan-specific	ZIP kinase (death associated protein-serine kinase 3 (DAPK3))	O43293	20.0
136	NN020	CASP12	Pan-specific	Pro-caspase 12 (mouse)	O08736	16.6
330	PK018-1	FAK	Y576	Focal adhesion protein-	Q05397	15.5

				tyrosine kinase		
328	PK019-2	FAK	Y577	Focal adhesion protein-tyrosine kinase	Q05397	15.3
584	NN123	p73	Pan-specific	Tumor suppressor protein p73	O15350	15.0
1220	NK188-2	ZIPK	Pan-specific	ZIP kinase (death associated protein-serine kinase 3 (DAPK3))	O43293	13.5
1204	NN112	XIAP	Pan-specific	X-linked inhibitor of apoptosis protein (baculoviral IAP repeat-containing 4)	P98170	12.9
122	NN014	CASP4	Pan-specific	Pro-caspase 4 (ICH2 protease, ICE(rel)-II)	P49662	12.9
134	NN019	CASP9	Pan-specific	Pro-caspase 9 (ICE-like apoptotic protease 6 (ICE-LAP6), Mch6, APAF3)	P55211	12.0
54	PN010	Bad	S75	Bcl2-antagonist of cell death protein	Q92934	11.1
16	NN002	AIF	Pan-specific	Apoptosis inducing factor (programed cell death protein 8 (PDCD8))	O95831	7.1
734	NN082	p53	Pan-specific	Tumor suppressor protein p53 (antigenNY-CO-13)	P04637	5.9
740	PN057-3	p53	S392	Tumor suppressor protein p53 (antigenNY-CO-13)	P04637	3.0
1018	NK158	RIPK1	Pan-specific	Receptor-interacting protein-serine kinase 1	Q13546	2.4
252	NK049	DRAK1	Pan-specific	DAP kinase-related apoptosis-inducing protein-serine kinase 1 (STK17A)	Q9UEE5	0.5
336	PK023	FAK	Y861	Focal adhesion protein-tyrosine kinase	Q05397	-2.2
110	NK022	CaMKK (CaMKK2)	Pan-specific	Calmodulin-dependent protein-serine kinase kinase	Q8N5S9	-5.0
64	NN006	Bcl2	Pan-specific	B-cell lymphoma protein 2 alpha	P10415	-6.0
1068	PN074	Shc1	Y349+Y350	SH2 domain-containing transforming protein 1	P29353	-8.9
320	PK021	FAK	S732	Focal adhesion protein-tyrosine kinase	Q05397	-9.3
90	NK016-1	CaMK1d	Pan-specific	Calcium/calmodulin-dependent protein-serine kinase 1 delta	Q8IU85	-11.1
340	NN043	FasL	Pan-specific	Tumor necrosis factor ligand, member 6	P48023	-11.1
36	NK007	ASK1 (MAP3K5)	Pan-specific	Apoptosis signal regulating protein-serine kinase	Q99683	-12.8
324	PK024	FAK	S910	Focal adhesion protein-tyrosine kinase	Q05397	-13.5
738	PN057-2	p53	S392	Tumor suppressor protein p53 (antigenNY-CO-13)	P04637	-13.7
972	PP002	PTEN	S370	Phosphatidylinositol-3,4,5-trisphosphate 3-phosphatase and protein phosphatase and	P60484	-13.8

				tensin homolog deleted on chromosome 10		
42	PK115	ATM	S1981	Ataxia telangiectasia mutated	Q13315	-16.5
1086	NN099	SODD	Pan-specific	Silencer of death domains (Bcl2 associated athanogene 4 (BAG4))	O95429	-17.4
120	NN013	CASP3	Pan-specific	Pro-caspase 3 (apopain, cysteine protease CPP32)	P42574	-20.9
338	NN042	FAS	Pan-specific	Tumor necrosis factor superfamily member 6 (Apo1, CD95)	P25445	-21.9
668	NK113-2	MST1	Pan-specific	Mammalian STE20-like protein-serine kinase 1 (KRS2)	Q13043	-25.0
326	PK017	FAK	Y397	Focal adhesion protein-tyrosine kinase	Q05397	-27.7
130	NN018	CASP8	Pan-specific	Pro-caspase 8 (ICE-like apoptotic protease 5 (ICE-LAP5), Mch5, FLICE, CAP4)	Q14790	-30.6
124	NN015	CASP5	Pan-specific	Caspase 5 (ICH3 protease, ICE(rel)-III)	P51878	-31.0
316	NK060	FAK	Pan-specific	Focal adhesion protein-tyrosine kinase	Q05397	-32.3
698	NN074	NME7	Pan-specific	Nucleotide diphosphate kinase 7 (nm23-H7)	Q9Y5B8	-32.6
66	NN008	Bcl-xS/L	Pan-specific	Bcl-xS/L	Q07817	-33.5
666	NK113-1	MST1	Pan-specific	Mammalian STE20-like protein-serine kinase 1 (KRS2)	Q13043	-36.8
670	NK114	MST2	Pan-specific	Mammalian STE20-like protein-serine kinase 2 (KRS1)	Q13188	-37.3
1172	NN111	Trail	Pan-specific	Tumor necrosis factor-related apoptosis-inducing ligand	P50591	-39.6
696	NN073	NME6	Pan-specific	Nucleotide diphosphate kinase 6 (nm23-H6)	O75414	-41.1
974	PN052	PTEN	S380+S382 +S385	Phosphatidylinositol-3,4,5-trisphosphate 3-phosphatase and protein phosphatase and tensin homolog deleted on chromosome 10	P18031	-41.7
244	NN035-NN126	DFF45/35	Pan-specific	DNA fragmentation factor alpha (ICAD) 45/35-kDa subunit	O00273	-42.4
68	NN009	Bid	Pan-specific	BH3 interacting domain death agonist	P55957	-42.4
692	NN072	Nip1	Pan-specific	Bcl2/adenovirus E1B 19kD-interacting protein 1	Q12981	-44.9
1074	NK171	SLK	Pan-specific	STE20-like protein-serine kinase	Q9H2G2	-45.4
334	PK019-1	FAK	Y577	Focal adhesion protein-tyrosine kinase	Q05397	-45.4

322	PK022	FAK	S843	Focal adhesion protein-tyrosine kinase	Q05397	-46.2
128	NN017	CASP7	Pan-specific	Pro-caspase 7 (ICE-like apoptotic protease 3 (ICE-LAP3), Mch3)	P55210	-47.6
62	NN005	Bax	Pan-specific	Apoptosis regulator Bcl2-associated X protein	Q07812	-49.7
332	PK018-2	FAK	Y576	Focal adhesion protein-tyrosine kinase	Q05397	-56.8
194	NN025	c-IAP1	Pan-specific	Cellular inhibitor of apoptosis protein 1 (baculoviral IAP repeat-containing protein 3, apoptosis inhibitor 2 (API2))	Q13490	-57.2
970	NP023	PTEN	Pan-specific	Phosphatidylinositol-3,4,5-trisphosphate 3-phosphatase and protein phosphatase and tensin homolog deleted on chromosome 10	P60484	-69.7
126	NN016	CASP6	Pan-specific	Pro-caspase 6 (apoptotic protease Mch2)	P55212	-71.1
242	NN034	DAXX	Pan-specific	Death-associated protein 6 (BING2)	Q9UER7	-72.8
			**Cell growth and division**			
150	NP003	Cdc25C	Pan-specific	Cell division cycle 25C phosphatase	P30307	248.2
264	PK010	EGFR	Y1148	Epidermal growth factor receptor-tyrosine kinase	P00533	232.0
208	PN022	Cortactin	Y470	Cortactin (amplaxin) (mouse)	Q60598	192.7
954	NP022	PP6C	Pan-specific	Protein-serine phosphatase 6 - catalytic subunit (PPVC)	O00743	181.6
228	NN030	Cyclin D1	Pan-specific	Cyclin D1 (PRAD1)	P24385	156.8
934	NP010	PP1/Cb	Pan-specific	Protein-serine phosphatase 1 - catalytic subunit - beta isoform	P62140	139.2
170	PK007-3	CDK1/2	Y15	Cyclin-dependent protein-serine kinase 1/2	P06493	123.7
938	NP012	PP2A/Aa/b	Pan-specific	Protein-serine phosphatase 2A - A regulatory subunit - alpha and beta isoforms	P30153	122.2
924	PK117	Plk1	T210	Polo-like protein-serine kinase 1	P53350	113.4
944	NP016-NP031	PP2Ca/b	Pan-specific	Protein-serine phosphatase 2C - catalytic subunit - alpha/beta	P35813	110.7
936	NP011	PP1/Cg	Pan-specific	Protein-serine phosphatase 1 - catalytic subunit - gamma isoform	P36873	99.8
250	PN027	Dok2	Y142	Docking protein 2 (mouse)	O60496	96.8
186	NK032	CDK9	Pan-specific	Cyclin-dependent protein-serine kinase 9	P50750	85.1
310	NK059-1	p38g MAPK	Pan-specific	Mitogen-activated protein-	P53778	80.4

		(Erk6)		serine kinase p38 gamma (MAPK12)		
940	NP013-NP014	PP2A/Ca	Pan-specific	Protein-serine phosphatase 2A - catalytic subunit alpha isoform	P67775	75.2
950	NP020	PP4C	Pan-specific	Protein-serine phosphatase X - catalytic subunit (PPX/C)	P60510	69.0
948	NP019	PP4/A’2	Pan-specific	Protein-serine phosphatase 4 - regulatory subunit (PPX/A'2)	Q8TF05	69.0
230	NN031	Cyclin E	Pan-specific	Cyclin E1	P24864	65.4
260	NK052-2	EGFR	Pan-specific	Epidermal growth factor receptor-tyrosine kinase	P00533	64.4
930	NP009	PP1/Ca	Pan-specific	Protein-serine phosphatase 1 - catalytic subunit - alpha isoform	P62136	56.7
952	NP021	PP5C	Pan-specific	Protein-serine phosphatase 5 - catalytic subunit (PPT)	P53041	52.2
664	PK058	Msk1	S376	Mitogen & stress-activated protein-serine kinase 1	O75582	51.6
532	NP004	KAP	Pan-specific	Cyclin-dependent kinase associated phosphatase (CDK inhibitor 3, CIP2)	Q16667	51.5
318	PK020	FAK	S722	Focal adhesion protein-tyrosine kinase	Q05397	51.2
476	NK078-2	ILK1	Pan-specific	Integrin-linked protein-serine kinase 1	Q13418	50.8
154	NN023	Cdc34	Pan-specific	Cell division cycle 34 (ubiquitin-conjugating ligase)	P49427	48.5
946	NP018	PP2Cd	Pan-specific	Protein-serine phosphatase 2C - catalytic subunit - delta isoform	O15297	46.5
262	PK009	EGFR	Y1068	Epidermal growth factor receptor-tyrosine kinase	P00533	45.8
932	PP001	PP1/Ca	T320	Protein-serine phosphatase 1 - catalytic subunit - alpha isoform	P62136	45.4
258	NK052-1	EGFR	Pan-specific	Epidermal growth factor receptor-tyrosine kinase	P00533	44.1
926	NK146	Plk2	Pan-specific	Polo-like protein kinase 2 (serum -inducible kinase (SNK))	Q9NYY3	42.3
304	NK057	Erk3	Pan-specific	Extracellular regulated protein-serine kinase 3	Q16659	41.0
1236	NN024	Cdc42	Pan-specific	Cell division control protein 42 homolog	P60953	39.5
298	PK014-PK015-2	Erk1/2	T202+Y204 /T185+ Y187	Extracellular regulated protein-serine kinase 1/2 (p44/p42 MAP kinases)	P27361	36.9
226	NN029	Cyclin B1	Pan-specific	Cyclin B1	P14635	36.4
308	PK016	Erk5	T218+Y220	Extracellular regulated protein-serine kinase 5 (Big MAP kinase 1 (BMK1))	P53778	34.3

548	NK091	LATS1	Pan-specific	Large tumor suppressor 1 protein-serine kinase (WARTS)	O95835	32.8
922	NK145	Plk1	Pan-specific	Polo-like protein-serine kinase 1	P53350	31.6
224	NN028	Cyclin A	Pan-specific	Cyclin A1	P78396	27.9
942	NP015	PP2B/Aa	Pan-specific	Protein-serine phosphatase 2B - catalytic subunit - alpha isoform	Q08209	26.7
152	NK024	CDC2L5 (CHED)	Pan-specific	Cell division cycle 2-like protein-serine kinase 5	Q14004	26.5
288	PK011	EGFR	Y1173	Epidermal growth factor receptor-tyrosine kinase	P00533	26.3
1168	NK177	Tlk1	Pan-specific	Tousled-like protein-serine kinase 1	Q9UKI8	24.0
6	NK001	Abl	Pan-specific	Abelson proto-oncogene-encoded protein-tyrosine kinase	P00519	22.3
1202	NK185	Wee1	Pan-specific	Wee1 protein-tyrosine kinase	P30291	21.8
294	NK055-NK056	Erk1/2	Pan-specific	Extracellular regulated protein-serine kinase 1/2 (p44/p42 MAP kinases)	P27361	20.4
684	NK119	Nek7	Pan-specific	NIMA (never-in-mitosis)-related protein-serine kinase 7	Q8TDX7	20.3
158	NK026-1	CDK2	Pan-specific	Cyclin-dependent protein-serine kinase 2	P24941	20.2
1002	NN093	Rb	Pan-specific	Retinoblastoma-associated protein 1	P06400	19.9
188	NK033	CDK10	Pan-specific	Cyclin-dependent protein-serine kinase 10 PISSLRE	Q15131	18.9
330	PK018-1	FAK	Y576	Focal adhesion protein-tyrosine kinase	Q05397	15.5
328	PK019-2	FAK	Y577	Focal adhesion protein-tyrosine kinase	Q05397	15.3
78	PN014	BRCA1	S1497	Breast cancer type 1 susceptibility protein	P38398	14.5
300	PK014-PK015-3	Erk1/2	T202+Y204/T185+Y187	Extracellular regulated protein-serine kinase 1/2 (p44/p42 MAP kinases)	P27361	13.0
732	NP021	PP5C	Pan-specific	Protein-serine phosphatase 5 - catalytic subunit (PPT)	P53041	11.9
156	NK025-3	CDK1 (CDC2)	Pan-specific	Cyclin-dependent protein-serine kinase 1	P06493	11.6
640	NK110	Met	Pan-specific	Hepatocyte growth factor (HGF) receptor-tyrosine kinase	P08581	9.6
1072	NN094	SIRPa1	Pan-specific	Signal regulatory protein substrate of PTP1D phosphatase (SHPS1)	P78324	8.9
190	NK034	Chk1	Pan-specific	Checkpoint protein-serine kinase 1	O14757	8.8
650	PK056	MLK3	T277+S281	Mixed-lineage protein-serine kinase 3	Q16584	7.2

1178	NK180	TTK	Pan-specific	Dual specificity protein kinase	P33981	7.1
20	NN003	AK2	Pan-specific	Adenylate kinase 2	P54819	5.4
390	NK071	Haspin	Pan-specific	Haploid germ cell-specific nuclear protein-serine kinase	Q8TF76	1.9
8	PK001	Abl	Y412	Abelson proto-oncogene-encoded protein-tyrosine kinase	P00519	-0.8
928	NK147	Plk3	Pan-specific	Polo-like protein kinase 3 (cytokine- inducible kinase (CNK))	Q9H4B4	-1.0
1030	PK116	mTOR (FRAP)	S2448	Mammalian target of rapamycin (FRAP)	P42345	-1.7
336	PK023	FAK	Y861	Focal adhesion protein-tyrosine kinase	Q05397	-2.2
210	NK042	COT	Pan-specific	Osaka thyroid oncogene protein-serine kinase (Tpl2)	P41279	-2.3
678	NK117-2	Nek2	Pan-specific	NIMA (never-in-mitosis)-related protein-serine kinase 2	P51955	-3.5
18	NK008-1	Aurora A (AIK)	Pan-specific	Aurora Kinase A (serine/threonine protein kinase 6)	Q96GD4	-5.9
350	NK064	FLT4	Pan-specific	Vascular endothelial growth factor receptor-protein-tyrosine kinase 3 (VEGFR3)	P35916	-7.5
160	NK026-2	CDK2	Pan-specific	Cyclin-dependent protein-serine kinase 2	P24941	-8.1
680	NK117-3	Nek2	Pan-specific	NIMA (never-in-mitosis)-related protein-serine kinase 2	P51955	-8.1
1068	PN074	Shc1	Y349+ Y350	SH2 domain-containing transforming protein 1	P29353	-8.9
320	PK021	FAK	S732	Focal adhesion protein-tyrosine kinase	Q05397	-9.3
1052	PN073	S6	S235	40S ribosomal protein S6	P62753	-11.4
1012	PN071	Rb	T826	Retinoblastoma-associated protein 1	P06400	-12.9
164	PK006	CDK1/2	T14+Y15	Cyclin-dependent protein-serine kinase 1/2	P06493	-13.2
324	PK024	FAK	S910	Focal adhesion protein-tyrosine kinase	Q05397	-13.5
296	PK014-PK015-1	Erk1/2	T202+Y204 /T185+Y187	Extracellular regulated protein-serine kinase 1/2 (p44/p42 MAP kinases)	P27361	-14.4
42	PK115	ATM	S1981	Ataxia telangiectasia mutated	Q13315	-16.5
474	NK078-1	ILK1	Pan-specific	Integrin-linked protein-serine kinase 1	Q13418	-18.7
182	NK025-2	CDK1 (CDC2)	Pan-specific	Cyclin-dependent protein-serine kinase 1	P06493	-19.3
348	PN032	FKHRL1	T32	Forkhead-like transcription factor 1 (FOXO3A)	O43524	-19.7
232	NN032	Cyclin G1	Pan-specific	Cyclin G1	P51959	-20.6
306	NK058	Erk4	Pan-specific	Extracellular regulated	Q13164	-21.5

				protein-serine kinase 4		
682	NK118	Nek4	Pan-specific	NIMA (never-in-mitosis)-related protein-serine kinase 4	P51957	-23.6
180	NK030-2	CDK7	Pan-specific	Cyclin-dependent protein-serine kinase 7	P50613	-23.7
376	NN047	Grp75	Pan-specific	Glucose regulated protein 75	P38646	-25.0
344	NK062	FGFR1	Pan-specific	Fibroblast growth factor receptor-tyrosine kinase 1	P11362	-25.2
1024	NK162	ROR2	Pan-specific	ROR2 neurotrophic receptor-tyrosine kinase	Q01974	-25.7
356	NK065	Fyn	Pan-specific	Fyn proto-oncogene-encoded protein-tyrosine kinase	P06241	-26.0
346	NK063	FGFR2	Pan-specific	Fibroblast growth factor receptor-tyrosine kinase 2 (BEK)	P21802	-26.6
1164	NK169	TEK (TIE2)	Pan-specific	Angiopoietin-1 receptor-tyrosine kinase	Q02763	-26.7
326	PK017	FAK	Y397	Focal adhesion protein-tyrosine kinase	Q05397	-27.7
88	PN015	Caldesmon	S789	Caldesmon	Q05682	-28.4
1000	NK156	RafB (Braf)	Pan-specific	RafB proto-oncogene-encoded protein-serine kinase	P06400	-28.7
1010	PN070	Rb	T821	Retinoblastoma-associated protein 1	P06400	-29.0
302	NK056	Erk2	Pan-specific	Extracellular regulated protein-serine kinase 2 (p42 MAP kinase)	P28482	-29.7
982	NK025-1	CDK1 (CDC2)	Pan-specific	Cyclin-dependent protein-serine kinase 1	Q05209	-30.5
44	NK008-2	Aurora A (AIK)	Pan-specific	Aurora Kinase A (serine/threonine protein kinase 6)	Q96GD4	-31.3
192	NK035	Chk2	Pan-specific	Checkpoint protein-serine kinase 2	O96017	-31.8
994	PN064	Rad17	S645	Rad17 homolog	P04049	-32.3
316	NK060	FAK	Pan-specific	Focal adhesion protein-tyrosine kinase	Q05397	-32.3
1014	PN067	Rb	S780	Retinoblastoma-associated protein 1	P07949	-34.9
996	NK155	Raf1	Pan-specific	Raf1 proto-oncogene-encoded protein-serine kinase	P04049	-35.0
46	NK009	Aurora C (AIK3)	Pan-specific	Aurora Kinase C (serine/threonine-protein kinase 13)	Q9UQB9	-35.2
1028	NK126-1	PDK1	Pan-specific	3-phosphoinositide-dependent protein-serine kinase 1	O15530	-36.3
704	NN083	p107	Pan-specific	Retinoblastoma (Rb) protein-related p107 (PRB1)	P28749	-36.9
712	NN080	p27 Kip1	Pan-specific	p27 cyclin-dependent kinase inhibitor 1B	P46527	-36.9

450	NK193	Aurora B (AIM-1)	Pan-specific	Aurora Kinase B (serine/threonine protein kinase 12)	Q96GD4	-38.3
1006	PN068	Rb	S807	Retinoblastoma-associated protein 1	P06400	-38.3
644	PK055	Met	Y1230+Y1234+Y1235	Hepatocyte growth factor (HGF) receptor-tyrosine kinase	P08581	-38.3
998	PK098	Raf1	S259	Raf1 proto-oncogene-encoded protein-serine kinase	P15056	-38.6
1050	NK167	RYK	Pan-specific	RYK tyrosine-protein kinase	P34925	-39.5
1020	PN065	Rb	T356	Retinoblastoma-associated protein 1	P06400	-39.5
662	NN069	MSH2	Pan-specific	DNA mismatch repair protein mutS homolog2, colon cancer, nonpolyposis type 1	P43246	-40.0
162	PK007-1	CDK1/2	Y15	Cyclin-dependent protein-serine kinase 1/2	P06493	-42.2
364	NK026-3	CDK2	Pan-specific	Cyclin-dependent protein-serine kinase 2	P24941	-42.4
354	PN033	Fos	T232	Fos-c FBJ murine osteosarcoma oncoprotein-related transcription factor	P01100	-42.6
760	PN058	Pax2	S394	Paired box protein 2	Q02962	-42.8
1026	NK163	ROS	Pan-specific	Orosomucoid 1 receptor-tyrosine kinase	P08922	-43.0
1004	PN066	Rb	S612	Retinoblastoma-associated protein 1	P06400	-44.7
334	PK019-1	FAK	Y577	Focal adhesion protein-tyrosine kinase	Q05397	-45.4
1008	PN069	Rb	S807+S811	Retinoblastoma-associated protein 1	P06400	-45.7
672	NK116	mTOR (FRAP)	Pan-specific	Mammalian target of rapamycin (FRAP)	P42345	-45.9
178	NK030-1	CDK7	Pan-specific	Cyclin-dependent protein-serine kinase 7	P50613	-46.1
708	NN077	p18 INK4c	Pan-specific	p18 INK4c cyclin-dependent kinase inhibitor	P42773	-46.2
322	PK022	FAK	S843	Focal adhesion protein-tyrosine kinase	Q05397	-46.2
148	NP002	Cdc25B	Pan-specific	Cell division cycle 25B phosphatase	P30305	-46.9
710	NN078	p21 CDKI1	Pan-specific	Cyclin-dependent kinase inhibitor 1 (MDA6)	P38936	-48.2
714	PN056	p27 Kip1	T187	p27 cyclin-dependent kinase inhibitor 1B	P46527	-48.7
642	PK054	Met	Y1003	Hepatocyte growth factor (HGF) receptor-tyrosine kinase	P08581	-50.9
86	NK015	BUB1A	Pan-specific	BUB1 mitotic checkpoint protein-serine kinase	O43683	-51.4
172	NK027	CDK4	Pan-specific	Cyclin-dependent protein-serine kinase 4	P11802	-55.1

352	NN044	Fos	Pan-specific	Fos-c FBJ murine osteosarcoma oncoprotein-related transcription factor	P01100	-56.0
184	NK031-2	CDK8	Pan-specific	Cyclin-dependent protein-serine kinase 8	P49336	-56.6
332	PK018-2	FAK	Y576	Focal adhesion protein-tyrosine kinase	Q05397	-56.8
174	NK028	CDK5	Pan-specific	Cyclin-dependent protein-serine kinase 5	Q00535	-60.2
676	NK117-1	Nek2	Pan-specific	NIMA (never-in-mitosis)-related protein-serine kinase 2	P51955	-60.6
166	PK008	CDK1/2	T161/T160	Cyclin-dependent protein-serine kinase 1/2	P06493	-64.0
168	PK007-2	CDK1/2	Y15	Cyclin-dependent protein-serine kinase 1/2	P06493	-64.1
176	NK029	CDK6	Pan-specific	Cyclin-dependent protein-serine kinase 6	Q00534	-65.1
706	NN076	p16 INK4	Pan-specific	p16 INK4a cyclin-dependent kinase inhibitor (MTS1)	P42771	-66.4
**Adhesion**						
554	PK039	Lck	S157	Lymphocyte-specific protein-tyrosine kinase	P06239	93.8
546	NP005	LAR	Pan-specific	LCA antigen-related (LAR) receptor tyrosine phosphatase	P10586	83.1
556	PK040	Lck	Y191	Lymphocyte-specific protein-tyrosine kinase	P06239	67.0
1256	NK182	Tyro3	Pan-specific	Tyrosine-protein kinase receptor TYRO3	Q06418	65.1
956	PN062	PRAS40	T246	Proline-rich Akt substrate 40 kDa (Akt1S1)	Q96B36	52.7
1198	PN095	Vinculin	Y821	Vinculin	P18206	36.1
552	NK092-2	Lck	Pan-specific	Lymphocyte-specific protein-tyrosine kinase	P06239	14.6
790	NN088	PERP	Pan-specific	p53-induced protein PIGPC1	Q9H230	12.2
550	NK092-1	Lck	Pan-specific	Lymphocyte-specific protein-tyrosine kinase	P06239	3.5
478	PN043	Integrin a4	S988	Integrin alpha 4 (VLA4)	P13612	1.1
480	PN044	Integrin b1	S785	Integrin beta 1 (fibronectin receptor beta subunit, CD29 antigen)	P05556	-12.5
482	PN105	Integrin b1	Y783	Integrin beta 1 (fibronectin receptor beta subunit, CD29 antigen)	P05556	-15.8
1196	PN094	Vimentin	S33	Vimentin	P08670	-16.1
558	PK041	Lck	Y504	Lymphocyte-specific protein-tyrosine kinase	P06239	-17.5
**B-cell**						
82	NK014	Btk	Pan-specific	Bruton's agammaglobulinemia	Q06187	83.1

				tyrosine kinase		
84	PK004	Btk	Y223	Bruton's agammaglobulinemia tyrosine kinase	Q06187	4.2
70	NK011	BLK	Pan-specific	B lymphoid tyrosine kinase	P51451	0.6
			**Heme**			
414	NK072	Hpk1	Pan-specific	Hematopoetic progenitor protein-serine kinase 1	Q92918	9.6
412	NN053	HO2	Pan-specific	Heme oxygenase 2	P30519	-10.9
1190	PK110	VEGFR2 (KDR)	Y1054	Vascular endothelial growth factor receptor-tyrosine kinase 2 (Flk1)	P35968	-14.2
454	NK073	ICK	Pan-specific	Intestinal cell protein-serine kinase (MAK-related kinase (MRK))	Q9UPZ9	-26.9
410	NN052	HO1	Pan-specific	Heme oxygenase 1	P09601	-48.6
1192	PK111	VEGFR2 (KDR)	Y1054+Y1059	Vascular endothelial growth factor receptor-tyrosine kinase 2 (Flk1)	P35968	-59.2
			**Neuro**			
236	PN026	Dab1	Y198	Disabled homolog 1	O75553	198.4
22	NK003	ALK	Pan-specific	Anaplastic lymphoma kinase	Q9UM73	91.3
1256	NK182	Tyro3	Pan-specific	Tyrosine-protein kinase receptor TYRO3	Q06418	65.1
284	NK054-2	ErbB2 (HER2)	Pan-specific	ErbB2 (Neu) receptor-tyrosine kinase	P04626	57.1
1146	PN106	Tau	S518	Microtubule-associated protein tau	P10636	30.7
1158	PN108	Tau	T547	Microtubule-associated protein tau	P10636	27.5
198	NK037	CK1e	Pan-specific	Casein protein-serine kinase 1 epsilon	P49674	25.8
1148	PN088	Tau	S530	Microtubule-associated protein tau	P10636	25.8
988	NK154	Pyk2	Pan-specific	Protein-tyrosine kinase 2	Q14289	21.7
1072	NN094	SIRPa1	Pan-specific	Signal regulatory protein substrate of PTP1D phosphatase (SHPS1)	P78324	8.9
1156	PN092	Tau	S720	Microtubule-associated protein tau	P10636	8.0
1186	PN109	Tyrosine Hydroxylase	S18	Tyrosine hydroxylase isoform a	P07101	5.5
1258	NK043	CPG16/CaM Kinase VI	Pan-specific	Serine/threonine-protein kinase DCAMKL1	O08875	5.3
1140	PN107	Tau	S738	Microtubule-associated protein tau	P10636	5.1
290	PK013-1	ErbB2 (HER2)	Y1248	ErbB2 (Neu) receptor-tyrosine kinase	P04626	2.5
98	PK005-2	CaMK2a	T286	Calcium/calmodulin-dependent protein-serine	Q9UQM7	1.7

				kinase 2 alpha		
1150	PN089	Tau	S578	Microtubule-associated protein tau	P10636	0.9
1174	NK178	TrkA	Pan-specific	Nerve growth factor (NGF) receptor- tyrosine kinase	P04629	-0.5
1142	PN085	Tau	S515	Microtubule-associated protein tau	P10636	-0.6
286	NK054-1	ErbB2 (HER2)	Pan-specific	ErbB2 (Neu) receptor-tyrosine kinase	P04626	-6.5
114	NK023	CASK/Lin2	Pan-specific	Calcium/calmodulin-dependent protein-serine kinase (Lin2 homolog)	O14936	-7.7
358	PN098	GAP-43	S41	Growth associated protein 43 (Neuromodulin)	P17677	-8.2
1134	PN084	Synapsin 1	S9	Synapsin 1 isoform Ia	P17600	-8.4
1152	PN090	Tau	S712	Microtubule-associated protein tau	P10636	-16.3
1144	PN086	Tau	S515+S518	Microtubule-associated protein tau	P10636	-17.6
292	PK013-2	ErbB2 (HER2)	Y1248	ErbB2 (Neu) receptor-tyrosine kinase	P04626	-18.7
716	NN081-NN120	p35	Pan-specific	CDK5 regulatory subunit 1, p35	Q15078	-18.9
104	NK019-1	CAMK2d	Pan-specific	Calcium/calmodulin-dependent protein-serine kinase 2 delta	Q13557	-22.0
102	NK018-2	CAMK2b	Pan-specific	Calcium/calmodulin-dependent protein-serine kinase 2 beta	Q13554	-22.7
1176	NK179	TrkB	Pan-specific	BNDF/NT3/4/5 receptor-tyrosine kinase	Q16620	-22.9
356	NK065	Fyn	Pan-specific	Fyn proto-oncogene-encoded protein-tyrosine kinase	P06241	-26.0
100	NK018-1	CAMK2b	Pan-specific	Calcium/calmodulin-dependent protein-serine kinase 2 beta	Q13554	-31.5
196	NK036	CK1d	Pan-specific	Casein protein-serine kinase 1 delta	P48730	-31.7
96	PK005-1	CaMK2a	T286	Calcium/calmodulin-dependent protein-serine kinase 2 alpha	Q9UQM7	-32.9
106	NK019-2	CAMK2d	Pan-specific	Calcium/calmodulin-dependent protein-serine kinase 2 delta	Q13557	-33.0
362	PN034	GFAP	S8	Glial fibrillary acidic protein	P14136	-33.1
108	NK020	CAMK2g	Pan-specific	Calcium/calmodulin-dependent protein-serine kinase 2 gamma	Q13555	-35.0
990	PK097	Pyk2	Y579	Protein-tyrosine kinase 2	P60953	-36.6
1154	PN091	Tau	S716	Microtubule-associated protein tau	P10636	-37.2
700	PN055	NR1	S896	N-methyl-D-aspartate (NMDA) glutamate receptor 1 subunit zeta	Q05586	-39.1

1188	PN093	Tyrosine Hydroxylase	S70	Tyrosine hydroxylase isoform a	P07101	-40.0
132	NK021	CaMK4	Pan-specific	Calcium/calmodulin-dependent protein-serine kinase 4	Q16566	-42.7
694	PN054	NMDAR2B	Y1474	N-methyl-D-aspartate (NMDA) glutamate receptor 2B subunit	Q13224	-48.6
1132	PN111	Synapsin 1	S605	Synapsin 1 isoform Ia	P17600	-54.2
**Adrenergic Receptor**						
34	PN005	Arrestin b1	S412	Arrestin beta 1	P49407	216.0
1234	PN005	Arrestin b1	Pan-specific	Arrestin beta 1	P49407	88.3
370	PK025	GRK2 (BARK1)	S670	G protein-coupled receptor-serine kinase 2	P25098	-28.4
368	NK067	GRK2 (BARK1)	Pan-specific	G protein-coupled receptor-serine kinase 2	P25098	-28.9
372	NK068	GRK3 (BARK2)	Pan-specific	G protein-coupled receptor-serine kinase 3	P35626	-38.7
**Calcium**						
804	NK127-1	PKA Ca/b	Pan-specific	cAMP-dependent protein-serine kinase catalytic subunit alpha/beta	P17612	162.6
914	NK148	PRK1 (PKN1)	Pan-specific	Protein kinase C-related protein-serine kinase 1	Q16512	143.0
536	PK036	Kit	Y703	Kit/Steel factor receptor-tyrosine kinase	P10721	118.6
892	PK089	PKCq	S676	Protein-serine kinase C theta	Q04759	108.9
852	NK135	PKCd	Pan-specific	Protein-serine kinase C delta	Q05655	96.9
884	NK138	PKCl/i	Pan-specific	Protein-serine kinase C lambda/iota	P41743	87.4
256	NK051	eEF2K	Pan-specific	Elongation factor-2 protein-serine kinase	O00418	78.8
900	PK091	PKCz/l	T410/T403	Protein-serine kinase C zeta/lambda	Q05513	70.9
1276	NN089	PI3K	Pan-specific	Phosphatidylinositol 3-kinase regulatory subunit alpha	P27986	69.3
916	NN116	PKA R1a	Pan-specific	cAMP-dependent protein kinase type I-alpha regulatory chain	P10644	66.6
930	NP009	PP1/Ca	Pan-specific	Protein-serine phosphatase 1 - catalytic subunit - alpha isoform	P62136	56.7
956	PN062	PRAS40	T246	Proline-rich Akt substrate 40 kDa (Akt1S1)	Q96B36	52.7
886	PK087	PKCl	T555	Protein-serine kinase C lambda/iota	P41743	48.8
932	PP001	PP1/Ca	T320	Protein-serine phosphatase 1 - catalytic subunit - alpha isoform	P62136	45.4
906	PK093-1	PKCm	S910	Protein-serine kinase C mu	Q15139	43.8

		(PKD)		(Protein kinase D)		
890	NK140	PKCq	Pan-specific	Protein-serine kinase C theta	Q04759	39.5
92	NK016-2	CaMK1d	Pan-specific	Calcium/calmodulin-dependent protein-serine kinase 1 delta	Q8IU85	38.8
896	PK088	PKCq	T538	Protein-serine kinase C theta	Q04759	38.3
814	PK069	PKA R2a	S98	cAMP-dependent protein-serine kinase regulatory type 2 subunit alpha	P13861	36.8
898	NK141	PKCz	Pan-specific	Protein-serine kinase C zeta	Q05513	34.6
816	PK070	PKA R2b	S114	cAMP-dependent protein-serine kinase regulatory type 2 subunit beta	P31323	34.0
910	NK143	PKG1	Pan-specific	Protein-serine kinase G1 (cGMP-dependent protein kinase)	Q13976	33.9
888	NK139	PKC-nu (PKN3)	Pan-specific	Protein-serine kinase C nu	O94806	33.0
860	PK077-1	PKCd	Y313	Protein-serine kinase C delta	Q05655	32.5
902	NK142	PKCm (PKD)	Pan-specific	Protein-serine kinase C mu (Protein kinase D)	Q15139	29.0
824	PK072-2	PKBa (Akt1)	S473	Protein-serine kinase B alpha	P31749	28.5
904	PK092	PKCm (PKD)	S738+S742	Protein-serine kinase C mu (Protein kinase D)	Q15139	25.7
850	PK076	PKCb2	T641	Protein-serine kinase C beta 2	P05771	25.6
882	PK085	PKCh	T655	Protein-serine kinase C eta	P24723	23.9
830	NK130-1	PKBb (Akt2)	Pan-specific	Protein-serine kinase B beta	P31751	22.8
988	NK154	Pyk2	Pan-specific	Protein-tyrosine kinase 2	Q14289	21.7
810	PK068	PKA Cb	S338	cAMP-dependent protein-serine kinase catalytic subunit beta	P22694	19.4
820	PK071-1	PKBa (Akt1)	T308	Protein-serine kinase B alpha	P31749	19.3
894	PK090	PKCq	S695	Protein-serine kinase C theta	Q04759	19.2
836	NK131-2	PKBg (Akt3)	Pan-specific	Protein-serine kinase B gamma	Q9Y243	18.1
1204	NN112	XIAP	Pan-specific	X-linked inhibitor of apoptosis protein (baculoviral IAP repeat-containing 4)	P98170	12.9
868	PK081-2	PKCe	S729	Protein-serine kinase C epsilon	Q02156	11.0
870	NK137	PKCg	Pan-specific	Protein-serine kinase C gamma	P05129	10.4
866	PK081-1	PKCe	S729	Protein-serine kinase C epsilon	Q02156	8.4
806	NK127-2	PKA Ca/b	Pan-specific	cAMP-dependent protein-serine kinase catalytic subunit alpha/beta	P17612	7.3
1258	NK043	CPG16/CaM	Pan-specific	Serine/threonine-protein	O08875	5.3

		Kinase VI		kinase DCAMKL1		
908	PK093-2	PKCm (PKD)	S910	Protein-serine kinase C mu (Protein kinase D)	Q15139	5.0
862	PK077-2	PKCd	Y313	Protein-serine kinase C delta	Q05655	4.9
826	PK072-3	PKBa (Akt1)	S473	Protein-serine kinase B alpha	P31749	4.2
838	NK132	PKCa	Pan-specific	Protein-serine kinase C alpha	P17252	3.3
98	PK005-2	CaMK2a	T286	Calcium/calmodulin-dependent protein-serine kinase 2 alpha	Q9UQM7	1.7
834	NK131-1	PKBg (Akt3)	Pan-specific	Protein-serine kinase B gamma	Q9Y243	0.5
840	PK073	PKCa	S657	Protein-serine kinase C alpha	P17252	0.4
858	PK078	PKCd	T507	Protein-serine kinase C delta	Q05655	-1.8
844	NK133	PKCb1	Pan-specific	Protein-serine kinase C beta 1	P05771	-2.7
794	NK191	PI3K p110 delta	Pan-specific	Phosphatidylinositol-4,5-biphosphate 3-kinase catalytic subunit delta isoform	O00329	-3.3
828	PK071-2	PKBa (Akt1)	T308	Protein-serine kinase B alpha	P31749	-3.3
818	NK129	PKBa (Akt1)	Pan-specific	Protein-serine kinase B alpha	P31749	-4.0
110	NK022	CaMKK (CaMKK2)	Pan-specific	Calmodulin-dependent protein-serine kinase kinase	Q8N5S9	-5.0
976	NP024	PTP1B	Pan-specific	Protein-tyrosine phosphatase 1B	P29350	-6.2
878	PK084	PKCg	T674	Protein-serine kinase C gamma	P05129	-7.2
880	PK086	PKCh	S674	Protein-serine kinase C eta	P24723	-7.5
114	NK023	CASK/Lin2	Pan-specific	Calcium/calmodulin-dependent protein-serine kinase (Lin2 homolog)	O14936	-7.7
848	NK134	PKCb2	Pan-specific	Protein-serine kinase C beta 2	P05771	-8.3
832	NK130-2	PKBb (Akt2)	Pan-specific	Protein-serine kinase B beta	P31751	-9.2
856	PK080	PKCd	S664	Protein-serine kinase C delta	Q05655	-10.9
784	PK066	PDK1	S244	3-Phosphoinositide-dependent protein-serine kinase 1	O15530	-10.9
90	NK016-1	CaMK1d	Pan-specific	Calcium/calmodulin-dependent protein-serine kinase 1 delta	Q8IU85	-11.1
972	PP002	PTEN	S370	Phosphatidylinositol-3,4,5-trisphosphate 3-phosphatase and protein phosphatase and tensin homolog deleted on chromosome 10	P60484	-13.8
872	PK082-1	PKCg	T514	Protein-serine kinase C gamma	P05129	-14.0

808	PK067	PKA Ca/b	T197	cAMP-dependent protein-serine kinase catalytic subunit alpha/beta	P17612	-14.1
74	NK012	BMX (Etk)	Pan-specific	Bone marrow X protein-tyrosine kinase	P51813	-16.4
796	NN114	PI3KR4	Pan-specific	Phosphoinositide-3-kinase, regulatory subunit 4	Q99570	-17.0
978	NP025	PTP1C	Pan-specific	Protein-tyrosine phosphatase 1C (SHP1, SHPTP1)	Q06124	-17.3
876	PK083	PKCg	T655	Protein-serine kinase C gamma	P05129	-19.0
540	PK038	Kit	Y936	Kit/Steel factor receptor-tyrosine kinase	P10721	-19.5
846	PK075	PKCb1/2	T500	Protein-serine kinase C beta 1/2	P05771	-20.8
104	NK019-1	CAMK2d	Pan-specific	Calcium/calmodulin-dependent protein-serine kinase 2 delta	Q13557	-22.0
102	NK018-2	CAMK2b	Pan-specific	Calcium/calmodulin-dependent protein-serine kinase 2 beta	Q13554	-22.7
782	NK126-2	PDK1	Pan-specific	3-phosphoinositide-dependent protein-serine kinase 1	O15530	-23.3
356	NK065	Fyn	Pan-specific	Fyn proto-oncogene-encoded protein-tyrosine kinase	P06241	-26.0
366	NN045	GNB2L1	Pan-specific	Guanine nucleotide-binding protein beta (receptor for activated C kinase 1 (RACK1))	P63244	-27.6
76	PK003	BMX (Etk)	Y40	Bone marrow X protein-tyrosine kinase	P51813	-27.8
88	PN015	Caldesmon	S789	Caldesmon	Q05682	-28.4
538	PK037	Kit	Y730	Kit/Steel factor receptor-tyrosine kinase	P10721	-28.5
864	NK136	PKCe	Pan-specific	Protein-serine kinase C epsilon	Q02156	-29.3
798	NN090	PI4K2b	Pan-specific	Phosphatidylinositol 4-kinase type 2 beta	Q8TCG2	-31.0
822	PK072-1	PKBa (Akt1)	S473	Protein-serine kinase B alpha	P31749	-31.1
100	NK018-1	CAMK2b	Pan-specific	Calcium/calmodulin-dependent protein-serine kinase 2 beta	Q13554	-31.5
96	PK005-1	CaMK2a	T286	Calcium/calmodulin-dependent protein-serine kinase 2 alpha	Q9UQM7	-32.9
106	NK019-2	CAMK2d	Pan-specific	Calcium/calmodulin-dependent protein-serine kinase 2 delta	Q13557	-33.0
14	PN003-PN004	Adducin a/g	S726/S693	Adducin alpha/gamma (ADD1/3)	P35611	-33.7
108	NK020	CAMK2g	Pan-specific	Calcium/calmodulin-dependent protein-serine	Q13555	-35.0

				kinase 2 gamma		
990	PK097	Pyk2	Y579	Protein-tyrosine kinase 2	P60953	-36.6
854	PK079	PKCd	S645	Protein-serine kinase C delta	Q05655	-41.1
974	PP003	PTEN	S380+S382 +S385	Phosphatidylinositol-3,4,5-trisphosphate 3-phosphatase and protein phosphatase and tensin homolog deleted on chromosome 10	P18031	-41.7
582	PN050-1	MARCKS	S158+S162	Myristoylated alanine-rich protein kinase C substrate	P29966	-41.7
812	NK128	PKA R2a	Pan-specific	cAMP-dependent protein-serine kinase regulatory type 2 subunit alpha	P13861	-42.0
132	NK021	CaMK4	Pan-specific	Calcium/calmodulin-dependent protein-serine kinase 4	Q16566	-42.7
842	PK074	PKCa/b2	T638/T641	Protein-serine kinase C alpha	P17252	-43.1
802	NN091	PI5K2a	Pan-specific	Phosphatidylinositol 4-phosphatase 5-kinase type 2 alpha	P48426	-45.0
800	NK192	PI4KCB	Pan-specific	Phosphatidylinositol 4-kinase, catalytic, beta polypeptide	Q5VWC1	-48.5
72	PN013	BLNK	Y84	B-cell linker protein	O75498	-49.5
94	NK017	CaMK1g	Pan-specific	Calcium/calmodulin-dependent protein-serine kinase 1 gamma	Q96NX5	-50.7
980	NP026	PTP1D	Pan-specific	Protein-tyrosine phosphatase 1D (SHP2, SHPTP2, Syp, PTP2C)	P06493	-57.9
874	PK082-2	PKCg	T514	Protein-serine kinase C gamma	P05129	-65.0
970	NP023	PTEN	Pan-specific	Phosphatidylinositol-3,4,5-trisphosphate 3-phosphatase and protein phosphatase and tensin homolog deleted on chromosome 10	P60484	-69.7
**Akt**						
956	PN062	PRAS40	T246	Proline-rich Akt substrate 40 kDa (Akt1S1)	Q96B36	52.7
476	NK078-2	ILK1	Pan-specific	Integrin-linked protein-serine kinase 1	Q13418	50.8
1030	PK116	mTOR (FRAP)	S2448	Mammalian target of rapamycin (FRAP)	P42345	-1.7
972	PP002	PTEN	S370	Phosphatidylinositol-3,4,5-trisphosphate 3-phosphatase and protein phosphatase and tensin homolog deleted on chromosome 10	P60484	-13.8
474	NK078-1	ILK1	Pan-specific	Integrin-linked protein-serine kinase 1	Q13418	-18.7

1028	NK126-1	PDK1	Pan-specific	3-phosphoinositide-dependent protein-serine kinase 1	O15530	-36.3
974	PP003	PTEN	S380+S382 +S385	Phosphatidylinositol-3,4,5-trisphosphate 3-phosphatase and protein phosphatase and tensin homolog deleted on chromosome 10	P18031	-41.7
970	NP023	PTEN	Pan-specific	Phosphatidylinositol-3,4,5-trisphosphate 3-phosphatase and protein phosphatase and tensin homolog deleted on chromosome 10	P60484	-69.7
**MAPK**						
622	NK103	MEK4 (MAP2K4)	Pan-specific	MAPK/ERK protein-serine kinase 4 (MKK4)	P45985	129.8
578	PN049-PN112-1	MAPKAPK 2a/b	T334	Mitogen-activated protein kinase-activated protein kinase 2 alpha/beta	P49137	100.0
632	NK106	MEK7 (MAP2K7)	Pan-specific	MAPK/ERK protein-serine kinase 7 (MKK7)	O14733	94.6
618	NK101	MEK3 (MAP2K3)	Pan-specific	MAPK/ERK protein-serine kinase 3 (MKK3)	P46734	83.1
310	NK059-1	p38g MAPK (Erk6)	Pan-specific	Mitogen-activated protein-serine kinase p38 gamma (MAPK12)	P53778	80.4
1260		MEK3b (MAP2K3)	Pan-specific	MAPK/ERK protein-serine kinase 3 beta isoform (MKK3 beta)	P46734	76.7
572	NK120-2	p38a MAPK	Pan-specific	Mitogen-activated protein-serine kinase p38 alpha	Q16539	43.3
304	NK057	Erk3	Pan-specific	Extracellular regulated protein-serine kinase 3	Q16659	41.0
92	NK016-2	CaMK1d	Pan-specific	Calcium/calmodulin-dependent protein-serine kinase 1 delta	Q8IU85	38.8
298	PK014-PK015-2	Erk1/2	T202+Y204 /T185+Y18 7	Extracellular regulated protein-serine kinase 1/2 (p44/p42 MAP kinases)	P27361	36.9
308	PK016	Erk5	T218+Y220	Extracellular regulated protein-serine kinase 5 (Big MAP kinase 1 (BMK1))	P53778	34.3
574	NK097	MAPKAPK 2	Pan-specific	Mitogen-activated protein kinase-activated protein kinase 2	P49137	30.5
724	PK060-3	p38a MAPK	T180+Y182	Mitogen-activated protein-serine kinase p38 alpha	Q16539	28.6
630	NK105-2	MEK6 (MAP2K6)	Pan-specific	MAPK/ERK protein-serine kinase 6 (MKK6)	P52564	26.6
636	NK108	MEKK2 (MAP3K2)	Pan-specific	MAPK/ERK kinase kinase 2	Q9Y2U5	23.0
614	PK049	MEK2 (MAP2K2) (human)	T394	MAPK/ERK protein-serine kinase 2 (MKK2) (human)	P36507	22.7

988	NK154	Pyk2	Pan-specific	Protein-tyrosine kinase 2	Q14289	21.7
588	NN067	Mcl1	Pan-specific	Myeloid cell leukemia differentiation protein 1	Q07820	20.4
294	NK055-NK056	Erk1/2	Pan-specific	Extracellular regulated protein-serine kinase 1/2 (p44/p42 MAP kinases)	P27361	20.4
616	PK050	MEK2 (MAP2K2) (mouse)	T394	MAPK/ERK protein-serine kinase 2 (MKK2) (mouse)	P36507	19.5
594	PK047-2	MEK1 (MAP2K1)	S297	MAPK/ERK protein-serine kinase 1 (MKK1)	Q02750	16.8
570	NK120-1	p38a MAPK	Pan-specific	Mitogen-activated protein-serine kinase p38 alpha	Q16539	16.8
626	NK104	MEK5 (MAP2K5)	Pan-specific	MAPK/ERK protein-serine kinase 5 (MKK5)	Q13163	16.5
602	PK048-1	MEK1 (MAP2K1)	T385	MAPK/ERK protein-serine kinase 1 (MKK1)	Q02750	15.1
606	PK048-3	MEK1 (MAP2K1)	T385	MAPK/ERK protein-serine kinase 1 (MKK1)	Q02750	13.5
300	PK014-PK015-3	Erk1/2	T202+Y204 /T185+Y187	Extracellular regulated protein-serine kinase 1/2 (p44/p42 MAP kinases)	P27361	13.0
604	PK048-2	MEK1 (MAP2K1)	T385	MAPK/ERK protein-serine kinase 1 (MKK1)	Q02750	10.0
568	NK096	MAK	Pan-specific	Male germ cell-associated protein-serine kinase	P20794	9.1
650	PK056	MLK3	T277+S281	Mixed-lineage protein-serine kinase 3	Q16584	7.2
598	PK046-2	MEK1 (MAP2K1)	T291	MAPK/ERK protein-serine kinase 1 (MKK1)	Q02750	7.1
592	PK047-1	MEK1 (MAP2K1)	S297	MAPK/ERK protein-serine kinase 1 (MKK1)	Q02750	5.8
600	PK046-3	MEK1 (MAP2K1)	T291	MAPK/ERK protein-serine kinase 1 (MKK1)	Q02750	5.2
742	NP008	PAC1	Pan-specific	Dual specificity MAP kinase protein phosphatase	Q05923	3.2
634	NK107	MEKK1 (MAP3K1)	Pan-specific	MAPK/ERK kinase kinase 1	Q13233	3.1
580	PN049-PN112-2	MAPKAPK 2a/b	T334	Mitogen-activated protein kinase-activated protein kinase 2 alpha/beta	P49137	0.9
624	PK052	MEK4 (MAP2K4)	S257+T261	MAPK/ERK protein-serine kinase 4 (MKK4)	P45985	-3.9
590	NK099	MEK1 (MAP2K1)	Pan-specific	MAPK/ERK protein-serine kinase 1 (MKK1)	Q02750	-6.1
576	PK044	MAPKAPK 2	T222	Mitogen-activated protein kinase-activated protein kinase 2	P49137	-6.9
610	NK100-1	MEK2 (MAP2K2)	Pan-specific	MAPK/ERK protein-serine kinase 2 (MKK2)	P36507	-7.5
586	NK098	MARK	Pan-specific	MAP/microtubule affinity-regulating protein-serine kinase 1	Q9P0L2	-9.5
90	NK016-1	CaMK1d	Pan-specific	Calcium/calmodulin-dependent protein-serine	Q8IU85	-11.1

				kinase 1 delta		
36	NK007	ASK1 (MAP3K5)	Pan-specific	Apoptosis signal regulating protein-serine kinase	Q99683	-12.8
296	PK014-PK015-1	Erk1/2	T202+Y204 /T185+Y187	Extracellular regulated protein-serine kinase 1/2 (p44/p42 MAP kinases)	P27361	-14.4
620	PK051	MEK3/6 (MAP2K3/6)	S189/S207	MAPK/ERK protein-serine kinase 3/6 (MKK3/6)	P46734	-15.1
648	NP007	MKP2	Pan-specific	MAP kinase phosphatase 2 (VH2)	Q13115	-15.2
596	PK046-1	MEK1 (MAP2K1)	T291	MAPK/ERK protein-serine kinase 1 (MKK1)	Q02750	-18.3
726	PK060-4	p38a MAPK	T180+Y182	Mitogen-activated protein-serine kinase p38 alpha	Q16539	-20.5
306	NK058	Erk4	Pan-specific	Extracellular regulated protein-serine kinase 4	Q13164	-21.5
728	NK121	p38d MAPK	Pan-specific	Mitogen-activated protein-serine kinase p38 delta (MAPK13)	O15264	-21.6
4	PN001	4E-BP1	S65	Eukaryotic translation initiation factor 4E binding protein 1 (PHAS1)	Q13541	-22.8
730	NK059-2	p38g MAPK (Erk6)	Pan-specific	Mitogen-activated protein-serine kinase p38 gamma (MAPK12)	P53778	-23.5
654	PK057	Mnk1	T209+T214	MAP kinase-interacting protein-serine kinase 1 (calmodulin-activated)	Q9BUB5	-26.4
628	NK105-1	MEK6 (MAP2K6)	Pan-specific	MAPK/ERK protein-serine kinase 6 (MKK6)	P52564	-26.7
608	PK045-PN007	MEK1/2 (MAP2K1/2) + B23 (NPM)	S218+S222, S4	MAPK/ERK protein-serine kinase 1 (MKK1) + B23 (nucleophosmin, numatrin, nucleolar protein NO38)	Q02750	-27.0
302	NK056	Erk2	Pan-specific	Extracellular regulated protein-serine kinase 2 (p42 MAP kinase)	P28482	-29.7
638	NK109	MEKK4 (MAP3K4)	Pan-specific	MAPK/ERK kinase kinase 4	Q9Y6R4	-30.1
722	PK060-2	p38a MAPK	T180+Y182	Mitogen-activated protein-serine kinase p38 alpha	Q16539	-32.7
718	NK120-3	p38a MAPK	Pan-specific	Mitogen-activated protein-serine kinase p38 alpha	Q16539	-34.2
720	PK060-1	p38a MAPK	T180+Y182	Mitogen-activated protein-serine kinase p38 alpha	Q16539	-34.3
612	NK100-2	MEK2 (MAP2K2)	Pan-specific	MAPK/ERK protein-serine kinase 2 (MKK2)	P36507	-35.6
990	PK097	Pyk2	Y579	Protein-tyrosine kinase 2	P60953	-36.6
360	NK066	GCK	Pan-specific	Germinal centre protein-serine kinase	Q12851	-47.0
646	NP006	MKP1	Pan-specific	MAP kinase phosphatase 1 (CL100, VH1)	P28562	-50.1
656	NK111	Mnk2	Pan-specific	MAP kinase-interacting protein-serine kinase 2	Q9HBH9	-73.7

				(calmodulin-activated)		
			**CREB**			
216	PN024	CREB1	S133	cAMP response element binding protein 1	P16220	122.2
664	PK058	Msk1	S376	Mitogen & stress-activated protein-serine kinase 1	O75582	51.6
1036	PK101-1	RSK1/2	S380/S386	Ribosomal S6 protein-serine kinase 1/2	Q15418	43.4
92	NK016-2	CaMK1d	Pan-specific	Calcium/calmodulin-dependent protein-serine kinase 1 delta	Q8IU85	38.8
38	PN006-1	ATF2	T51+T53	Activating transcription factor 2 (CRE-BP1)	P15336	30.1
1038	PK099	RSK1/2	S221/S227	Ribosomal S6 protein-serine kinase 1/2	Q15418	29.7
1046	PK102	RSK1/2/3	T573/T577/ T570	Ribosomal S6 protein-serine kinase 1/2/3	Q15418	1.9
1044	PK103	RSK1/3	T359/T365	Ribosomal S6 protein-serine kinase 1/3	Q15418	-1.3
1042	PK101-2	RSK1/2	S380/S386	Ribosomal S6 protein-serine kinase 1/2	Q15418	-5.1
40	PN006-2	ATF2	T51+T53	Activating transcription factor 2 (CRE-BP1)	P15336	-8.4
1048	NK166	RSK4	Pan-specific	Ribosomal S6 protein-serine kinase 4 (alpha 6)	Q9UK32	-9.6
90	NK016-1	CaMK1d	Pan-specific	Calcium/calmodulin-dependent protein-serine kinase 1 delta	Q8IU85	-11.1
1032	NK164	RSK1	Pan-specific	Ribosomal S6 protein-serine kinase 1	Q15418	-12.0
1034	NK165	RSK2	Pan-specific	Ribosomal S6 protein-serine kinase 2	P51812	-25.0
214	PN023	CREB1	S129+S133	cAMP response element binding protein 1	P16220	-29.9
1040	PK100	RSK1/2	S363/S369	Ribosomal S6 protein-serine kinase 1/2	Q15418	-39.1
132	NK021	CaMK4	Pan-specific	Calcium/calmodulin-dependent protein-serine kinase 4	Q16566	-42.7
94	NK017	CaMK1g	Pan-specific	Calcium/calmodulin-dependent protein-serine kinase 1 gamma	Q96NX5	-50.7
			**Jun**			
516	PK035-1	JNK	T183	Jun N-terminus protein-serine kinases (stress-activated protein kinase (SAPK)) 1/2/3	P45983	161.2
534	NK089	KHS	Pan-specific	Kinase homologous to SPS1/STE20 (MAP kinase kinase kinase protein-serine kinase 5 (MEKKK5)	Q9Y4K4	97.2
526	PN048-1	Jun	S73	Jun proto-oncogene-encoded	P05412	95.1

				AP1 transcription factor		
514	NK088	JNK	Pan-specific	Jun N-terminus protein-serine kinases (stress-activated protein kinase (SAPK)) 1/2/3	P45983	86.4
664	PK058	Msk1	S376	Mitogen & stress-activated protein-serine kinase 1	O75582	51.6
1232	NN066	Jun	Pan-specific	Jun proto-oncogene-encoded AP1 transcription factor	P05412	49.4
528	PN048-2	Jun	S73	Jun proto-oncogene-encoded AP1 transcription factor	P05412	42.9
518	PK035-2	JNK	T183	Jun N-terminus protein-serine kinases (stress-activated protein kinase (SAPK)) 1/2/3	P45983	31.7
988	NK154	Pyk2	Pan-specific	Protein-tyrosine kinase 2	Q14289	21.7
530	PN048-3	Jun	S73	Jun proto-oncogene-encoded AP1 transcription factor	P05412	0.2
512	NK087	JIK (TAO3)	Pan-specific	STE20-like protein-serine kinase	Q9UHG7	-2.6
522	NK189	JNK2	Pan-specific	Jun N-terminus protein-serine kinases (stress-activated protein kinase (SAPK)) 2	P45984	-24.1
520	PK035-3	JNK	T183+Y185	Jun N-terminus protein-serine kinases (stress-activated protein kinase (SAPK)) 1/2/3	P45983	-35.2
990	PK097	Pyk2	Y579	Protein-tyrosine kinase 2	P60953	-36.6
524	NN066	Jun	S63	Jun proto-oncogene-encoded AP1 transcription factor	P05412	-43.0
			**NFκB**			
1162	NN109-2	TBK1	Pan-specific	Tank-binding protein 1	Q9UHD2	97.3
1266	NK077	IKKg/NEMO	Pan-specific	I-kappa-B kinase gamma/NF-kappa-B essential modulator(NEMO)	Q9Y6K9	81.1
1262	NK157	RIP2/RICK	Pan-specific	Receptor-interacting serine/threonine-protein kinase 2 (RIPK2)	O43353	55.9
466	NK075-3	IKKa	Pan-specific	Inhibitor of NF-kappa-B protein-serine kinase alpha (CHUK)	O15111	38.9
1170	NN110	TRADD	Pan-specific	Tumor necrosis factor receptor type 1 associated DEATH domain protein	Q15628	29.3
464	NK075-2	IKKa	Pan-specific	Inhibitor of NF-kappa-B protein-serine kinase alpha (CHUK)	O15111	18.2
462	NK075-1	IKKa	Pan-specific	Inhibitor of NF-kappa-B protein-serine kinase alpha (CHUK)	O15111	13.2
1204	NN112	XIAP	Pan-specific	X-linked inhibitor of apoptosis protein	P98170	12.9

				(baculoviral IAP repeat-containing 4)		
1160	NN109-1	TBK1	Pan-specific	Tank-binding protein 1	Q9UHD2	6.8
1018	NK158	RIPK1	Pan-specific	Receptor-interacting protein-serine kinase 1	Q13546	2.4
472	NK076-2	IKKb	Pan-specific	Inhibitor of NF-kappa-B protein-serine kinase beta	O14920	-9.0
460	NN065	IkBb	Pan-specific	Inhibitor of NF-kappa-B beta (thyroid receptor interacting protein 9)	Q15653	-19.7
458	NN064	IkBa	Pan-specific	Inhibitor of NF-kappa-B alpha (MAD3)	P25963	-20.5
468	PK030-PK031	IKKa/b	S180/S181	Inhibitor of NF-kappa-B protein-serine kinase alpha (CHUK)/beta	O15111	-23.1
470	NK076-1	IKKb	Pan-specific	Inhibitor of NF-kappa-B protein-serine kinase beta	O14920	-25.9
688	NN071	NFkappaB p65	Pan-specific	NF-kappa-B p65 nuclear transcription factor	Q04206	-46.5
686	NN070	NFkappaB p50	Pan-specific	NF-kappa-B p50 nuclear transcription factor	P19838	-48.9
690	PN053	NFkappaB p65	S276	NF-kappa-B p65 nuclear transcription factor	Q04206	-49.9
			**Orphan**			
220	PN110	Crystallin aB	S45	Crystallin alpha B (heat-shock 20 kDa like-protein)	P02511	276.3
24	NK004	ALS2CR7 (PFTAIRE2)	Pan-specific	Amyotrophic lateral sclerosis 2 chromosomal region candidate gene protein-serine kinase 7	Q96Q40	121.7
1206	NK186	Yes	Pan-specific	Yamaguchi sarcoma proto-oncogene-encoded tyrosine kinase	P07947	110.2
218	PN025	Crystallin aB	S19	Crystallin alpha B (heat-shock 20 kDa like-protein)	P02511	91.4
1070	PP004	SHP2	S576	Protein-tyrosine phosphatase 1D (SHP2, SHPTP2, Syp, PTP2C)	Q06124	73.5
960	NK149	PRK2	Pan-specific	Protein kinase C-related protein-serine kinase 2	Q16513	66.2
776	PK063	PDGFRa	Y754	Platelet-derived growth factor receptor kinase alpha	P16234	52.1
1268	NK115	MST3	Pan-specific	Mammalian STE20-like protein-serine kinase 3	Q9Y6E0	48.5
1080	PN076	Smad2	S465+S467	SMA- and mothers against decapentaplegic homolog 2	Q15796	39.0
48	NK010	Axl	Pan-specific	Axl proto-oncogene-encoded protein-tyrosine kinase	P30530	35.2
660	PN051-1	MLC (MLRC2)	S20	Myosin regulatory light chain 2, smooth muscle isoform	P19105	24.3

774	PK062	PDGFRa	Y742	Platelet-derived growth factor receptor kinase alpha	P16234	19.1
1088	PN077	SOX9	S181	SRY (sex determining region Y)-box 9 (campomelic dysplasia, autosomal sex-reversal)	P48436	15.7
756	NN085-1	PARP1	Pan-specific	Poly [ADP-ribose] polymerase 1 (ADPRT)	P09874	14.6
1098	PK108	Src	Y529	Src proto-oncogene-encoded protein-tyrosine kinase	P12931	14.1
312	NN040	ERP57	Pan-specific	ER protein 57 kDa (protein disulfide isomerase-associated 3; 58 kDa glucose regulated protein)	P30101	12.3
1200	NK184	Vrk1	Pan-specific	Vaccinia related protein-serine kinase 1	Q99986	10.9
772	NK125	PCTK1 (PCTAIRE)	Pan-specific	PCTAIRE-1 protein-serine kinase	Q00536	9.9
280	PN097	eNos	T495	Nitric-oxide synthase, endothelial	P29474	7.2
1096	PK107	Src	Y418	Src proto-oncogene-encoded protein-tyrosine kinase	P12931	4.8
1094	NK172	Src	Pan-specific	Src proto-oncogene-encoded protein-tyrosine kinase	P12931	4.5
958	PK095-PK096	PRK1/2 (PKN1/2)	T774	Protein kinase C-related protein-serine kinase 1/2	Q16512	-1.1
32	NN122	APG2	Pan-specific	Hsp 70-related heat shock protein 4 (HSP70RY)	P34932	-2.2
248	NK048	DNAPK	Pan-specific	DNA-activated protein-serine kinase	P78527	-5.5
1064	NK169	S6Kb (p70 S6Kb)	Pan-specific	p70 ribosomal protein-serine S6 kinase beta	Q9UBS0	-6.4
1128	NK173	STK33	Pan-specific	FLJ35932 protein-serine kinase	Q8NEF5	-15.7
80	NK013	BRD2	Pan-specific	Bromodomain-containing protein-serine kinase 2	P25440	-15.7
314	NN041	ERP72	Pan-specific	ER protein 72 kDa (protein disulfide isomerase-associated 4)	P13667	-15.7
200	NK040	CK1g2	Pan-specific	Casein protein-serine kinase 1 gamma 2	P78368	-18.7
1078	PN075	Smad1/5/9	S463+S465/ S463+S465/ S465+S467	SMA- and mothers against decapentaplegic homologs 1/5/9	Q15797	-19.1
1194	NP030	VHR	Pan-specific	Dual specificity protein phosphatase 3	P51452	-20.8
282	NK053	EphA1	Pan-specific	Ephrin type-A receptor 1 protein-tyrosine kinase	P21709	-20.9
1084	NN098	SOD (Cu/Zn)	Pan-specific	Superoxide dismutase 1	P00441	-20.9
758	NN085-2	PARP1	Pan-specific	Poly [ADP-ribose] polymerase 1 (ADPRT)	P09874	-25.4
202	NK041	CK2a	Pan-specific	Casein protein-serine kinase 2 alpha/ alpha prime	P68400	-25.7
984	NP027	PTP-PEST	Pan-specific	Protein-tyrosine phosphatase with PEST sequences	Q15119	-27.8

				(PTPG1, PTPN12)		
658	NK112	Mos	Pan-specific	Moloney sarcoma oncogene-encoded protein-serine kinase	P00540	-32.7
780	PK065	PDGFRb	Y716	Platelet-derived growth factor receptor kinase beta	P09619	-35.4
1016	PN072	Ret	S696	Ret receptor-tyrosine kinase	Q13546	-37.6
778	PK064	PDGFRa/b	Y572+Y574/Y579+Y581	Platelet-derived growth factor receptor kinase alpha/beta	P16234	-49.6
342	NK061	Fes	Pan-specific	Fes/Fps protein-tyrosine kinase	P07332	-61.5
